# Zinc limitation triggers anticipatory adaptations in *Mycobacterium tuberculosis*

**DOI:** 10.1371/journal.ppat.1009570

**Published:** 2021-05-14

**Authors:** Allexa Dow, Preeti Sule, Timothy J. O’Donnell, Andrew Burger, Joshua T. Mattila, Brandi Antonio, Kevin Vergara, Endrei Marcantonio, L. Garry Adams, Nicholas James, Philip G. Williams, Jeffrey D. Cirillo, Sladjana Prisic

**Affiliations:** 1 School of Life Sciences, University of Hawaiʻi at Mānoa, Honolulu, Hawaii, United States of America; 2 Microbial Pathogenesis and Immunology, Texas A&M University Health, Bryan, Texas, United States of America; 3 Department of Chemistry, University of Hawaiʻi at Mānoa, Honolulu, Hawaii, United States of America; 4 School of Ocean and Earth Science and Technology, University of Hawaiʻi at Mānoa, Honolulu, Hawaii, United States of America; 5 Department of Infectious Diseases and Microbiology, University of Pittsburgh, Pittsburgh, Pennsylvania, United States of America; 6 Department of Veterinary Pathobiology, Texas A&M University, College Station, Texas, United States of America; 7 Department of Cell and Molecular Biology, John A. Burns School of Medicine, Honolulu, Hawaii, United States of America; University of Massachusetts Medical School, UNITED STATES

## Abstract

*Mycobacterium tuberculosis* (*Mtb*) has complex and dynamic interactions with the human host, and subpopulations of *Mtb* that emerge during infection can influence disease outcomes. This study implicates zinc ion (Zn^2+^) availability as a likely driver of bacterial phenotypic heterogeneity *in vivo*. Zn^2+^ sequestration is part of “nutritional immunity”, where the immune system limits micronutrients to control pathogen growth, but this defense mechanism seems to be ineffective in controlling *Mtb* infection. Nonetheless, Zn^2+^-limitation is an environmental cue sensed by *Mtb*, as calprotectin triggers the zinc uptake regulator (Zur) regulon response *in vitro* and co-localizes with Zn^2+^-limited *Mtb in vivo*. Prolonged Zn^2+^ limitation leads to numerous physiological changes *in vitro*, including differential expression of certain antigens, alterations in lipid metabolism and distinct cell surface morphology. Furthermore, *Mtb* enduring limited Zn^2+^ employ defensive measures to fight oxidative stress, by increasing expression of proteins involved in DNA repair and antioxidant activity, including well described virulence factors KatG and AhpC, along with altered utilization of redox cofactors. Here, we propose a model in which prolonged Zn^2+^ limitation defines a population of *Mtb* with anticipatory adaptations against impending immune attack, based on the evidence that Zn^2+^-limited *Mtb* are more resistant to oxidative stress and exhibit increased survival and induce more severe pulmonary granulomas in mice. Considering that extracellular *Mtb* may transit through the Zn^2+^-limited caseum before infecting naïve immune cells or upon host-to-host transmission, the resulting phenotypic heterogeneity driven by varied Zn^2+^ availability likely plays a key role during early interactions with host cells.

## Author summary

*Mycobacterium tuberculosis* (*Mtb*), the causative agent of tuberculosis (TB), has plagued humanity for millennia and remains the world’s deadliest bacterium today. Bacterial heterogeneity is one of the most important characteristics of *Mtb* that complicates TB treatment. Access to zinc ion (Zn^2+^) may influence bacterial heterogeneity, considering microenvironments developed during TB create a perpetual cycle exposing *Mtb* to high and low concentrations of Zn^2+^. Here we show that Zn^2+^ limitation drives changes in gene expression patterns of well described virulence factors in *Mtb*, and Zn^2+^-limited *Mtb* show increased resistance to oxidative stress and increased replication *in vivo*. Our results suggest that host-pathogen interactions are influenced by pre-exposure of *Mtb* to Zn^2+^ and mycobacteria that transit through a Zn^2+^-depleted microenvironment are primed to withstand impending oxidative stress upon subsequent contact with immune cells in the same, or a naïve host. Considering that the standard mycobacterial media recapitulates a Zn^2+^-replete environment, the Zn^2+^-dependent phenotype of the pathogen may confound our fundamental understanding of initial interactions between *Mtb* and immune cells.

## Introduction

The success of *Mycobacterium tuberculosis* (*Mtb*) as a human pathogen is enabled by a genetic arsenal that allows it to withstand a myriad of immune defenses, survive in diverse host environments, and establish persistent infection [1]. Disease outcome is influenced by subpopulations of *Mtb* arising within heterogeneous microenvironments *in vivo*, but specific cues from the host leading to their development are mostly unknown [2–5].

One cue that triggers physiological adaptation in many pathogens is the limitation of essential micronutrients, *e*.*g*., free zinc ion (Zn^2+^), a strategy known as ‘nutritional immunity’ [6]. Indeed, drastic changes in Zn^2+^ concentration [Zn^2+^] are experienced by *Mtb* throughout infection. Zn^2+^ rapidly accumulates in phagosomes [7], but *Mtb* is protected from zinc poisoning through upregulation of heavy metal efflux P_1_-type ATPase as it colonizes the intracellular niche [8]. Infected macrophages release pro-inflammatory cytokines which recruit immune cells to the site of infection forming the hallmark pathology of TB, the granuloma [1,9]. As TB disease progresses, neutrophils infiltrate granulomas, promoting necrosis of unresolved *Mtb*-infected immune cells [3,10]. Necrotic cells release their contents, including *Mtb*, into the extracellular milieu, a microenvironment rich in neutrophil-derived Zn^2+^ and Mn^2+^-binding protein calprotectin (CP), part of the ‘nutritional immunity’ host response [10]. Therefore, throughout infection *Mtb* may transit between high [Zn^2+^] inside macrophages and low [Zn^2+^] in CP-rich caseum. The spectrum of diverse immunopathologies of granulomas and necrotic cavities give rise to distinct subpopulations of *Mtb* that exist simultaneously within the host [3,5], and [Zn^2+^] may be a contributing factor in their development. Further, [Zn^2+^]-dependent physiological changes may affect interactions with the immune system, thus influencing disease progression and response to treatment.

As with many other bacteria, *Mtb* has a Zn^2+^-responsive transcriptional repressor–zinc uptake regulator (Zur) that controls 21 genes with upregulated expression during Zn^2+^-limiting conditions [11]. Zn^2+^ limitation is likely a cue sensed by *Mtb* during infection, considering upregulation of Zur-regulated genes (*e*.*g*., genes in the *altRP* operon [12]) detected in *Mtb* from human sputum [13,14]. However, the Zur regulon may be just one aspect of the global response to Zn^2+^ availability, as suggested by transcriptomic studies in other Zn^2+^-limited bacteria [15]. Since [Zn^2+^] is tied to specific microenvironments *in vivo*, it may cue *Mtb* to trigger adaptive responses beyond maintaining zinc homeostasis. We hypothesize that *Mtb* has a dynamic global response enabling endurance through periods of prolonged Zn^2+^ limitation and reason that Zn^2+^-limited *Mtb*, such as those found in sputum or exposed to CP-rich regions in the extracellular microenvironment, are phenotypically distinct from *Mtb* residing in a Zn^2+^-replete niche.

In this study, we show that *Mtb* enduring Zn^2+^-limited and Zn^2+^-replete environments have distinct signatures, suggesting that [Zn^2+^] may likewise delineate subpopulations of *Mtb in vivo*. We use biochemical and multi-omics approaches to describe the effect of prolonged Zn^2+^ limitation at a global scale in *Mtb* grown *in vitro*. The analysis of Zn^2+^-limited *Mtb* revealed a response that goes beyond the Zur regulon, including activation of the oxidative stress response, altered utilization of reducing cofactors, changes in the lipidome and a distinct cell surface morphology. In addition, Zn^2+^-limited *Mtb* are more resistant to oxidative stress and more sensitive to the prodrug isoniazid compared to Zn^2+^-replete *Mtb*. Finally, in an aerosol mouse model of infection, Zn^2+^-limited inoculum exhibited greater bacterial burden and pulmonary granulomas than Zn^2+^-replete inoculum. Together these findings define a novel adaptive mechanism employed by *Mtb* during Zn^2+^ limitation that triggers formation of a distinct population that may play a role in TB pathogenesis.

## Results

### *Mtb* responds to Zn^2+^ limitation *in vivo* and *in vitro*

**Fig 1:**
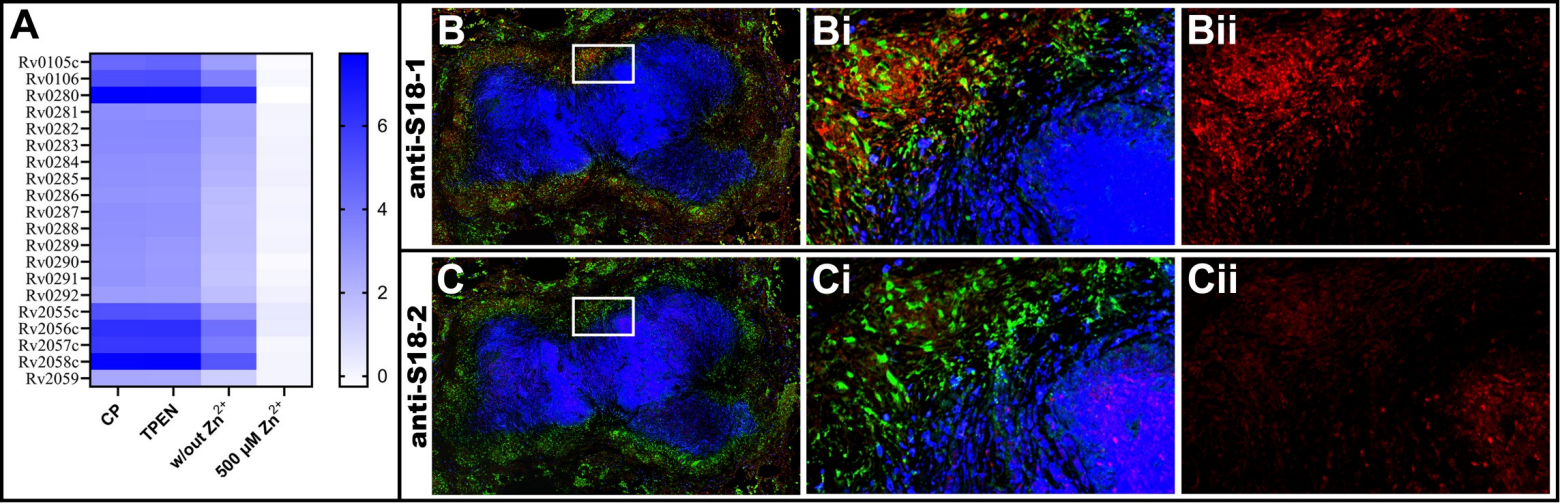
CP upregulates the Zur regulon *in vitro* and detection of Zur-regulated protein expression in regions containing CP *in vivo*. (A) Heatmap showing log_2_FC values for genes in the Zur regulon after growth in media without added Zn^2+^ followed by short-term exposure to CP, TPEN, 6 μM Zn^2+^ and 500 μM Zn^2+^. In each condition the change in expression (log_2_FC) is relative to the 6 μM Zn^2+^ condition. Log_2_FC expression values are calculated from the normalized expression counts of biological replicates (n = 3). Confocal microscopy images of serial sections of lung granulomas at 20X magnification stained for *Mtb* ribosomal proteins with polyclonal anti-S18-1 (B) and anti-S18-2 (C) antibodies (*red*), CD68+ macrophages (*green*) and CP (*blue*). The field in B and C bound by the white box is magnified in panels Bi-ii and Ci-ii and corresponds to a region containing both CD68+ epithelioid macrophages and CP-stained neutrophils and caseum, as well as distribution of *Mtb* S18-1 (Bi) and S18-2 (Ci). Single channel images from staining with antibodies specific to *Mtb* ribosomal proteins only is shown for S18-1 (Bii) and S18-2 (Cii).

Accumulation of CP in the caseum of necrotic granulomas is a marked feature of human pulmonary tuberculosis [16]. Cavitation of necrotic granulomas provides the route for *Mtb* transmission into the airways and out of the host via sputum [3]. Accordingly, we detected CP in sputum from patients with active TB (S1 Table), suggesting that extracellular *Mtb* in the caseum and sputum are in contact with CP. CP has been shown to induce *altRP* expression *in vitro* [12] and the increased *altRP* expression detected in *Mtb* from human sputum suggests this subpopulation is experiencing Zn^2+^-limitation due to Zur-regulation of the *altRP* operon [13,14]. However, other environmental stressors may also upregulate *altRP* expression [17], so to validate the notion that increased *altRP* expression is in response to Zn^2+^-limiting conditions sensed by *Mtb* in contact with Zn^2+^-binding CP, we further investigated expression of all genes in the *Mtb* Zur regulon in response to CP *in vitro*. As expected, we saw significant upregulation of the Zur regulon (except Rv2060 for which we did not detect gene expression), including the *altRP* operon (Rv2055c-Rv2058c), which was one of the most highly expressed features in response to CP (Fig 1A). In agreement, recent high throughput sequencing of *Mtb* transcriptomes from human sputum reveal upregulation of Zur regulon just as we have shown for *Mtb* exposed to CP [18]. The clear evidence that CP induces expression of Zur-regulated genes demonstrates that *Mtb* in contact with CP experience Zn^2+^ limitation, and the observation that *Mtb* from sputum also have increased expression of Zur regulon suggests this subpopulation of bacteria is likely enduring Zn^2+^-limitation.

Beyond the Zn^2+^-limited signature of *Mtb* from sputum and the observed increase in *altRP* expression from *Mtb* in artificial granulomas in mice [19], there is sparse information regarding the existence or localization of Zn^2+^-limited *Mtb in vivo*. Because the caseum is a microenvironment rich in CP [10], we predict that extracellular *Mtb* in necrotic granulomas (caseum) have limited access to Zn^2+^ and will be delineated from Zn^2+^-replete bacteria by expression of genes in the Zur regulon. Therefore, we hypothesized that S18-2 protein, one of the products of the Zur-regulated *altRP* operon, will be observed in the Zn^2+^-limited, CP-rich cores of necrotic granulomas. To test this hypothesis, we stained serial sections of necrotic granulomas from experimentally infected cynomolgus macaques [10] with polyclonal antibodies targeting either S18-1 or S18-2 ribosomal proteins in combination with antibodies against CD68 and CP to identify macrophages and CP-rich necrotic regions, respectively (Fig 1B and 1C).

The antibody against S18-1 protein is useful as marker for the presence of *Mtb* antigens, but does not delineate subpopulations of *Mtb*, considering S18-1 protein is detected in *Mtb* cultures with and without S18-2 protein expression [12] and S18-1 antibody has non-specific binding to numerous other *Mtb* antigens (S1 Fig). The signal from S18-1 antibody associated with the macrophage rich cellular ring surrounding the necrotic core of the granuloma, consistent with the description of this microenvironment as one that contains intracellular bacteria [10] (Fig 1B). The signal from S18-2 antibody, which is specific for S18-2 protein and is selectively detected in Zn^2+^-limited *Mtb* cultures [12] (S1 Fig), was associated with the signal from CP found within the acellular core (*i*.*e*., caseum) of necrotic granulomas, a microenvironment that contains extracellular bacteria [10] (Fig 1C). Although we could not co-stain for individual bacteria because of technical limitations with our staining protocol, extracellular bacilli are detected in the caseum of necrotic macaque granulomas [10] and intact acid-fast bacteria are observed in association with necrotic cells and debris in the caseum of patients with post-primary TB [20], suggesting that the signal detected from S18-2 is from extracellular bacteria in the CP-rich caseum.

### *Mtb* growth during prolonged Zn^2+^ limitation *in vitro*

We seek to understand how *Mtb* responds to Zn^2+^ limited conditions, such as what may occur during the transition from intracellular to CP-rich extracellular microenvironments. Expression of Zur-regulated genes delineates a subpopulation of *Mtb* in the CP-rich, Zn^2+^ limited microenvironment *in vivo*, so we reasoned that, to analyze the Zn^2+^-dependent phenotype of *Mtb*, expression of Zur-regulated genes may be used as a proxy to establish Zn^2+^-limiting conditions *in vitro*. Beyond the small-scale study of Zur expression in *Mtb* exposed to CP, it was not feasible to use recombinant CP to study the response to prolonged Zn^2+^-limitation, so we also followed expression of Zur regulon in *Mtb* exposed to *N*,*N*,*N′*,*N′*-tetrakis(2-pyridinylmethyl)-1,2-ethanediamine (TPEN), a synthetic Zn chelator that has a precedence for being used to study cellular responses to Zn^2+^ limitation. Recently, it was shown that use of TPEN leads to Zn^2+^-starved mycobacteria that progress from ribosome remodeling (*i*.*e*., expression of AltRPs and their incorporation into ribosomes) to hibernation and growth arrest [21]. Furthermore, although considered a Zn^2+^-specific chelator, TPEN has been shown to influence the transcriptional profile of bacteria beyond changes relevant to Zn^2+^ alone [22]. Indeed, we observed induction of the Zur regulon in *Mtb* exposed to TPEN (Fig 1A), however we also observed a significant increase in the expression of genes involved in mycobactin synthesis (*i*.*e*., *mbt* operon) in TPEN vs. CP conditions (S2 Table), suggesting that use of TPEN intertwines the response to Zn^2+^ and iron limitation in *Mtb*. Growth of *Mtb* in chemically defined Sauton’s medium without addition of Zn^2+^ also upregulated the Zur regulon compared to *Mtb* grown in presence of the standard 6 μM Zn^2+^ supplementation (Fig 1A). Adding Zn^2+^ to the levels found in phagosomes [7], *i*.*e*., 500 μM Zn^2+^, did suppress the Zur regulon, but not significantly more than when *Mtb* was grown with 6 μM Zn^2+^ (Fig 1A). Therefore, we decided to use 6 μM Zn^2+^ supplemented Sauton’s medium as Zn^2+^ replete medium (ZRM) and Sauton’s medium without added Zn^2+^ as Zn^2+^ limited medium (ZLM) for all our subsequent experiments. This Zn^2+^-limiting condition (*i*.*e*., bacterial growth in ZLM) induces mycobacterial ribosome remodeling, but maintains translational activity of alternative ribosomes [23] and was the ideal condition to study the response to prolonged Zn^2+^-limitation in *Mtb*. To detect the onset of Zn^2+^-limitation in ZLM, we created a transcriptional reporter strain with the Zur-regulated *altRP* operon promoter fused to mCherry fluorescent protein (P_altRP_-mCherry), as fluorescence from this strain has previously been shown to correlate with *altRP* gene and protein expression in *Mtb* [12].

We monitored growth of virulent *Mtb* (strain H37Rv) and the attenuated double auxotroph (strain mc^2^ 6206, a safe and suitable model organism for *Mtb* research [24]) in parallel with fluorescence from the P_altRP_-mCherry reporter in both *Mtb* strains in ZLM and ZRM. Fluorescence was first detected in *Mtb* (strains H37Rv and mc^2^ 6206) after 4 days of growth in ZLM, indicating the onset of Zn^2+^ limitation in ZLM (S2A and S2B Fig). Thus, we determined that the response to persistent Zn^2+^ limitation in *Mtb* can be studied using growth in ZLM, and while we do not know the specific [Zn^2+^] at which Zur-regulated *altRP* expression is induced, omitting use of Zn^2+^-chelation enables us to conclude it is below the concentration of [Zn^2+^] in ZLM which was determined by ICP-MS analysis to be 115 ± 37 nM (average and standard deviation from three independent media preparations). To investigate adaptations employed by *Mtb* during prolonged Zn^2+^ limitation, cultures grown in ZLM were compared to cultures grown in ZRM. We recently described a unique morphogenesis of the non-pathogenic mycobacteria *Mycobacterium smegmatis* (*Msm*) marked by cell elongation upon Zn^2+^ limitation [25], however, in contrast to *Msm*, we did not observe any growth-related impairments due to Zn^2+^ limitation (S2C and S2D Fig), nor did we observe obvious changes in cell length when *Mtb* was grown in ZLM (S2E and S2F Fig). Based on observations of P_altRP_-mCherry fluorescence in ZLM (S2A and S2B Fig), while potentially maximizing the effect of metabolic and structural remodeling due to Zn^2+^ limitation to occur at all levels of cellular structures, we chose to further investigate the expression profiles and physiology of *Mtb* in late log phase (*i*.*e*., after 10 days of growth) in ZLM and ZRM.

### Global changes in the transcriptome of Zn^2+^-limited *Mtb*

**Fig 2:**
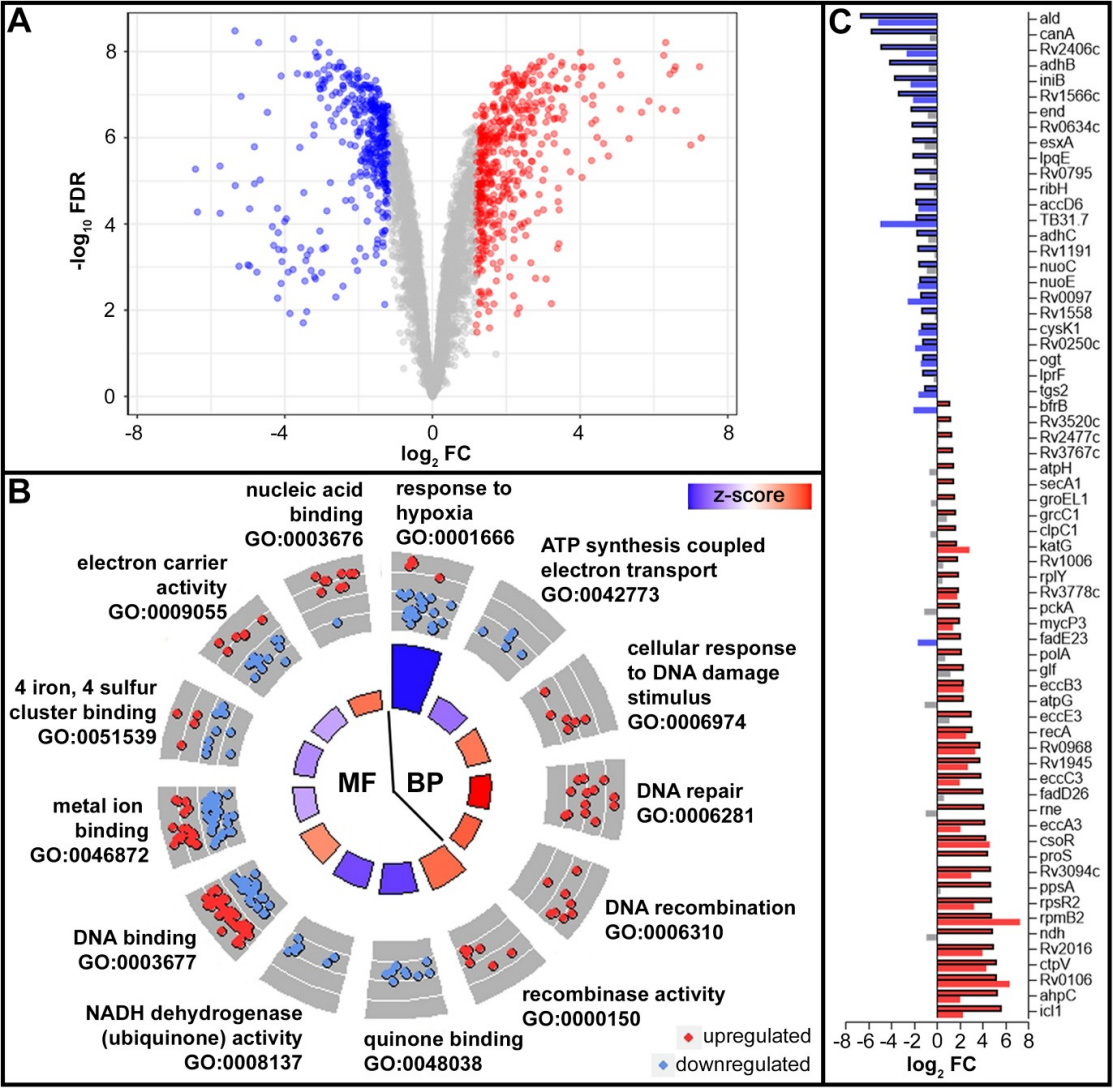
Adaptive response to Zn^2+^ limitation in *Mtb*. (A) Volcano plot of DE genes (absFC >2, FDR <0.05) in *Mtb* H37Rv in ZLM vs. ZRM. Genes colored in red, blue, and grey are significantly upregulated, downregulated or not significantly regulated in ZLM vs. ZRM, respectively. (B) Circle plot showing GO terms of biological processes (BP) and molecular functions (MF) significantly enriched in the list of DE genes in ZLM. Each pie slice of the circle is labeled with the enriched GO term, the outer circle shows a scatter plot of DE genes with the given GO annotation and their log_2_FC values where red circles display upregulated and blue circles downregulated genes in ZLM vs. ZRM. The color of bars in the inner circle indicates whether the given biological process is more likely to be increased (red) or decreased (blue) in the dataset and the height represents the -log_10_(FDR) for the enriched term (larger bars have smaller FDR). (C) Horizontal bar graph showing the overlap between transcript and protein expression for the 65 DE proteins identified in ZLM (absFC >1.5, FDR <0.05). For each gene, the bar on top (black outline) represents the log_2_FC value of the protein and the bar below it (no outline) represents the log_2_FC value of its transcript. Red bars show upregulation and blue bars show downregulation of the protein or transcript in ZLM vs. ZRM while grey bars represent no significant differential expression for transcripts. RNA and proteins for DE analysis were isolated from the same cultures of day 10 cells from ZRM (n = 3) and ZLM (n = 3).

To elucidate the global response employed upon prolonged Zn^2+^ limitation, we analyzed the transcriptomes of Zn^2+^-replete and Zn^2+^-limited *Mtb* H37Rv after 10 days of growth in ZRM and ZLM, respectively, using RNA sequencing (RNAseq). Analyzing the transcriptomes from ZLM and ZRM with RNAseq yielded identification of over 99% of the coding and non-coding RNAs in the *Mtb* genome, leading to identification of 379 upregulated and 346 downregulated genes in Zn^2+^ limited condition, *i*.*e*., ZLM vs. ZRM (Fig 2A). When visualized on a multidimensional scaling plot, we found that [Zn^2+^] was the first dimension describing over 90% of variation in the data, with the second dimension representing intra-sample (ZRM n = 3, ZLM n = 3) variation having a much smaller effect (S3A Fig).

While we observed significant upregulation of most Zur-regulated genes (S3 Table), there were numerous other differentially expressed (DE) genes greatly surpassing the induction level of genes in the Zur regulon (S4 Table). Filtering DE genes by those with the greatest level of upregulation in ZLM yielded a surprising observation that most tRNAs are highly upregulated, with tRNA-leuV being the most highly expressed feature in ZLM (S4 Table). In fact, 33/45 tRNAs in the *Mtb* genome are identified as DE genes upregulated in ZLM whereas only one tRNA was downregulated (S4 Fig).

To better understand the functionality of genes that are differentially regulated during prolonged Zn^2+^ limitation in *Mtb*, we conducted an enrichment analysis for gene ontology (GO) terms on the list of DE genes in ZLM. The enrichment analysis showed 65% of the DE genes had at least one functionally enriched GO term which yielded 15 functional categories that were significantly enriched in the list of DE genes (S5A Fig). Fig 2B shows a circle plot of genes that are significantly enriched in the GO analysis. The circle plot shows specific downregulation of genes involved in the hypoxic response, electron transport and quinone binding as well as metal ion binding function during Zn^2+^ limitation. On the other hand, there was a strong upregulation of genes involved in DNA repair and synthesis, recombination and the general response to DNA damage stimulus during Zn^2+^ limitation. Similar results are apparent when assigning DE genes to pathways as defined by KEGG (S6 Fig and S6 and S7 Tables). These results suggest that energy production, electron flow and oxygen consumption are reduced while processes involved with DNA repair are increased in *Mtb* during prolonged periods of [Zn^2+^] limitation.

### Changes in the proteome mirrors transcriptome during Zn^2+^ limitation in *Mtb*

The abundance of an mRNA molecule and the protein it codes for are not always correlated; post-transcriptional or post-translational processes affect protein abundance [26,27]. Moreover, the shift in translational machinery from primary to alternative ribosomes upon Zn^2+^ limitation in *Mtb* creates a differential population of ribosomes, and this switch could alter ribosomal specificity [12]. Indeed, selective translation from alternative ribosomes in *Msm* has been recently described [28]. If alternative ribosomes in *Mtb* also exhibit selective translation, these changes may be undetectable at the transcript level but ultimately affect the proteome. Therefore, we also probed the proteomes of *Mtb* in ZLM and ZRM to investigate proteins that may be differentially correlated with transcript abundance and to define the core response to Zn^2+^ limitation conserved between the transcriptome and proteome.

We isolated proteins from the same cultures of *Mtb* H37Rv used for RNAseq and coordinately analyzed the proteomes of cultures from ZLM and ZRM with label-free quantitation using mass spectrometry. As is typical with shotgun proteomics, coverage of the genome was much lower than with RNAseq; only about half of theoretical proteins were identified using spectral counting (SpC) and removing proteins with low expression resulted in 738 proteins used for differential expression analysis, among which 65 were identified as DE proteins with 25 being upregulated and 40 being downregulated in ZLM (Fig 2C and S5 Table). When visualized on a multidimensional scaling plot, we found that [Zn^2+^] explains the largest portion of variation in the data (nearly 80%), with intra-sample variation having a much smaller effect (S3B Fig). GO enrichment of DE proteins yielded six significantly enriched categories with “cell wall” being the only category present in the GO analysis of both genes and proteins (S5 Fig). However, there was considerable overlap between DE genes and DE proteins, with half of the DE proteins (33/65) correlating with a DE gene and in only two of these cases (FadE23, BfrB) was the regulation level discordant (Fig 2C). We reasoned that DE genes and DE proteins that follow the same level of regulation (n = 31) represent a robust biological indication of the response to Zn^2+^ limitation and this list is given in Table 1.

**Table 1:** Conserved response to Zn^2+^ limitation in *Mtb* as defined by the concordance of DE genes and proteins in ZLM vs. ZRM.

H37Rv Locus	Gene name	Product (Protein name)	logFC RNA	logFC Protein
Rv2058c	rpmB2	50S ribosomal protein L28 (L28-2)	7.24	4.83
Rv0106	Rv0106	Conserved hypothetical protein	6.32	5.26
Rv0967	csoR	Copper-sensitive operon repressor (CsoR)	4.58	4.32
Rv0969	ctpV	Probable metal cation transporter P-type ATPase (CtpV)	4.30	5.20
Rv2016	Rv2016	Hypothetical protein	3.95	4.96
Rv0968	Rv0968	Conserved protein	3.31	3.81
Rv2055c	rpsR2	30S ribosomal protein S18 (S18-2)	3.21	4.80
Rv3094c	Rv3094c	Conserved hypothetical protein	2.95	4.70
Rv1908c	katG	Catalase-peroxidase-peroxynitritase (KatG)	2.81	1.73
Rv1945	Rv1945	Conserved hypothetical protein	2.68	3.81
Rv2737c	recA	Recombinase A protein (RecA)	2.51	3.07
Rv0283	eccB3	ESX-3 type VII secretion system protein (EccB3)	2.28	2.32
Rv0467	icl1	Isocitrate lyase (Icl)	2.24	5.67
Rv0282	eccA3	ESX-3 type VII secretion system protein (EccA3)	2.02	4.18
Rv2428	ahpC	Alkyl hydroperoxide reductase C (AhpC)	2.00	5.31
Rv0284	eccC3	ESX-3 type VII secretion system protein (EccC3)	1.96	3.82
Rv3778c	Rv3778c	Possible aminotransferase	1.75	1.94
Rv0291	mycP3	Probable membrane-anchored mycosin (MycP3)	1.36	2.03
Rv3809c	glf	UDP-galactopyranose mutase (Glf)	1.16	2.29
Rv1316c	ogt	Methylated-DNA—protein-cysteine methyltransferase (Ogt)	-1.45	-1.36
Rv2334	cysK1	Cysteine synthase a (CysK1)	-1.65	-1.43
Rv3734c	tgs2	Putative triacylglycerol synthase (Tgs2)	-1.65	-1.13
Rv2247	accD6	Acetyl/propionyl-CoA carboxylase, beta subunit (AccD6)	-1.66	-1.97
Rv3149	nuoE	Probable NADH dehydrogenase I, chain E (NuoE)	-1.72	-1.62
Rv0250c	Rv0250c	Conserved protein	-1.94	-1.37
Rv1566c	Rv1566c	Possible Inv protein	-2.13	-3.44
Rv0341	iniB	Isoniazid inductible gene protein (IniB)	-2.35	-3.82
Rv0097	Rv0097	Possible oxidoreductase	-2.59	-1.50
Rv2406c	Rv2406c	Conserved protein	-2.68	-5.02
Rv2623	TB31.7	Universal stress protein family protein (TB31.7)	-5.00	-1.93
Rv2780	ald	Secreted L-alanine dehydrogenase, 40 kDa antigen (Ald)	-5.19	-6.80

### Zn^2+^-limited *Mtb* maintain redox homeostasis

Zn^2+^, a potent antioxidant [29], assists in preservation of cellular redox homeostasis, and Zn^2+^ deficiency is a condition widely associated with oxidative stress [30,31]. Reactive oxygen species (ROS), produced during oxidative stress, are associated with increased levels of protein and lipid oxidation and DNA damage [32]. As such, mechanisms involved in detoxification of ROS have been reported as an adaptive response during Zn^2+^ depletion [31,33]. Based on these observations and the results obtained from the multi-omics analysis, we postulated that Zn^2+^ limited *Mtb* experience increased exposure to ROS. Specific clues in the data were selective upregulation of some key features in ZLM including the antioxidants catalase (KatG) and alkylhydroperoxide reductase (AhpC) and many genes involved in DNA replication, repair and response to DNA damage (Fig 2 and Table 1), consistent with observations in *Mtb* treated with oxidizing agents [34].

**Fig 3:**
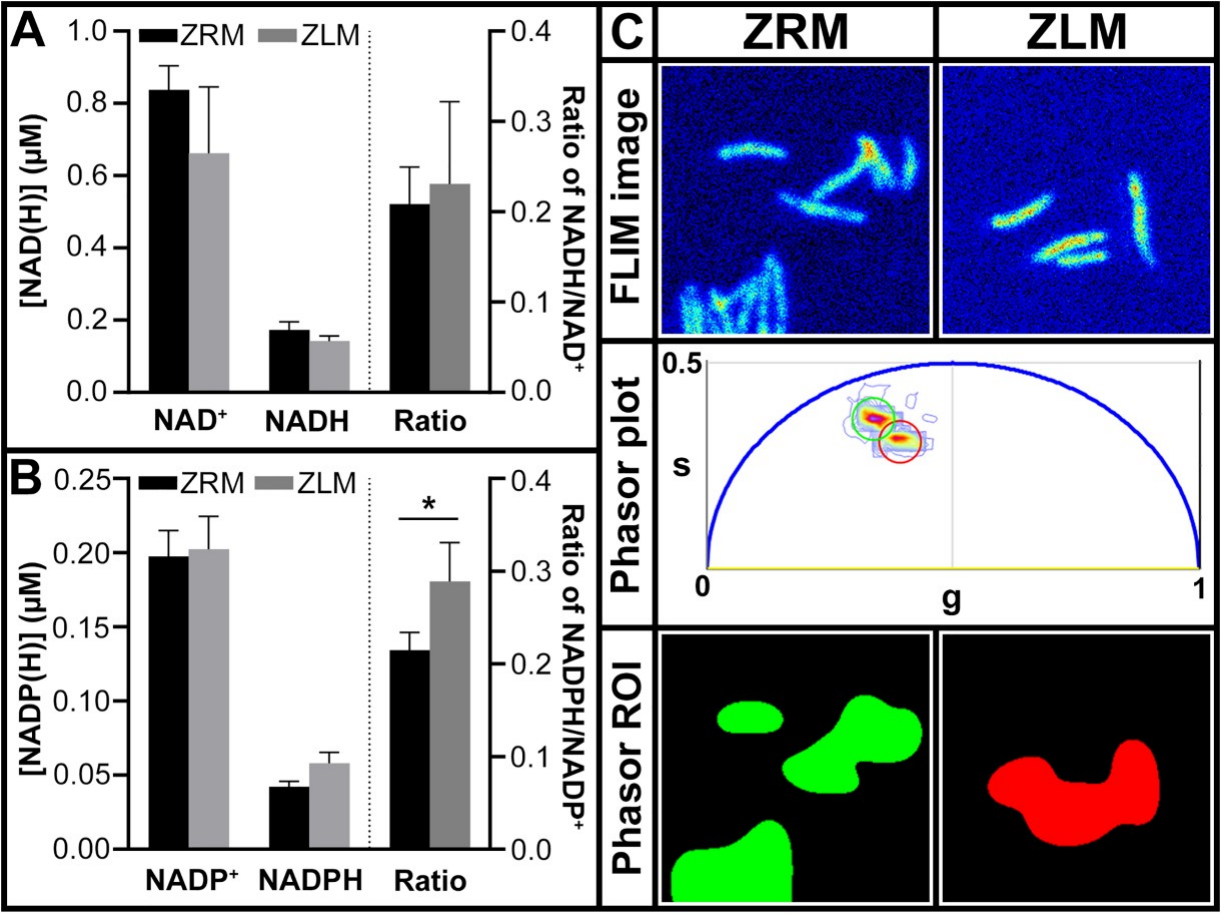
Zn^2+^-limited *Mtb* maintain redox homeostasis and exhibit increased reducing power. Quantification of oxidized (NAD(P)^+^) and reduced (NAD(P)H) nicotinamide adenine dinucleotide cofactors (A) and its phosphorylated forms (B) in *Mtb* mc^2^ 6206 cultures after 10 days of growth in ZLM and ZRM. Data, given as the average of biological replicates (ZRM n = 3, ZLM n = 3) with error bars representing standard deviation, are representative of three independent experiments. Asterisks represent a statistically significant difference (t-test, p-value <0.05) in the ratio of oxidized to reduced forms of nicotinamide cofactors in ZRM and ZLM. (C) Fluorescence lifetime imaging (FLIM) of reduced nicotinamide cofactors (NADH and NADPH) for *Mtb* mc^2^ 6206 cultures after 10 days of growth in ZRM and ZLM. The FLIM micrographs in the top panel are representative fluorescence intensity images for each condition. The phasor plot in the middle shows the phasor positions obtained from the FLIM micrographs where green and red circles encompass the phasor fingerprints of cells grown in ZRM and ZLM respectively. The bottom panels show the region of interest (ROI) encompassed by the encircled areas in the phasor plot projected onto the FLIM micrographs, demonstrating distinct phasor fingerprints between cultures in ZRM and ZLM.

It was previously shown that *Mtb* treated with oxidizing agents had a decreased ratio of NADH/NAD^+^ which was associated with increased sensitivity to oxidation [35,36]. To determine if Zn^2+^-limited *Mtb* experienced altered redox homeostasis, we quantified the amount of oxidized and reduced forms of the nicotinamide cofactors in *Mtb* mc^2^ 6206 cultures grown in ZLM and ZRM. There was no significant difference in the ratio of NADH/NAD^+^ for bacteria grown with or without added Zn^2+^ (Fig 3A), however there was a small, but significant increase in the NADPH/NADP^+^ ratio of Zn^2+^ limited cultures (Fig 3B). Considering the compartmentalized role of these two cofactors in metabolism, these data suggest that the change in redox equilibrium is not generalized, but specifically affecting NADPH-binding enzymes, *i*.*e*., how this factor is utilized.

To further assess utilization of redox cofactors, we visualized the biological activity of NAD(P)H from *Mtb* mc^2^ 6206 in ZLM vs. ZRM using fluorescence lifetime imaging microscopy (FLIM). NAD(P)H FLIM-phasor technique has previously been used to differentiate metabolic states in live bacterial populations at the single-cell level [37]. The phasor positions of cells corresponds to the ratio of free to protein-bound NAD(P)H, and changes in response to environmental factors such as growth phase and history of exposure to antibiotics [37]. Using NAD(P)H FLIM-phasor, we determined that cultures from ZLM and ZRM have distinct phasor fingerprints with Zn^2+^-limited cultures having shorter fractional intensities, indicating a shift towards a freer state for NAD(P)H than that observed in Zn^2+^ replete cultures (Fig 3C). This result indicates that the metabolic state of Zn^2+^-replete and Zn^2+^-limited *Mtb* are distinctly different from one another marked by an increased amount of free reduced nicotinamide cofactors available during Zn^2+^ limitation.

Finally, another coenzyme that has a substantial role in the redox reactions of *Mtb* is cofactor F_420_ [38]. Using fluorescence intensity scans, we observed that oxidized coenzyme F_420_ was detectable in late-log phase cultures (and supernatants) from ZRM while those from ZLM had a loss of peak intensity from the oxidized form of this actinobacterial redox cofactor (S7A and S7B Fig). The loss of peak fluorescence from F_420_ can be quantified by calculating the ratio of fluorescence intensity at excitation wavelengths of 375 nm and 420 nm (Ex_375_/Ex_420_) which offers a simple and robust way to verify the Zn^2+^-limited phenotype (S7C Fig). Although these data do not provide a detailed picture of the mechanisms involved in maintaining redox homeostasis and utilization of the redox cofactors during Zn^2+^ limitation, they indicate that [Zn^2+^] influences the redox signature of *Mtb*.

### Changes in lipidome composition of Zn^2+^-limited *Mtb*

During oxidative stress in *Msm*, in addition to selective upregulation of key detoxifying enzymes (*e*.*g*., catalase and alkylhydroperoxidase) along with the global response to DNA repair, a metabolic switch in lipid metabolism is observed [39]. In our RNAseq and proteomic datasets, several enzymes involved in lipid metabolism were differentially regulated, and “cell wall” was an enriched term in the GO analysis from both genes and proteins in *Mtb* cultures grown in ZLM (S5 Fig and S6 and S7 Tables). Additionally, a clear shift from fatty acid biosynthesis to fatty acid degradation upon Zn^2+^ limitation was observed when looking at DE genes in the context of a global metabolic network (S8 Fig). Together this led us to investigate whether [Zn^2+^] may affect lipidome composition.

**Fig 4:**
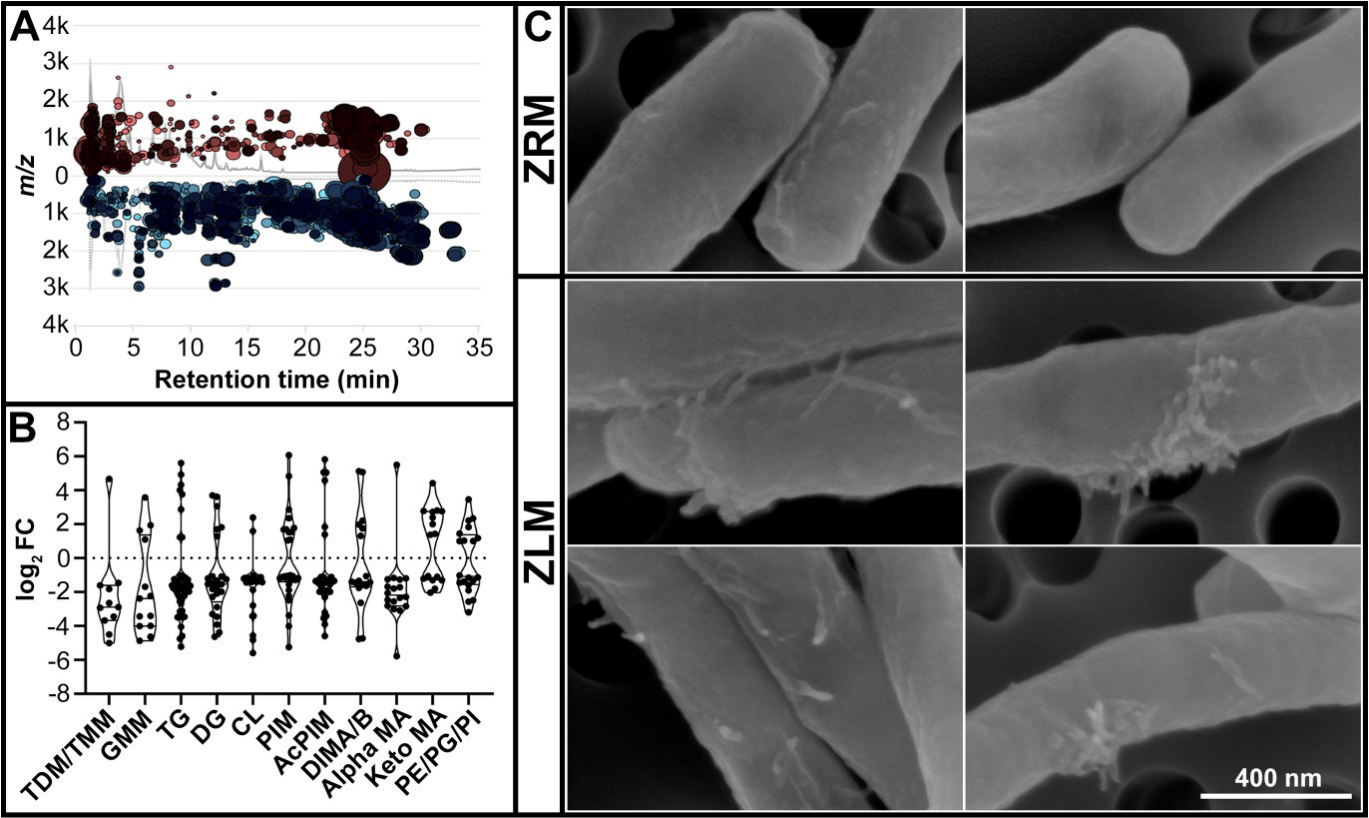
Lipidomic analysis and corresponding surface morphology of *Mtb* in ZLM vs. ZRM. (A) Cloud plot of retention time vs. *m/z* for 7,408 features detected from LC-MS of lipids extracted from *Mtb* H37Rv cells after 10 days of growth in ZRM (n = 3) and ZLM (n = 3). Each bubble on the graph represents a unique feature where red and blue bubbles indicate upregulated and downregulated features respectively in ZLM vs. ZRM. For each bubble, the p-value is represented by opacity (lower p-values appear darker) and the fold-change is represented by the radius (larger fold-changes have larger radii). Only features with p-value < 0.01 and absFC >2 are shown. (B) Violin plot showing log_2_FC for each compound identified from selected lipid classes in ZLM vs. ZRM. The horizontal lines in each violin plot represent the interquartile range. Abbreviations of lipid classes can be found in S1 Text. (C) Scanning electron micrographs representative of *Mtb* mc^2^ 6206 cultures after 10 days of growth in ZRM (n = 3) and ZLM (n = 3). All images are taken at 120kX magnification, the scale bar applies to all images.

We analyzed the lipidomes of *Mtb* H37Rv grown in ZLM and ZRM using LC-MS. Lipid analysis showed remodeling of the lipidome during Zn^2+^ limitation. Many features had decreased relative abundance in Zn^2+^-limited condition as seen by the number of compounds downregulated on the cloud plot in Fig 4A. From the precursor ions detected, 307 features were identified as mycobacterial lipids, with 229 of these lipids having decreased relative abundance and 77 having increased relative abundance in ZLM vs. ZRM (S8 Table). In *Mtb*, *de novo* synthesis of short-chain fatty acids from acetyl-CoA initiates with the eukaryotic-like fatty acid synthase I (*fas*) [40]. *Fas* gene was significantly downregulated in ZLM (S4 Table) and this enzyme is responsible for many of the reactions involved in fatty acid biosynthesis that are downregulated in ZLM (S8 Fig). Additionally, 4/5 genes in the *fasII* operon (*acpM*, *kasA*, *kasB* and *accD6*), responsible for chain elongation in the biosynthesis of meroacids [40], were also downregulated in ZLM (S4 Table), with AccD6 being downregulated at both the RNA and protein level (Table 1).

Mycolic acids are an integral component of the mycobacterial cell wall and are required for virulence of *Mtb* [40]. Many forms of mycolic acids had decreased relative abundance in the Zn^2+^-limited condition, specifically mycolate moieties esterified to carbohydrates in the outer leaflet of the mycomembrane (*i*.*e*., trehalose monomycolate -TMM and trehalose dimycolate -TDM) (Fig 4B and S8 Table). Full-length, matured mycolic acids are transferred across the lipid bilayer to be incorporated into the cell wall by inner membrane transporters, upon which antigen 85 complex transfers mycolyl groups from TMM onto cell-wall arabinogalactan forming arabinogalactan-mycolate or onto other TMM moieties forming TDM [41]. In agreement with the decreased relative abundance of TMM and TDM in ZLM, the gene encoding the secreted antigen 85C protein (*fbpC*) was strongly downregulated in ZLM (S4 Table). In addition, Zn^2+^-limited *Mtb* showed a marked decrease in relative abundance of triacyl glycerides (TG) (Fig 4B), a class of lipids stored as energy reserves during stress conditions [42], consistent with the downregulation of TG synthase enzymes *tgs1* and *tgs2* (S4 Table). Overall, the results show that [Zn^2+^] affects the lipidome of *Mtb* with Zn^2+^-limited bacilli exhibiting decreased relative abundance of specific lipid classes, including triacyl glycerides and mycolic acids.

Next, we investigated whether the global changes observed in the lipidome were reflected in surface features of *Mtb* mc^2^ 6206 grown in ZRM vs. ZLM. Using scanning electron microscopy (SEM), we observed two features of cells grown in ZLM that differentiated them from those grown in ZRM; the cells were more clumpy (even after enrichment for single cells) (S9 Fig) and most remarkably, there were many finger-like protrusions (fibrils) extending from cells in ZLM (Fig 4C). The protrusions from the cell walls of ZLM cultures observed with SEM are very similar in appearance to those features observed in *Mtb* mutants deficient in *kasA*, a key enzyme of mycolic acid biosynthesis [43] that is among the *fasII* operon enzymes downregulated in ZLM. Therefore, morphological changes observed on the surface of *Mtb* grown under Zn^2+^ limited conditions may be caused by differential expression of some key enzymes involved in lipid biosynthesis.

### Zn^2+^-limited *Mtb* exhibit increased resistance to oxidative stress *in vitro* and cause higher bacterial burden and pathology *in vivo*

As presented above, an expected consequence of Zn^2+^ limitation is exposure to ROS which is supported by increased antioxidant (*e*.*g*., KatG and AhpC) expression in ZLM (Table 1). Considering Zn^2+^-limited bacteria exhibit many features of cells exposed to elevated levels of ROS that correlate with pathogen virulence [44,45], we investigated whether Zn^2+^ limitation contributes to altered susceptibility of this subpopulation to specific antibiotics, exogenous oxidative stress, and/or affects virulence.

**Fig 5:**
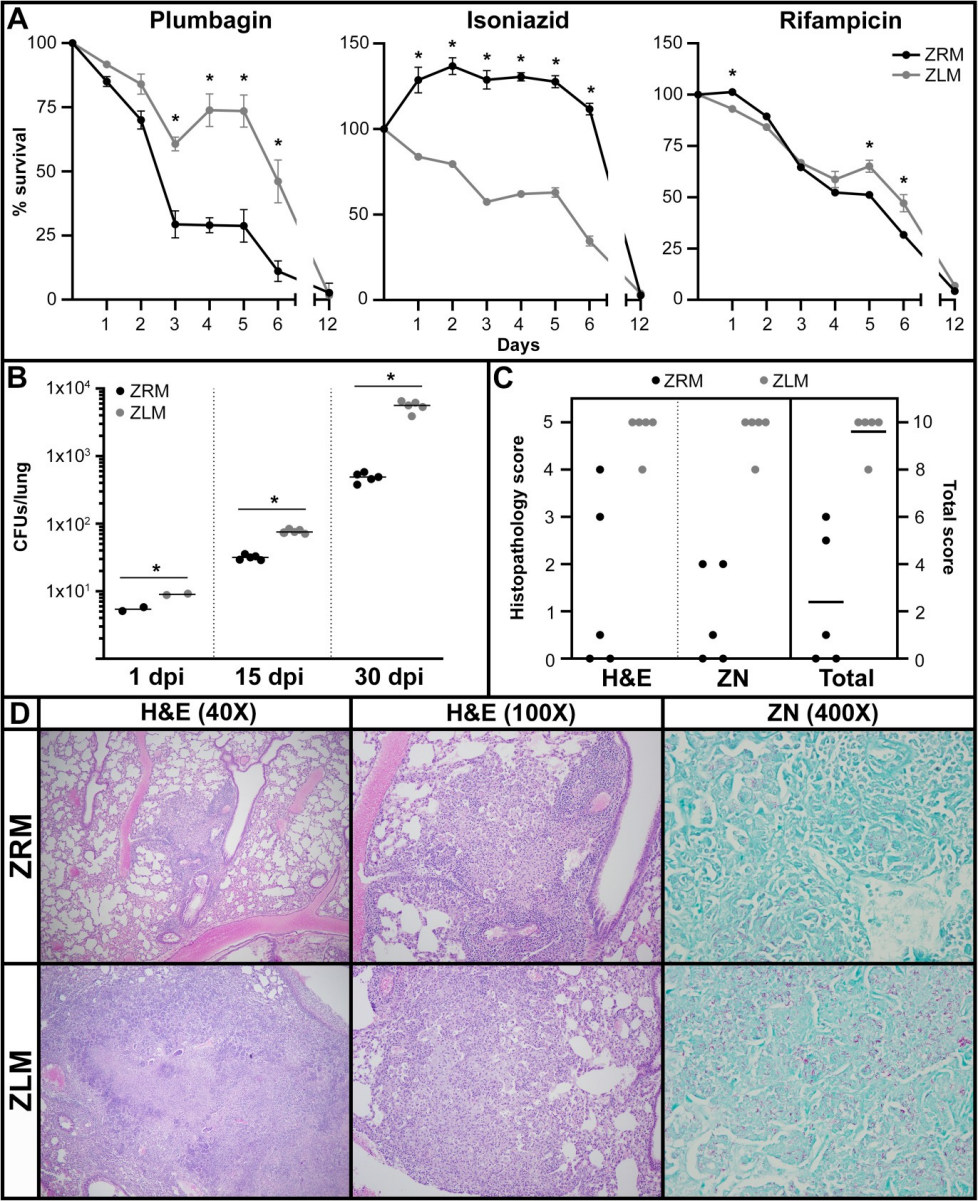
Zn^2+^-limited *Mtb* exhibit increased resistance to oxidative stress *in vitro* and cause higher bacterial burden and pathology *in vivo*. (A) Survival of *Mtb* mc^2^ 6206 after growth in ZRM or ZLM and subsequent exposure to the indicated chemical or antibiotic. Survival was monitored with flow cytometry for twelve days following treatment and was calculated as the percentage of live cells in untreated cultures at the beginning of treatment (S10 Fig). The data are representative of three independent experiments and are given as the average of biological replicates (ZRM n = 3, ZLM n = 2) with error bars representing standard deviation. Asterisks represent a statistically significant difference (t-test, p-value <0.05) between survival of cultures in ZRM vs. ZLM at any given time-point. (B) Bacterial burden in the lungs of C3HeB/FeJ (Kramnik) mice after 1, 15 and 30 days post-infection (dpi) with *Mtb* H37Rv pre-grown in ZRM or ZLM. Horizontal bars across the data points represent the average CFUs. Asterisks represent a statistically significant difference (t-test, p-value <0.05) between CFUs of mice infected with ZRM vs. ZLM at each timepoint. (C) Blinded histopathology scores from hematoxylin and eosin (H&E) and Ziehl-Neelsen (ZN) staining in panel D. Maximal score of five for H&E and ZN stained cross sections is based on observed changes in lung morphology and immune cell infiltration and bacterial burden respectively and these scores are plotted on the left y-axis. The total score is the sum of the H&E and ZN score for each lung and is given on the right y-axis with a score of 10 representing the maximal level of disease pathology. The horizontal bar through the data for total score represents the average total pathology score. The non-parametric Mann-Whitney U-test for significance was applied to histopathology scores from H&E, ZN and total, all resulting p-values <0.02 indicating a significant difference in pathology scores for mice infected with *Mtb* pre-grown in ZRM or ZLM. (D) Histopathology micrographs of the lungs of mice infected with *Mtb* H37Rv pre-grown in ZRM or ZLM at 30 days post-infection. In blinded studies, histology cross-sections of lung tissues were stained with H&E to score changes in lung morphology and immune cell infiltration or ZN stain for mycobacteria (pink) to assess bacterial burden.

To test the effect of [Zn^2+^] on antibiotic susceptibility, we used a flow cytometry method to analyze *Mtb* mc^2^ 6206 grown in ZRM or ZLM and treated with several common antibiotics and oxidizing agents (S10 Fig). This method captures information about viable bacteria not immediately culturable under standard conditions after antibiotic exposure [46]. Using flow cytometry, we also observed increased clumping as seen with SEM, Zn^2+^-limited cultures (even after enrichment for single cells) had increased abundance of particles at higher forward scatter intensities indicating larger particle sizes (clumps) in ZLM condition (S7 Fig). Results demonstrated that, consistent with upregulation of antioxidant and enzymes involved in DNA repair in ZLM, cells grown in this condition exhibited increased viability when exposed plumbagin, an oxidizing and genotoxic agent (Fig 5A). A similar trend was observed for rifampicin, an antibiotic known to generate ROS in *Mtb* [47]. A difference in killing with rifampicin was more prominent at the later stages, consistent with the observation that sterilizing activity of rifampicin is time dependent [46] (Fig 5A). On the other hand, cultures from ZLM experienced significantly more rapid killing when treated with isoniazid, consistent with the upregulation of KatG (confirmed in mc^2^ 6206 strain with western blot, S11 Fig), the isoniazid-activating enzyme in *Mtb* [48] (Fig 5A). No difference in killing was observed for ethambutol or kanamycin, indicating that differences in antibiotic sensitivities are specific (S12 Fig). In conclusion, the Zn^2+^-limited population exhibited differential response to certain treatments compared to the Zn^2+^ replete population.

In addition to altered survival with exposure to antibiotics, differential expression of key enzymes upregulated in ZLM has been correlated with increased survival and virulence *in vivo* [44,45]. Considering that certain changes in the transcriptome and proteome of Zn^2+^-limited *Mtb* affect virulence, along with the finding that *Mtb* transits through a Zn^2+^ limited environment during infection and is likely primed by low [Zn^2+^] before being transmitted to naïve hosts, it is relevant to determine if [Zn^2+^] alone can affect virulence of *Mtb*. To test this, we used C3HeB/FeJ (Kramnik) mice which develop liquefied necrotic granulomas resembling human disease pathology [49]. Mice were infected by aerosol delivery of *Mtb* pre-grown in ZLM or ZRM (S13 Fig), producing comparable, albeit significantly different (1.7-fold ZLM vs. ZRM, p-value = 0.01265) bacterial numbers in the lungs at 1 day post-infection (dpi) (Fig 5B). Interestingly, bacteria preconditioned by prolonged growth in Zn^2+^-limiting conditions exhibited significantly higher bacterial burden in the lungs by 15 (2.4-fold, p-value <0.000001) and 30 (11.3-fold, p-value = 0.000004) dpi. (Fig 5B). Accordingly, throughout the infection mice infected with *Mtb* pre-grown in ZLM provoked increased pulmonary granulomas, with more observable bacteria, considerable tissue destruction, and increased neutrophilic infiltration in the lungs (Figs 5C, 5D and S14A). Although pathology was absent or minimal in livers and spleens (S14 and S15 Figs), by 30 dpi there was a significant increase in the bacterial burden in these organs when mice were infected with inoculum from ZLM vs. ZRM (S15A Fig). These data indicate that exposure to different [Zn^2+^] may contribute to virulence of *Mtb*, considering inoculum from ZLM had increased ability to replicate and cause tissue damage in the lungs.

### Adaptive response to prolonged Zn^2+^limitation in *Mtb*

We have demonstrated that [Zn^2+^] influences a myriad of changes at the gene level correlating with changes at the protein level and translating into altered physiological characteristics of [Zn^2+^]-derived populations of *Mtb*. Many Zn^2+^ binding proteins have essential biological functions [50], but we found no evidence of a generalized decrease in expression/abundance of these proteins in the response to Zn^2+^ limitation (S9 Table). This observation indicates that *Mtb* grown in ZLM were not severely limited for this nutrient, which is also evident from typical growth curves (S2C and S2D Fig). Nonetheless, the response to prolonged Zn^2+^-limitation in *Mtb* is robust and complex given the vast global adaptations employed by bacteria grown in ZLM, most of which are not directly related to Zn^2+^ conservation. In addition to the changes described in the sections above, there were many other genes and pathways affected by [Zn^2+^] whose significance is unknown and beyond the scope of this paper but support the existence of an adaptive response during Zn^2+^-limitation. These changes include: 1.) downregulation of many well described antigens in ZLM (*e*.*g*., *fbpC* {Ag85-C}, *ald* {Ald, 40 Kd-Ag, TB43}, TB31.7, 35 Kd-Ag, *hspX* {16 Kd-Ag, α-crystallin}, *esxA* {ESAT-6}) (Table 1 and S4 and S5 Tables) consistent with decreased expression of ABC transporters (S6B Fig and S4 Table), 2.) decreased electron transport, oxidative phosphorylation and ATP production (S6B and S8 Figs) as indicated by decreased cytochrome *bc* (*qcrCAB*) and *bd* (*cydB-D*) oxidases, NADH-dehydrogenase I (*nuoE-N*) and ATP-synthase (*atpC-E*) (S4 Table), and 3.) a significant decrease in genes involved in the hypoxic response (39/49 genes the *Mtb* H37Rv DosR regulon [51]) (Fig 2B and S10 Table).

In addition to Zur-regulated genes (S3 Table) and the numerous processes involved in DNA replication, mismatch repair and recombination (Figs 2B and S6A and S6 Table) other genes upregulated in ZLM included many PE/PPE proteins, genes involved in biosynthesis pathways and metabolism of diverse substrates (*e*.*g*., amino acids) (S6 Table) and isocitrate lyase (Icl) was significantly upregulated at the gene and protein level (Table 1). Another consequence of Zn^2+^ limitation was altered Cu^2+^ homeostasis; specifically, there was a strong induction of the copper-sensitive operon repressor CsoR and copper export transporter CtpV (Table 1). Zn^2+^ limitation altered the expression of numerous genes involved in transcriptional regulation; *whiB6* and *sigG* expression were increased while *sigE*, *sigD*, and ten genes involved in two-component signal transduction systems (*e*.*g*., *devS*/*devR*, *mprA*/*mprB* and *narG*/*narI*/*narX*) were decreased in ZLM (S4 and S7 Tables). Altogether, Zn^2+^ limitation contributes to phenotypic heterogeneity, causing dramatic changes in expression patterns of many genes and pathways that directly influence physiology of *Mtb*.

## Discussion

**Fig 6:**
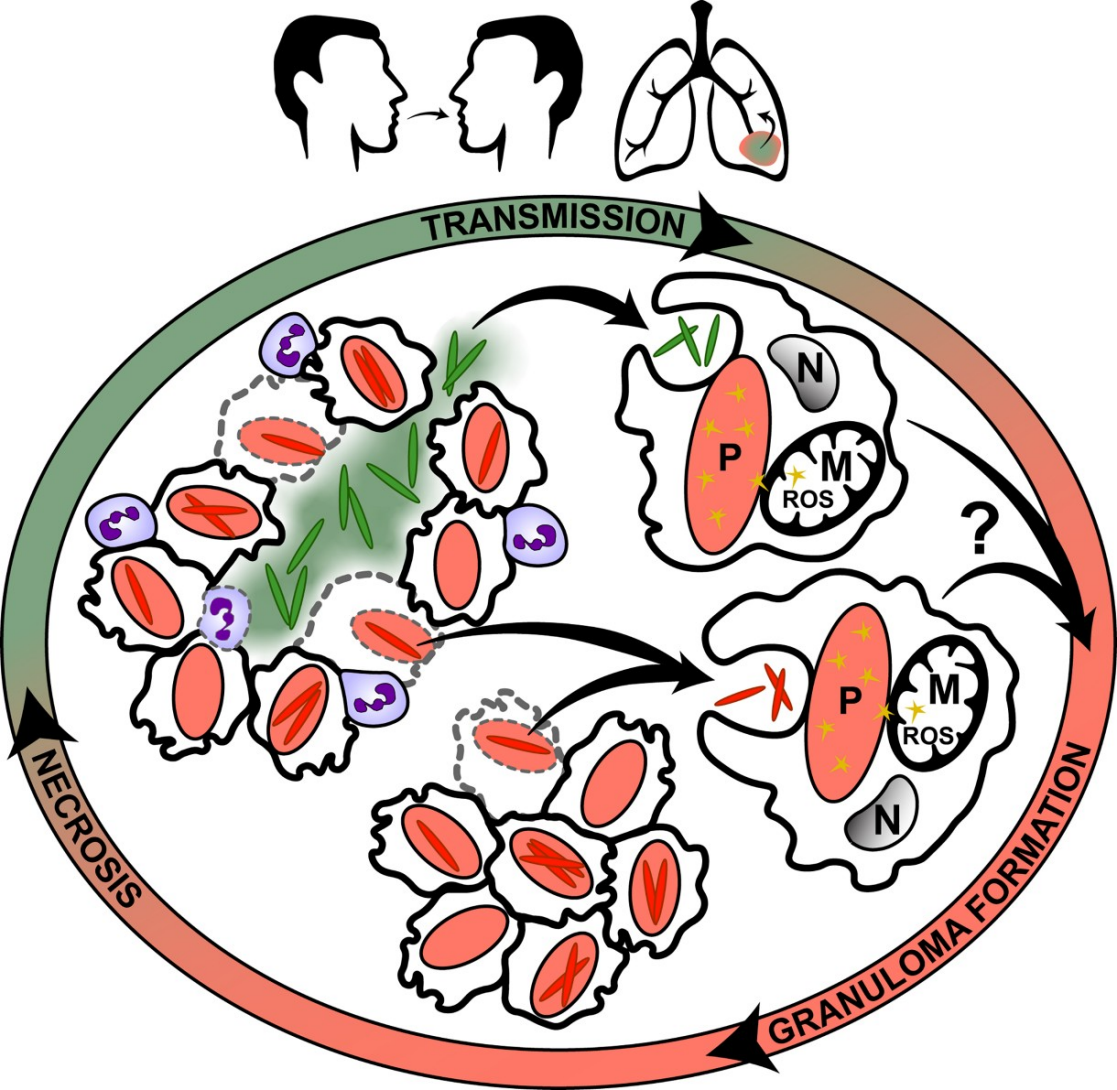
Changes in [Zn^2+^] throughout TB infection cycle drives formation of Zn^2+^-limited *Mtb* with anticipatory adaptations. The gradient around the outside of the figure represents the cycle of changing [Zn^2+^] throughout infection; red indicates Zn^2+^-replete and green indicates Zn^2+^-limited microenvironments. These [Zn^2+^]-defined microenvironments drive formation of physiologically distinct subpopulations of *Mtb* with Zn^2+^-replete *Mtb* (*red rods*) in phagosomes and Zn^2+^-limited *Mtb* (*green rods*) exposed to CP, *e*.*g*., in the caseum. After phagocytosis of *Mtb*, solid granulomas form (*bottom*) and sustained inflammation leads to the recruitment of neutrophils (*purple*) which cause necrosis (*left*). *Mtb* may be transmitted from necrotic or apoptotic cells (*grey dashed margins*) in either solid or necrotic granulomas. With the latter, *Mtb* transit through the Zn^2+^-limited caseum before being transmitted host-to-host or within a single infected individual (*top*). The Zn^2+^-limited *Mtb* subpopulation has adaptations that could enable this subpopulation to anticipate forthcoming stress and resist host killing (*e*.*g*., oxidative stress–*yellow stars*) and/or affect immune cell activation. The exact mechanisms of how [Zn^2+^]-derived changes in physiology of *Mtb* may affect disease outcome are unclear and we highlight the importance of defining the host response to Zn^2+^-replete and Zn^2+^-limited *Mtb* (*question mark*). Drawings are representative and not to scale and some components of granulomas have been omitted for clarity. Abbreviations: nucleus (N), mitochondria (M), phagosome (P).

Numerous pathogens experience drastic changes in free [Zn^2+^] during infection and have adapted elaborate mechanisms to endure Zn^2+^ toxicity and Zn^2+^ limitation *in vivo* [52,53]. Despite the relevance of the Zn^2+^-depleted niche in the lifecycle of *Mtb*, the response to prolonged Zn^2+^ limitation remained undefined in this pathogen. In this study, we suggest that [Zn^2+^] is likely a physiologically relevant signal experienced by *Mtb in vivo*, since we were able to demonstrate that this micronutrient triggers the formation of distinct populations *in vitro*. Zn^2+^-limited *Mtb* exhibit a global adaptive response that affects physiology, confers resiliency to oxidative stress and possibly leads to increased virulence. *Mtb* depends on a cycle of exit and re-entry into host immune cells to perpetuate its lifecycle, and [Zn^2+^] may be a major cue experienced in this cycle that could potentially affect host-pathogen interactions and disease outcome (Fig 6).

After establishing *in vitro* conditions that induce Zur regulon, we used a multi-omics approach to compare Zn^2+^-replete and Zn^2+^-limited *Mtb*. We detected upregulation of genes in the Zur regulon including those involved in Zn^2+^ acquisition and replacing Zn^2+^-dependent ribosomal proteins, as expected [11,12,54]. Interestingly, the ESX-3 gene cluster had the lowest expression levels among Zur-regulated genes, however this finding is explained by the dual regulation of this gene cluster by both Zn^2+^ (*i*.*e*., Zur) [11] and iron (*i*.*e*., IdeR) [55], which is not limited in ZLM. The effect of copper (Cu^2+^) was the opposite, Zn^2+^-limited *Mtb* appear to reduce Cu^2+^ uptake as seen in other Zn^2+^-limited bacteria [15]. Importantly, the oxidative stress response was a marked feature of Zn^2+^-limited *Mtb* including significantly increased expression of antioxidant enzymes KatG and AhpC and genes involved in DNA replication, mismatch repair and recombination, along with accumulation of reducing cofactor NADPH. While there is a precedence for increased antioxidant production and mechanisms to conserve NADPH in Zn^2+^-limited yeast [31,33], similar protective mechanisms have not been discovered for Zn^2+^-limited bacteria until now.

In agreement with our observations and consistent with the notion that Zn^2+^ limitation associates with oxidative stress, multiple different mechanisms leading to elevated oxidative stress in *Mtb* also modulate expression of antioxidants and enzymes involved in lipid biosynthesis and DNA repair [34,56,57]. Increased expression of *sigG* in ZLM, the alternative sigma factor upregulated by DNA damaging agents [58], further confirms the response to DNA damage during Zn^2+^ limitation. Beyond increased capacity to repair damaged DNA, Zn^2+^ limitation affected other physiological responses that have roles in antioxidant defense or are induced upon oxidative stress, including increased expression of genes involved with iron storage (*bfrB*) and regulation (*furA*) [56,59], isocitrate lyase (*icl1*) [56,60], PE and PPE genes [34], transfer RNAs [61], thioredoxin reductase (*trxB2*) [62] and WhiB6 gene [45,63]. In this study, Zn^2+^-limitation is implicated for the first time in induction of the oxidative stress response in *Mtb*, an adaptive response highly similar to Zn^2+^-limited pathogenic fungi [64]. The combined effect of an upregulated oxidative stress response, increased reducing power (NADPH), and processes involved in DNA repair likely affords *Mtb* the ability to adapt to diverse host environments in the context of [Zn^2+^].

There is a precedence that mycobacteria primed by exposure to oxidative stress have enhanced survival in the host [65,66]. This is, in part, due to two well described virulence factors, catalase (KatG) and alkylhydroperoxide reductase (AhpC), critical elements of the antioxidant defense system, whose expression is induced by oxidative stress and correlates with enhanced survival and hypervirulence in animal models [34,44,45,67]. Owing to inactivation of the oxidation-sensing regulator OxyR in the genome of *Mtb*, this pathogenic mycobacterium is assumed to have evolved altered regulation of the oxidative stress response [68] and *ahpC* expression is thought to be silenced under aerobic conditions [69]. Here, we provide evidence that *ahpC* is not always silenced under aerobic conditions—it is induced upon Zn^2+^ limitation even in an aerobic environment, a significant finding considering de-repression of *ahpC* is a mechanism believed to enable survival of bacteria specifically during the transition from latency to active infection [70]. AhpC expression in bacilli exposed to Zn^2+^ limited environments provides a possible mechanism for sustained survival in the host. Therefore, our results suggest that oxidative stress associated with Zn^2+^ limitation is a previously unappreciated signal modulating the expression of virulence factors in *Mtb*.

Hypoxia, not [Zn^2+^], is the best characterized and appreciated property of the tubercle necrotic granuloma [71]. However, a spectrum of granuloma types exists within a single infected individual [3] and while hypoxia is strongly associated with deeper regions of necrotic granulomas [71], CP-expressing neutrophils are present in both necrotic and non-necrotic granulomas [10]. It is interesting that transcriptomic and phenotypic analysis of *Mtb* from sputum were found to have a decrease in enzymes involved in PDIM synthesis, protein export systems and a low energy state (decreased ATP synthase genes) [13,14,72], just as we have described to occur upon Zn^2+^ limitation. The two major differences between transcripts and phenotypes from Zn^2+^-limited *Mtb* and those from sputum were decreased accumulation of TGs and expression of genes involved in the hypoxic response (*e*.*g*., DosR regulon) in Zn^2+^-limited condition, another feature shared with Zn^2+^-limited pathogenic fungi [64]. One possible explanation for this observation is due to decreased oxidative phosphorylation, and presumably a concurrent decrease in oxygen consumption during Zn^2+^ limitation, which may be an anticipatory response signaled by low [Zn^2+^] in preparation for transit through the hypoxic caseum. The complete lack of DosR-mediated hypoxic response in Zn^2+^-limited *Mtb* demonstrates that [Zn^2+^] alone does not recapitulate the microenvironment of sputum or the necrotic granuloma. We suggest that, while hypoxia is a relevant cue to bacilli in certain microenvironments during tuberculosis, [Zn^2+^] may be another cue that delineates bacterial physiology *in vivo*, possibly even independent of hypoxia, and we emphasize that the combined effects of hypoxia and Zn^2+^ limitation remains obscure.

[Zn^2+^] alone dramatically contributes to changes in mycobacterial physiology. We discovered that Zn^2+^-limited bacteria have increased resistance to exogenous oxidation as predicted by increased antioxidant expression in this condition. While Zn^2+^-limitation did not have a broad effect on antibiotic susceptibility, Zn^2+^-limited bacteria were more resistant to ROS-producing rifampicin while increased KatG expression sensitized this subpopulation to the prodrug isoniazid [73]. In addition, changes in the cell wall and other lipid classes in Zn^2+^-limiting condition can be attributed to a global downregulation in fatty acid biosynthesis at both the gene (*fas* and *fasII* operon) and lipidome level, which may be associated with the increased abundance of free NADPH observed during Zn^2+^ limitation considering NADPH is an essential cofactor for fatty acid biosynthesis. Decreased mycolic acid content of the Zn^2+^-limited mycomembrane resulted in an observable phenotype marked by finger-like protrusions (fibrils) extending from the surface of cells in ZLM. Cell wall remodeling is utilized by *Mtb* to interact and persist within the host [74] and the Zn^2+^-dependent changes in the cell wall may contribute to altered virulence of Zn^2+^-limited *Mtb*. Moreover, Zn^2+^-limited cells showed increased clumping behavior, another cell surface related phenotype that also may play a role in pathogenesis. It has been shown that once a clump of *Mtb* initiates death of a single macrophage it can lead to serial killing of other macrophages and loss of control over the infection, and it was highlighted that an important next step will be to show how the original clump of *Mtb* is formed [75]. All in all, Zn^2+^ limitation drives phenotypic heterogeneity in *Mtb in vitro* and may be a signal used by *Mtb* to anticipate forthcoming stress and alter its physiology in ways to promote survival and dissemination.

Classically described as an intracellular parasite, *Mtb* invades and replicates within host macrophages, and extracellular *Mtb* in the necrotic milieu face one of two fates for survival; expulsion from the lungs through aerosols (a required step for disease transmission), or re-phagocytosis from competent immune cells at the site of the active lesion. Either way, *Mtb* must re-enter host immune cells to perpetuate its life-cycle, and, upon phagocytosis, the infected macrophage initiates a respiratory burst producing high levels of ROS to kill the pathogen [76] (Fig 6). However, *Mtb* possesses resistance mechanisms to evade or counter the phagocytic respiratory burst (*e*.*g*., KatG), thus masking the impact of this antimicrobial defense [77]. The fact that Zn^2+^ limitation primes individual bacilli with the resistance mechanisms needed to evade ROS-derived killing and persist in the host suggests that transit through the Zn^2+^ limited environment could aid bacterial survival *in vivo*. Indeed, we show that Zn^2+^-limited *Mtb* manifest more severe disease in mice, marked by increased bacterial burden and disease pathology. It is unclear whether the modest difference in bacterial numbers at 1 dpi was due to more efficient initial inoculation that might not reflect altered virulence *per se*, however this initial difference does not fully explain the large difference in bacterial numbers and disease pathology from Zn^2+^-limited inoculum observed at later time points. The heightened disease outcome in response to Zn^2+^-limited inoculum may also be due to increased evasion of killing upon initial contact with macrophages and/or differential priming of the immune system, given the many changes to the lipidome, surface morphology and antigens produced in Zn^2+^-limited bacteria, their clumping behavior or other unknown mechanisms (Fig 6). Albeit just one study in one mouse strain, our findings suggest that Zn^2+^-dependent phenotypic heterogeneity in *Mtb* may have a significant effect on disease progression. We speculate that Zn^2+^ limitation triggers an anticipatory response against impending phagocytic attack to promote host colonization, as seen in pathogenic fungi exposed to iron limitation [78]. Anticipatory metabolic mechanisms have been described in *Mtb* [79] and the capacity for adaptive prediction of environmental changes in microorganisms has been shown [80]. Although further *in vivo* studies are warranted to provide more evidence for the role of [Zn^2+^] in TB pathogenesis in humans and animal models, our *in vitro* data suggest that Zn^2+^ limitation may cue an anticipatory response in extracellular *Mtb*, stimulating the bacillus to employ protective mechanisms in preparation for the imminent phagocytosis.

Modes of phenotypic heterogeneity in *Mtb* have been widely recognized as driving forces acting upon individual bacilli which can significantly impact host-pathogen interactions and treatment outcomes [81]. In this study, we demonstrate significant changes in the transcriptome, proteome and lipidome of *Mtb* depending on [Zn^2+^], including upregulation of numerous transcription factors and enzymes involved in the oxidative stress response leading to a global adaptive response during Zn^2+^ limitation. Accordingly, predisposition of individual *Mtb* bacilli to the Zn^2+^-limited microenvironment could prime them to interact differentially with the host during infection (Fig 6). Here, for the first time, we suggest that [Zn^2+^] itself may be a driving factor in the development of phenotypic heterogeneity in *Mtb in vivo*, and subpopulations of *Mtb* developing in the Zn^2+^-depleted niche of necrotic granulomas may affect containment and spread of the bacillus both within the host and the human population.

## Materials and methods

### Ethics statement

Granulomas from cynomolgus macaques were obtained from animals that were enrolled in completed studies and all work had been previously approved by the University of Pittsburgh’s Institutional Animal Care and Use Committee. All experimental animals (mice) used in this study were approved by the Texas A&M University Institutional Animal Care and Use Committee. Sputum leftovers from TB testing were obtained without identifiers from Hawaii Department of Health and were not considered human subject study. Detailed materials and methods are provided in S1 Text.

## Supporting information

S1 Fig Validation of rabbit polyclonal antibodies raised against *Mtb* ribosomal proteins S18-1 and S18-2. (PDF)

S2 Fig Growth and morphology of *Mtb* H37Rv and mc^2^ 6206 in ZLM and ZRM. (PDF)

S3 Fig Multi-dimensional scaling (MDS) plots for *Mtb* H37Rv transcriptomics (A) and proteomics (B). (PDF)

S4 Fig Heatmap of tRNA molecules detected in RNAseq of *Mtb* H37Rv. (PDF)

S5 Fig Gene ontology enrichment analysis of differentially expressed genes (A) and proteins (B) from *Mtb* H37Rv. (PDF)

S6 Fig Pie charts showing KEGG pathways of DE genes in ZLM vs. ZRM. (PDF)

S7 Fig Loss of peak fluorescence from redox cofactor F_420_ under Zn^2+^-limiting conditions and the association of F_420_ loss with Zn^2+^-limited phenotypes. (PDF)

S8 Fig Genes belonging to KEGG metabolic pathways that are upregulated (red) or downregulated (blue) in ZLM vs. ZRM and superimposed onto a global metabolic network. (PDF)

S9 Fig Clumping of cells in ZLM observed with scanning electron microscopy (SEM). (PDF)

S10 Fig Controls for flow cytometry killing experiment showing gating strategy for bacteria based on forward vs. side scatter and bacteria stained with the live cell stain Calcein-AM (C-AM) and the dead cell stain SYTOX. (PDF)

S11 Fig Detection of KatG protein in lysates from *Mtb* mc^2^ 6206 in ZRM and ZLM. (PDF)

S12 Fig Survival of *Mtb* mc^2^ 6206 after growth in ZRM (black) or ZLM (grey) and subsequent exposure to the indicated antibiotics. (PDF)

S13 Fig Schematic of mouse infection experiment with *Mtb* H37Rv pre-grown in ZRM or ZLM. (PDF)

S14 Fig Histopathology scores from the lungs (A), livers (B), and spleens (C) of mice after 15 days of infection with *Mtb* H37Rv pre-grown in ZRM or ZLM. (PDF)

S15 Fig Bacterial burden and histopathology scores from the livers and spleens of mice after 30 days of infection with *Mtb* H37Rv pre-grown in ZRM or ZLM. (PDF)

S1 Table Concentration of CP in sputum samples from TB patients. (XLSX)

S2 Table Differentially expressed genes in TPEN vs. CP treatment. (XLSX)

S3 Table Differentially expressed genes and proteins from *Mtb* Zur regulon in ZLM vs. ZRM. (XLSX)

S4 Table Differentially expressed genes in ZLM vs. ZRM. (XLSX)

S5 Table Differentially expressed proteins in ZLM vs. ZRM. (XLSX)

S6 Table Assignment of DE genes upregulated in ZLM to KEGG pathways. (XLSX)

S7 Table Assignment of DE genes downregulated in ZLM to KEGG pathways. (XLSX)

S8 Table Features detected from LC-MS analysis and identified as *Mtb* lipids by cross-referencing with *Mtb* LipidDB. (XLSX)

S9 Table Expression values of Zn^2+^-binding proteins at the gene and protein level. (XLSX)

S10 Table Expression values of genes and proteins in the DosR regulon in ZLM vs. ZRM. (XLSX)

S1 Data Supporting Data. (XLSX)

S1 Text Materials and Methods. (PDF)

## References

[1] Philips JA, Ernst JD. Tuberculosis Pathogenesis and Immunity. Annu Rev Pathol Mech Dis. 2012;7: 353–384. 10.1146/annurev-pathol-011811-132458 22054143

[2] Barry CE, Boshoff HI, Dartois V, Dick T, Ehrt S, Flynn J, et al. The spectrum of latent tuberculosis: rethinking the biology and intervention strategies. Nat Rev Microbiol. 2009;7: 845–855. 10.1038/nrmicro2236 19855401 PMC4144869

[3] Lenaerts A, Barry CE, Dartois V. Heterogeneity in tuberculosis pathology, microenvironments and therapeutic responses. Immunol Rev. 2015;264: 288–307. 10.1111/imr.12252 25703567 PMC4368385

[4] Hoff DR, Ryan GJ, Driver ER, Ssemakulu CC, De Groote MA, Basaraba RJ, et al. Location of Intra- and Extracellular M. tuberculosis Populations in Lungs of Mice and Guinea Pigs during Disease Progression and after Drug Treatment. Tailleux L, editor. PLoS One. 2011;6: e17550. 10.1371/journal.pone.0017550 21445321 PMC3061964

[5] Cadena AM, Fortune SM, Flynn JL. Heterogeneity in tuberculosis. Nat Rev Immunol. 2017;17: 691–702. 10.1038/nri.2017.69 28736436 PMC6247113

[6] Zackular JP, Chazin WJ, Skaar EP. Nutritional Immunity: S100 Proteins at the Host-Pathogen Interface. J Biol Chem. 2015;290: 18991–18998. 10.1074/jbc.R115.645085 26055713 PMC4521021

[7] Wagner D, Maser J, Lai B, Cai Z, Barry CE, Höner zu Bentrup K, et al. Elemental Analysis of Mycobacterium avium -, Mycobacterium tuberculosis -, and Mycobacterium smegmatis -Containing Phagosomes Indicates Pathogen-Induced Microenvironments within the Host Cell’s Endosomal System. J Immunol. 2005;174: 1491–1500. 10.4049/jimmunol.174.3.1491 15661908

[8] Botella H, Peyron P, Levillain F, Poincloux R, Poquet Y, Brandli I, et al. Mycobacterial p(1)-type ATPases mediate resistance to zinc poisoning in human macrophages. Cell Host Microbe. 2011;10: 248–59. 10.1016/j.chom.2011.08.006 21925112 PMC3221041

[9] Ramakrishnan L. Revisiting the role of the granuloma in tuberculosis. Nat Rev Immunol. 2012;12: 352–366. 10.1038/nri3211 22517424

[10] Mattila JT, Ojo OO, Kepka-Lenhart D, Marino S, Kim JH, Eum SY, et al. Microenvironments in Tuberculous Granulomas Are Delineated by Distinct Populations of Macrophage Subsets and Expression of Nitric Oxide Synthase and Arginase Isoforms. J Immunol. 2013;191: 773–784. 10.4049/jimmunol.1300113 23749634 PMC3746594

[11] Maciąg A, Dainese E, Rodriguez GM, Milano A, Provvedi R, Pasca MR, et al. Global Analysis of the Mycobacterium tuberculosis Zur (FurB) Regulon. J Bacteriol. 2007;189: 730–740. 10.1128/JB.01190-06 17098899 PMC1797298

[12] Prisic S, Hwang H, Dow A, Barnaby O, Pan TS, Lonzanida JA, et al. Zinc regulates a switch between primary and alternative S18 ribosomal proteins in Mycobacterium tuberculosis. Mol Microbiol. 2015;97: 263–280. 10.1111/mmi.13022 25858183 PMC4548965

[13] Garton NJ, Waddell SJ, Sherratt AL, Lee S-M, Smith RJ, Senner C, et al. Cytological and Transcript Analyses Reveal Fat and Lazy Persister-Like Bacilli in Tuberculous Sputum. Neyrolles O, editor. PLoS Med. 2008;5: e75. 10.1371/journal.pmed.0050075 18384229 PMC2276522

[14] Rockwood N, Lai RPJ, Seldon R, Young DB, Wilkinson RJ. Variation in pre-therapy levels of selected Mycobacterium tuberculosis transcripts in sputum and their relationship with 2-month culture conversion. Wellcome Open Res. 2019;4: 106. 10.12688/wellcomeopenres.15332.1

[15] Lim CK, Hassan KA, Penesyan A, Loper JE, Paulsen IT. The effect of zinc limitation on the transcriptome of Pseudomonas protegens Pf-5. Environ Microbiol. 2013;15: 702–715. 10.1111/j.1462-2920.2012.02849.x 22900619

[16] Gopal R, Monin L, Torres D, Slight S, Mehra S, McKenna KC, et al. S100A8/A9 Proteins Mediate Neutrophilic Inflammation and Lung Pathology during Tuberculosis. Am J Respir Crit Care Med. 2013;188: 1137–1146. 10.1164/rccm.201304-0803OC 24047412 PMC3863739

[17] Keren I, Minami S, Rubin E, Lewis K. Characterization and transcriptome analysis of Mycobacterium tuberculosis persisters. MBio. 2011;2: e00100–11. 10.1128/mBio.00100-11 21673191 PMC3119538

[18] Lai RP, Cortes T, Marais S, Rockwood N, Burke ML, Garza-Garcia A, et al. Transcriptomic characterization of tuberculous sputum reveals a host Warburg effect and microbial cholesterol catabolism. BioRxiv. 2020. 10.1101/2020.03.09.983163 PMC8649757 34872348

[19] Karakousis PC, Yoshimatsu T, Lamichhane G, Woolwine SC, Nuermberger EL, Grosset J, et al. Dormancy Phenotype Displayed by Extracellular Mycobacterium tuberculosis within Artificial Granulomas in Mice. J Exp Med. 2004;200: 647–657. 10.1084/jem.20040646 15353557 PMC2212740

[20] Hunter RL. Pathology of post primary tuberculosis of the lung: An illustrated critical review. Tuberculosis. 2011;91: 497–509. 10.1016/j.tube.2011.03.007 21733755 PMC3215852

[21] Li Y, Corro JH, Palmer CD, Ojha AK. Progression from remodeling to hibernation of ribosomes in zinc-starved mycobacteria. Proc Natl Acad Sci. 2020;117: 19528–19537. 10.1073/pnas.2013409117 32723821 PMC7431043

[22] Sigdel TK, Easton JA, Crowder MW. Transcriptional response of Escherichia coli to TPEN. J Bacteriol. 2006;188: 6709–6713. 10.1128/JB.00680-06 16952965 PMC1595494

[23] Tobiasson V, Dow A, Prisic S, Amunts A. Zinc depletion does not necessarily induce ribosome hibernation in mycobacteria. Proc Natl Acad Sci. 2019;116: 2395–2397. 10.1073/pnas.1817490116 30683730 PMC6377477

[24] Mouton JM, Heunis T, Dippenaar A, Gallant JL, Kleynhans L, Sampson SL. Comprehensive Characterization of the Attenuated Double Auxotroph Mycobacterium tuberculosisΔleuDΔpanCD as an Alternative to H37Rv. Front Microbiol. 2019;10: 1–13. 10.3389/fmicb.2019.00001 31481950 PMC6710366

[25] Dow A, Prisic S. Alternative ribosomal proteins are required for growth and morphogenesis of Mycobacterium smegmatis under zinc limiting conditions. Pavelka M, editor. PLoS One. 2018;13: e0196300. 10.1371/journal.pone.0196300 29684089 PMC5912738

[26] Halbeisen RE, Gerber AP. Stress-Dependent Coordination of Transcriptome and Translatome in Yeast. Bähler J, editor. PLoS Biol. 2009;7: e1000105. 10.1371/journal.pbio.1000105 19419242 PMC2675909

[27] Gerashchenko M V, Lobanov A V, Gladyshev VN. Genome-wide ribosome profiling reveals complex translational regulation in response to oxidative stress. Proc Natl Acad Sci. 2012;109: 17394–17399. 10.1073/pnas.1120799109 23045643 PMC3491468

[28] Chen Y-X, Xu Z, Ge X, Sanyal S, Lu ZJ, Javid B. Selective translation by alternative bacterial ribosomes. Proc Natl Acad Sci. 2020;117: 19487–19496. 10.1073/pnas.2009607117 32723820 PMC7431078

[29] Powell SR. The Antioxidant Properties of Zinc. J Nutr. 2000;130: 1447S–1454S. 10.1093/jn/130.5.1447S 10801958

[30] Oteiza PI. Zinc and the modulation of redox homeostasis. Free Radic Biol Med. 2012;53: 1748–1759. 10.1016/j.freeradbiomed.2012.08.568 22960578 PMC3506432

[31] Wu C-Y, Bird AJ, Winge DR, Eide DJ. Regulation of the Yeast TSA1 Peroxiredoxin by ZAP1 Is an Adaptive Response to the Oxidative Stress of Zinc Deficiency. J Biol Chem. 2007;282: 2184–2195. 10.1074/jbc.M606639200 17121842

[32] Oteiza PL, Olin KL, Fraga CG, Keen CL. Zinc Deficiency Causes Oxidative Damage to Proteins, Lipids and DNA in Rat Testes. Biochem Mol Roles Nutr. 1995;125: 823–829. 10.1093/jn/125.4.823 7722683

[33] Wu C-Y, Roje S, Sandoval FJ, Bird AJ, Winge DR, Eide DJ. Repression of Sulfate Assimilation Is an Adaptive Response of Yeast to the Oxidative Stress of Zinc Deficiency. J Biol Chem. 2009;284: 27544–27556. 10.1074/jbc.M109.042036 19656949 PMC2785683

[34] Voskuil MI, Bartek IL, Visconti K, Schoolnik GK. The Response of Mycobacterium Tuberculosis to Reactive Oxygen and Nitrogen Species. Front Microbiol. 2011;2: 1–12. 10.3389/fmicb.2011.00001 21734908 PMC3119406

[35] Gurumurthy M, Rao M, Mukherjee T, Rao SPS, Boshoff HI, Dick T, et al. A novel F 420 -dependent anti-oxidant mechanism protects Mycobacterium tuberculosis against oxidative stress and bactericidal agents. Mol Microbiol. 2013;87: 744–755. 10.1111/mmi.12127 23240649 PMC3567243

[36] Boshoff HIM, Myers TG, Copp BR, McNeil MR, Wilson MA, Barry CE. The Transcriptional Responses of Mycobacterium tuberculosis to Inhibitors of Metabolism. J Biol Chem. 2004;279: 40174–40184. 10.1074/jbc.M406796200 15247240

[37] Bhattacharjee A, Datta R, Gratton E, Hochbaum AI. Metabolic fingerprinting of bacteria by fluorescence lifetime imaging microscopy. Sci Rep. 2017;7: 3743. 10.1038/s41598-017-04032-w 28623341 PMC5473825

[38] Selengut JD, Haft DH. Unexpected Abundance of Coenzyme F420-Dependent Enzymes in Mycobacterium tuberculosis and Other Actinobacteria. J Bacteriol. 2010;192: 5788–5798. 10.1128/JB.00425-10 20675471 PMC2953692

[39] Li X, Wu J, Han J, Hu Y, Mi K. Distinct Responses of Mycobacterium smegmatis to Exposure to Low and High Levels of Hydrogen Peroxide. Chatterji D, editor. PLoS One. 2015;10: e0134595. 10.1371/journal.pone.0134595 26225431 PMC4520597

[40] PaweŁczyk J, Kremer L. The Molecular Genetics of Mycolic Acid Biosynthesis. Microbiol Spectr. 2014;2. 10.1128/microbiolspec.MGM2-0003-2013 26104214

[41] Takayama K, Wang C, Besra GS. Pathway to Synthesis and Processing of Mycolic Acids in Mycobacterium tuberculosis. Clin Microbiol Rev. 2005;18: 81–101. 10.1128/CMR.18.1.81-101.2005 15653820 PMC544180

[42] Sirakova TD, Dubey VS, Deb C, Daniel J, Korotkova TA, Abomoelak B, et al. Identification of a diacylglycerol acyltransferase gene involved in accumulation of triacylglycerol in Mycobacterium tuberculosis under stress. Microbiology. 2006;152: 2717–2725. 10.1099/mic.0.28993-0 16946266 PMC1575465

[43] Bhatt A, Kremer L, Dai AZ, Sacchettini JC, Jacobs WR. Conditional Depletion of KasA, a Key Enzyme of Mycolic Acid Biosynthesis, Leads to Mycobacterial Cell Lysis. J Bacteriol. 2005;187: 7596–7606. 10.1128/JB.187.22.7596-7606.2005 16267284 PMC1280301

[44] Li Z, Kelley C, Collins F, Rouse D, Morris S. Expression of katG in Mycobacterium tuberculosis Is Associated with Its Growth and Persistence in Mice and Guinea Pigs. J Infect Dis. 1998;177: 1030–1035. 10.1086/515254 9534978

[45] Chawla M, Parikh P, Saxena A, Munshi M, Mehta M, Mai D, et al. Mycobacterium tuberculosis WhiB4 regulates oxidative stress response to modulate survival and dissemination in vivo. Mol Microbiol. 2012;85: 1148–1165. 10.1111/j.1365-2958.2012.08165.x 22780904 PMC3438311

[46] Hendon-Dunn CL, Doris KS, Thomas SR, Allnutt JC, Marriott AAN, Hatch KA, et al. A Flow Cytometry Method for Rapidly Assessing Mycobacterium tuberculosis Responses to Antibiotics with Different Modes of Action. Antimicrob Agents Chemother. 2016;60: 3869–3883. 10.1128/AAC.02712-15 26902767 PMC4914659

[47] Piccaro G, Pietraforte D, Giannoni F, Mustazzolu A, Fattorini L. Rifampin Induces Hydroxyl Radical Formation in Mycobacterium tuberculosis. Antimicrob Agents Chemother. 2014;58: 7527–7533. 10.1128/AAC.03169-14 25288092 PMC4249506

[48] Zhang Y, Heym B, Allen B, Young D, Cole S. The catalase—peroxidase gene and isoniazid resistance of Mycobacterium tuberculosis. Nature. 1992;358: 591–593. 10.1038/358591a0 1501713

[49] Driver ER, Ryan GJ, Hoff DR, Irwin SM, Basaraba RJ, Kramnik I, et al. Evaluation of a Mouse Model of Necrotic Granuloma Formation Using C3HeB/FeJ Mice for Testing of Drugs against Mycobacterium tuberculosis. Antimicrob Agents Chemother. 2012;56: 3181–3195. 10.1128/AAC.00217-12 22470120 PMC3370740

[50] Riccardi G, Milano A, Pasca MR, Nies DH. Genomic analysis of zinc homeostasis in Mycobacterium tuberculosis. FEMS Microbiol Lett. 2008;287: 1–7. 10.1111/j.1574-6968.2008.01320.x 18752625

[51] Park H-D, Guinn KM, Harrell MI, Liao R, Voskuil MI, Tompa M, et al. Rv3133c/dosR is a transcription factor that mediates the hypoxic response of Mycobacterium tuberculosis. Mol Microbiol. 2003;48: 833–843. 10.1046/j.1365-2958.2003.03474.x 12694625 PMC1992516

[52] Capdevila DA, Wang J, Giedroc DP. Bacterial Strategies to Maintain Zinc Metallostasis at the Host-Pathogen Interface. J Biol Chem. 2016;291: 20858–20868. 10.1074/jbc.R116.742023 27462080 PMC5076499

[53] Ong CY, Berking O, Walker MJ, McEwan AG. New Insights into the Role of Zinc Acquisition and Zinc Tolerance in Group A Streptococcal Infection. Freitag NE, editor. Infect Immun. 2018;86: e00048–18. 10.1128/IAI.00048-18 29581188 PMC5964520

[54] Serafini A, Pisu D, Palù G, Rodriguez GM, Manganelli R. The ESX-3 Secretion System Is Necessary for Iron and Zinc Homeostasis in Mycobacterium tuberculosis. Delogu G, editor. PLoS One. 2013;8: e78351. 10.1371/journal.pone.0078351 24155985 PMC3796483

[55] Rodriguez GM, Voskuil MI, Gold B, Schoolnik GK, Smith I. ideR, an Essential Gene in Mycobacterium tuberculosis: Role of IdeR in Iron-Dependent Gene Expression, Iron Metabolism, and Oxidative Stress Response. Infect Immun. 2002;70: 3371–3381. 10.1128/iai.70.7.3371-3381.2002 12065475 PMC128082

[56] Tyagi P, Dharmaraja AT, Bhaskar A, Chakrapani H, Singh A. Mycobacterium tuberculosis has diminished capacity to counteract redox stress induced by elevated levels of endogenous superoxide. Free Radic Biol Med. 2015;84: 344–354. 10.1016/j.freeradbiomed.2015.03.008 25819161 PMC4459714

[57] Tiwari S, van Tonder AJ, Vilchèze C, Mendes V, Thomas SE, Malek A, et al. Arginine-deprivation–induced oxidative damage sterilizes Mycobacterium tuberculosis. Proc Natl Acad Sci. 2018;115: 9779–9784. 10.1073/pnas.1808874115 30143580 PMC6166831

[58] Smollett KL, Dawson LF, Davis EO. SigG Does Not Control Gene Expression in Response to DNA Damage in Mycobacterium tuberculosis H37Rv. J Bacteriol. 2011;193: 1007–1011. 10.1128/JB.01241-10 21169493 PMC3028679

[59] Sala C, Forti F, Di Florio E, Canneva F, Milano A, Riccardi G, et al. Mycobacterium tuberculosis FurA Autoregulates Its Own Expression. J Bacteriol. 2003;185: 5357–5362. 10.1128/jb.185.18.5357-5362.2003 12949087 PMC193761

[60] Ahn S, Jung J, Jang I, Madsen EL, Park W. Role of Glyoxylate Shunt in Oxidative Stress Response. J Biol Chem. 2016;291: 11928–11938. 10.1074/jbc.M115.708149 27036942 PMC4882458

[61] Zhong J, Xiao C, Gu W, Du G, Sun X, He Q, et al. Transfer RNAs Mediate the Rapid Adaptation of Escherichia coli to Oxidative Stress. Ibba M, editor. PLOS Genet. 2015;11: e1005302. 10.1371/journal.pgen.1005302 26090660 PMC4474833

[62] Jaeger T, Budde H, Flohé L, Menge U, Singh M, Trujillo M, et al. Multiple thioredoxin-mediated routes to detoxify hydroperoxides in Mycobacterium tuberculosis. Arch Biochem Biophys. 2004;423: 182–191. 10.1016/j.abb.2003.11.021 14871480

[63] Geiman DE, Raghunand TR, Agarwal N, Bishai WR. Differential Gene Expression in Response to Exposure to Antimycobacterial Agents and Other Stress Conditions among Seven Mycobacterium tuberculosis whiB-Like Genes. Antimicrob Agents Chemother. 2006;50: 2836–2841. 10.1128/AAC.00295-06 16870781 PMC1538666

[64] Vicentefranqueira R, Amich J, Marín L, Sánchez C, Leal F, Calera J. The Transcription Factor ZafA Regulates the Homeostatic and Adaptive Response to Zinc Starvation in Aspergillus fumigatus. Genes (Basel). 2018;9: 318. 10.3390/genes9070318 29949939 PMC6070888

[65] Ganief N, Sjouerman J, Albeldas C, Nakedi KC, Hermann C, Calder B, et al. Associating H 2 O 2- and NO-related changes in the proteome of Mycobacterium smegmatis with enhanced survival in macrophage. Emerg Microbes Infect. 2018;7: 1–17. 10.1038/s41426-017-0002-0 30546046 PMC6292918

[66] Idh J, Andersson B, Lerm M, Raffetseder J, Eklund D, Woksepp H, et al. Reduced susceptibility of clinical strains of Mycobacterium tuberculosis to reactive nitrogen species promotes survival in activated macrophages. Neyrolles O, editor. PLoS One. 2017;12: e0181221. 10.1371/journal.pone.0181221 28704501 PMC5509328

[67] Lee H-N, Lee N-O, Han SJ, Ko I-J, Oh J-I. Regulation of the ahpC Gene Encoding Alkyl Hydroperoxide Reductase in Mycobacterium smegmatis. Manganelli R, editor. PLoS One. 2014;9: e111680. 10.1371/journal.pone.0111680 25365321 PMC4218801

[68] Deretic V, Philipp W, Dhandayuthapani S, Mudd M, Curcic R, Garbe T, et al. Mycobacterium tuberculosis is a natural mutant with an inactivated oxidative-stress regulatory gene:implications for sensitivity to isoniazid. Mol Microbiol. 1995;17: 889–900. 10.1111/j.1365-2958.1995.mmi_17050889.x 8596438

[69] Springer B, Master S, Sander P, Zahrt T, McFalone M, Song J, et al. Silencing of Oxidative Stress Response in Mycobacterium tuberculosis: Expression Patterns of ahpC in Virulent and Avirulent Strains and Effect ofahpC Inactivation. Kaufmann SHE, editor. Infect Immun. 2001;69: 5967–5973. 10.1128/IAI.69.10.5967-5973.2001 11553532 PMC98723

[70] Master SS, Springer B, Sander P, Boettger EC, Deretic V, Timmins GS. Oxidative stress response genes in Mycobacterium tuberculosis: role of ahpC in resistance to peroxynitrite and stage-specific survival in macrophages. Microbiology. 2002;148: 3139–3144. 10.1099/00221287-148-10-3139 12368447

[71] Via LE, Lin PL, Ray SM, Carrillo J, Allen SS, Eum SY, et al. Tuberculous Granulomas Are Hypoxic in Guinea Pigs, Rabbits, and Nonhuman Primates. Infect Immun. 2008;76: 2333–2340. 10.1128/IAI.01515-07 18347040 PMC2423064

[72] Sharma S, Ryndak MB, Aggarwal AN, Yadav R, Sethi S, Masih S, et al. Transcriptome analysis of mycobacteria in sputum samples of pulmonary tuberculosis patients. Neyrolles O, editor. PLoS One. 2017;12: e0173508. 10.1371/journal.pone.0173508 28282458 PMC5345810

[73] Pym AS, Domenech P, Honore N, Song J, Deretic V, Cole ST. Regulation of catalase-peroxidase (KatG) expression, isoniazid sensitivity and virulence by furA of Mycobacterium tuberculosis. Mol Microbiol. 2001;40: 879–889. 10.1046/j.1365-2958.2001.02427.x 11401695

[74] Kieser KJ, Rubin EJ. How sisters grow apart: mycobacterial growth and division. Nat Rev Microbiol. 2014;12: 550–562. 10.1038/nrmicro3299 24998739 PMC6556109

[75] Mahamed D, Boulle M, Ganga Y, Mc Arthur C, Skroch S, Oom L, et al. Intracellular growth of Mycobacterium tuberculosis after macrophage cell death leads to serial killing of host cells. Elife. 2017;6: 1–26. 10.7554/eLife.22028 28130921 PMC5319838

[76] Ehrt S, Schnappinger D. Mycobacterial survival strategies in the phagosome: defence against host stresses. Cell Microbiol. 2009;11: 1170–1178. 10.1111/j.1462-5822.2009.01335.x 19438516 PMC3170014

[77] Ng VH, Cox JS, Sousa AO, MacMicking JD, McKinney JD. Role of KatG catalase-peroxidase in mycobacterial pathogenesis: countering the phagocyte oxidative burst. Mol Microbiol. 2004;52: 1291–1302. 10.1111/j.1365-2958.2004.04078.x 15165233

[78] Pradhan A, Avelar GM, Bain JM, Childers D, Pelletier C, Larcombe DE, et al. Non-canonical signalling mediates changes in fungal cell wall PAMPs that drive immune evasion. Nat Commun. 2019;10: 5315. 10.1038/s41467-019-13298-9 31757950 PMC6876565

[79] Eoh H, Wang Z, Layre E, Rath P, Morris R, Branch Moody D, et al. Metabolic anticipation in Mycobacterium tuberculosis. Nat Microbiol. 2017;2: 17084. 10.1038/nmicrobiol.2017.84 28530656 PMC5540153

[80] Mitchell A, Romano GH, Groisman B, Yona A, Dekel E, Kupiec M, et al. Adaptive prediction of environmental changes by microorganisms. Nature. 2009;460: 220–224. 10.1038/nature08112 19536156

[81] Dhar N, McKinney J, Manina G. Phenotypic Heterogeneity in Mycobacterium tuberculosis. Microbiol Spectr. 2016;4: 1–27. 10.1128/microbiolspec.TBTB2-0021-2016 27837741

