# Supplementary material for: Zinc limitation triggers anticipatory adaptations in Mycobacterium tuberculosis
Source: PLoS Pathog. 2021 May 14;17(5):e1009570. doi: 10.1371/journal.ppat.1009570 (PMC8121289; doi:10.1371/journal.ppat.1009570)
Supplement: S1 Fig — (PDF) [file ppat.1009570.s001.pdf]

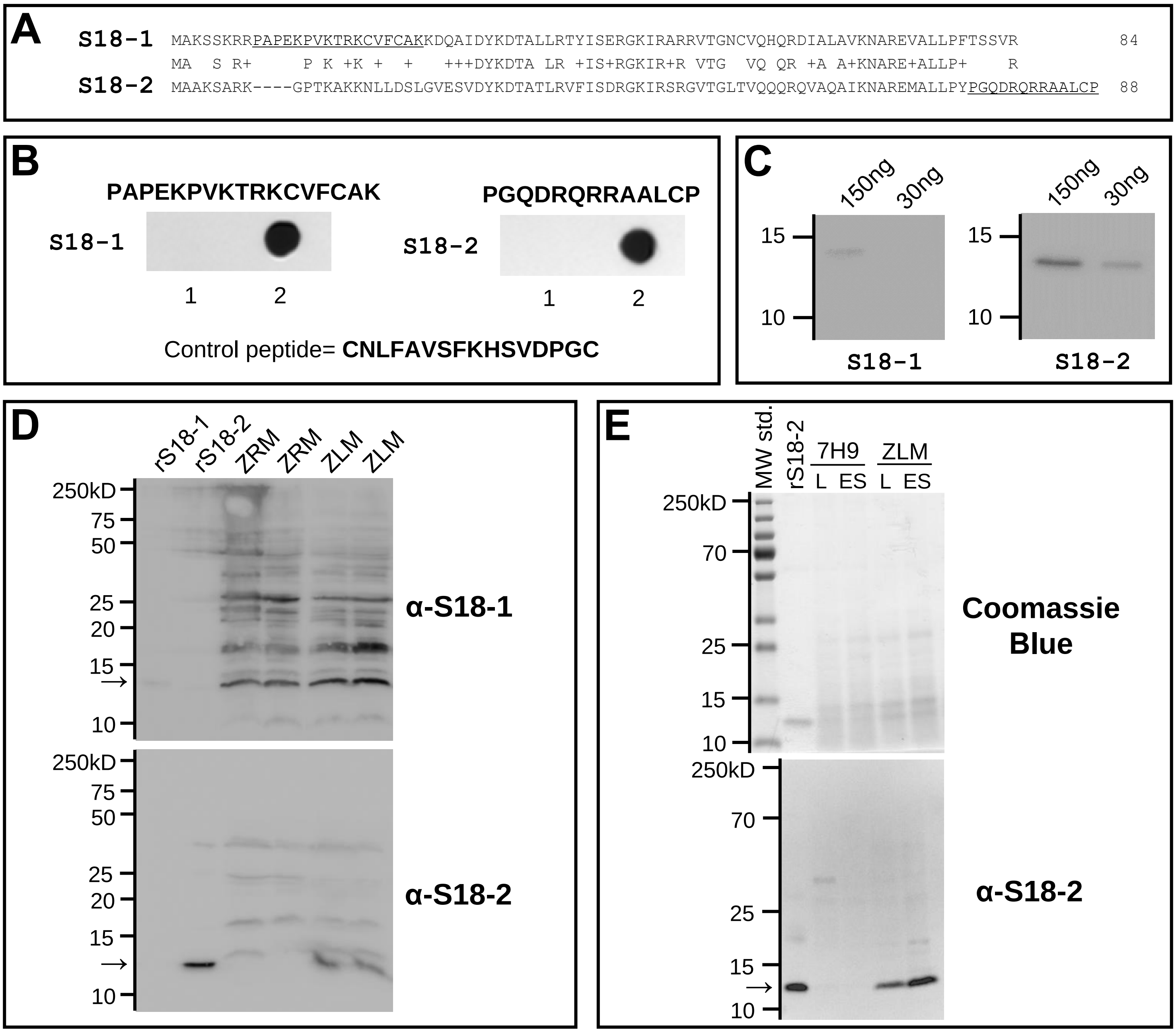

S1 Fig. Validation of rabbit polyclonal antibodies raised against *Mtb* ribosomal proteins S18-1 and S18-2.
(A) Alignment S18-1 and S18-2 protein sequences with the peptide sequence selected as the antigen used to raise polyclonal antibodies underlined for each protein. (B) Validation of antibody recognition of the synthetic peptide antigen. For both S18-1 and S18-2 antibodies, 100 ng of the control peptide (lane 1) or synthetic peptide (lane 2) was detected with the corresponding antibody diluted 1:1000. Control peptide sequence (same for both dot blots) is given at the bottom and the sequence of the synthetic peptide used for each antibody is given above the respective dot blot. (C) Detection of recombinant S18-1 (rS18-1) using anti-S18-1 and recombinant S18-2 (rS18-2) using anti-S18-2 polyclonal antibodies. Recombinant proteins have a 6xHis-tag. Two different loading amounts for each recombinant protein are given in nanograms (ng). (D) Specificity of detection for anti-S18-1 and anti-S18-2 against lysates from *Mtb* mc^2 6206 day 10 ZRM and ZLM cultures. For both conditions, 150 ng of recombinant proteins with 6xHis-tag (marked with arrows), 10 µg lysates for anti-S18-1 and 40 µg lysates for anti-S18-2 are loaded. Cells from ZRM and ZLM are biological replicates from two independent growths. For panels C and D, proteins are resolved on 5%/15% sodium dodecyl sulfate polyacrylamide gels. (E) Specific detection of S18-2 protein (marked with arrow) in ZLM and its absence in the standard, zinc-replete 7H9/ADC media in log and early stationary growth phases. Coomassie Blue staining of an identical SDS-PAGE gel is given to control for loading amount (25 ng untagged recombinant S18-2 protein, 8 µg *Mtb* H37Rv lysates). Proteins were resolved on Any kD™ Mini-PROTEAN® TGX™ Precast Protein Gels (BioRad). Recombinant proteins and growth of *Mtb* H37Rv in panel E are described in S1 Text, SI References [1].
