# Supplementary material for: Zinc limitation triggers anticipatory adaptations in Mycobacterium tuberculosis
Source: PLoS Pathog. 2021 May 14;17(5):e1009570. doi: 10.1371/journal.ppat.1009570 (PMC8121289; doi:10.1371/journal.ppat.1009570)
Supplement: S2 Fig — (PDF) [file ppat.1009570.s002.pdf]

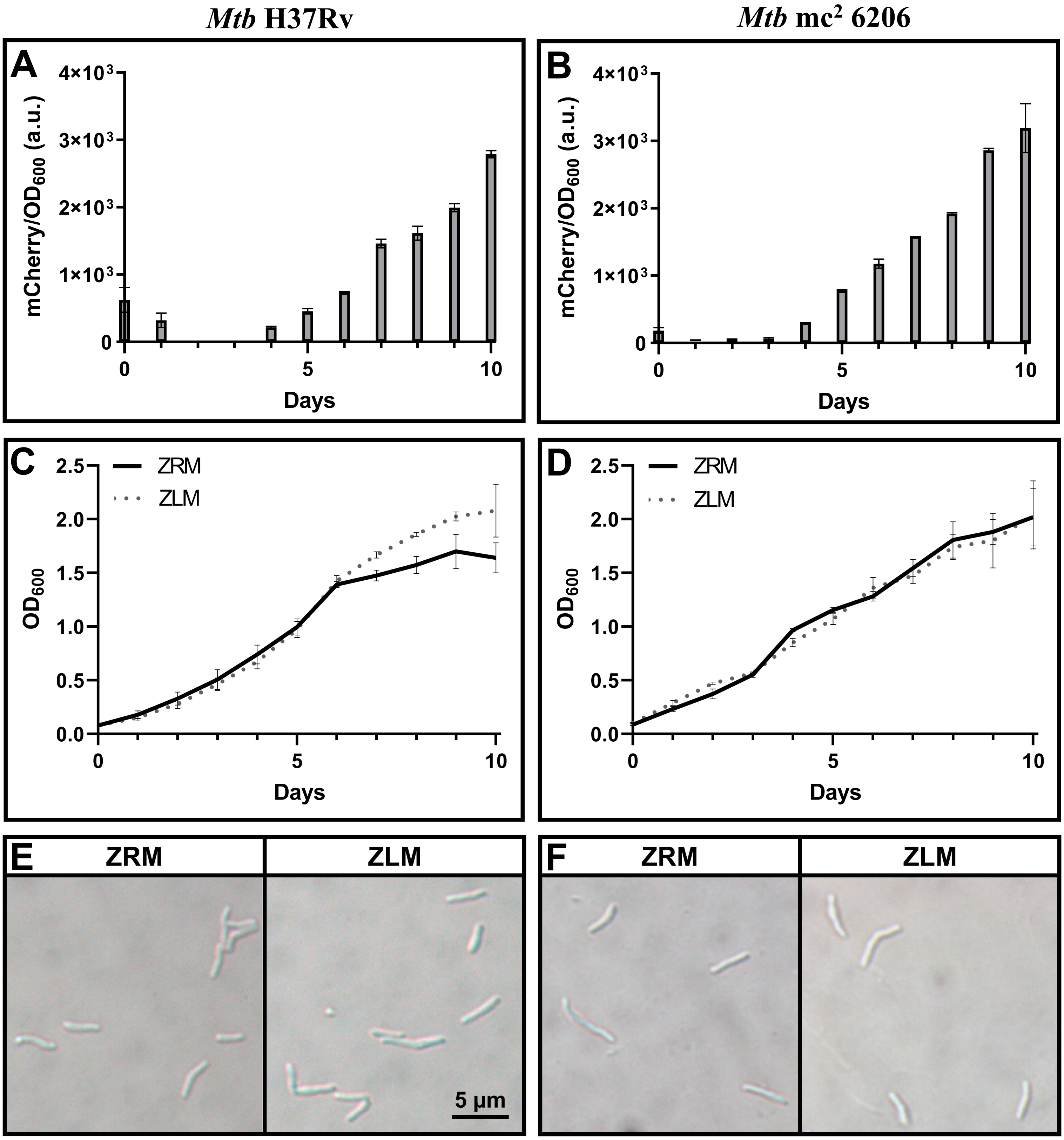

S2 Fig. Growth and morphology of *Mtb* H37Rv and mc² 6206 in ZLM and ZRM. Fluorescence from P_{altRP}-mCherry reporter plasmid carried by *Mtb* H37Rv (A) and *Mtb* mc² 6206 (B) throughout growth in ZLM, normalized to OD_{600} of the reporter strains at each time point. Fluorescence above background was not detected at any point during growth from *Mtb* H37Rv and mc² 6206 strains carrying P_{altRP}-mCherry reporter plasmid when grown in ZRM. Optical density detected at 600nm (OD_{600}) for *Mtb* H37Rv (C) and *Mtb* mc² 6206 (D) cultures grown in ZRM and ZLM. The increased variation in OD_{600} measurements in late-log phase is due to increased clumping of cultures in both ZRM and ZLM. Data points for A-D represent the mean of cultures grown in biological triplicate and error bars represent the standard deviation. Differential interference contrast (DIC) microscopy of *Mtb* H37Rv (E) and *Mtb* mc² 6206 (F) cultures after 10 days of growth in ZRM or ZLM. *Mtb* H37Rv cultures were fixed in paraformaldehyde before being removed from the BSL3 facility for analysis, mc² 6206 cultures were not fixed. The scale bar in panel E corresponds to all micrographs. There was no significant difference in the length of cells from ZRM vs. ZLM for either H37Rv (t-test, p-value=0.48) or mc² 6206 (t-test, p-value=0.34). The average cell length and standard deviation in micrometers from 100 individual cells from cultures at day 10 are: H37Rv ZRM= 3.51 ±0.78, H37Rv ZLM= 3.51 ±0.91, mc² 6206 ZRM = 3.76 ±1.12, and mc² 6206 ZLM = 3.69 ±1.34. Cell length measurements were made following the methods described in the main text References [25].
