# Supplementary material for: Zinc limitation triggers anticipatory adaptations in Mycobacterium tuberculosis
Source: PLoS Pathog. 2021 May 14;17(5):e1009570. doi: 10.1371/journal.ppat.1009570 (PMC8121289; doi:10.1371/journal.ppat.1009570)
Supplement: S3 Fig — (PDF) [file ppat.1009570.s003.pdf]

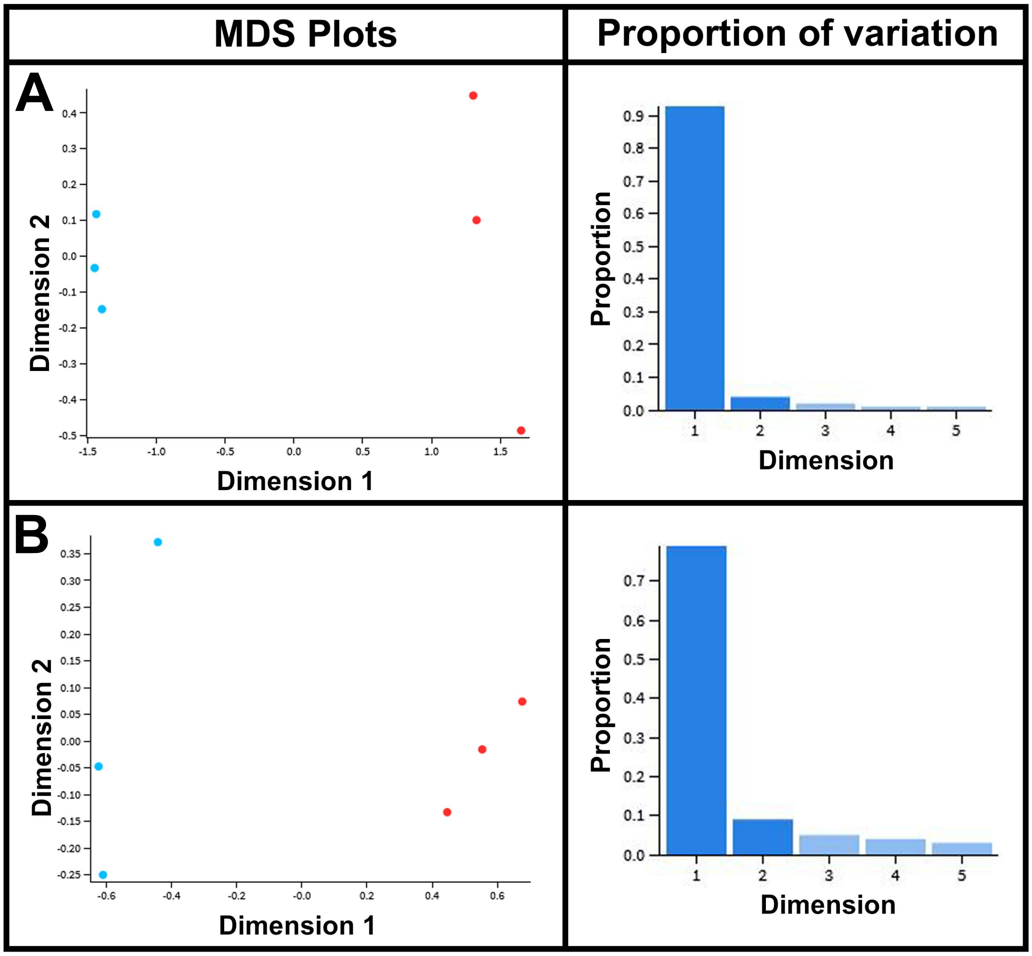

S3 Fig. Multi-dimensional scaling (MDS) plots for *Mtb* H37Rv transcriptomics (A) and proteomics (B). Cultures were analyzed from biological triplicate and blue dots represent samples from ZRM while red dots represent samples from ZLM. For each MDS plot the first two leading dimensions describing variation in the data are shown and the bar graphs on the right of each panel give the proportion of variation in the data described by each dimension (including dimensions 3-5 which are not shown in the MDS plots).
