# Supplementary material for: Zinc limitation triggers anticipatory adaptations in Mycobacterium tuberculosis
Source: PLoS Pathog. 2021 May 14;17(5):e1009570. doi: 10.1371/journal.ppat.1009570 (PMC8121289; doi:10.1371/journal.ppat.1009570)
Supplement: S4 Fig — (PDF) [file ppat.1009570.s004.pdf]

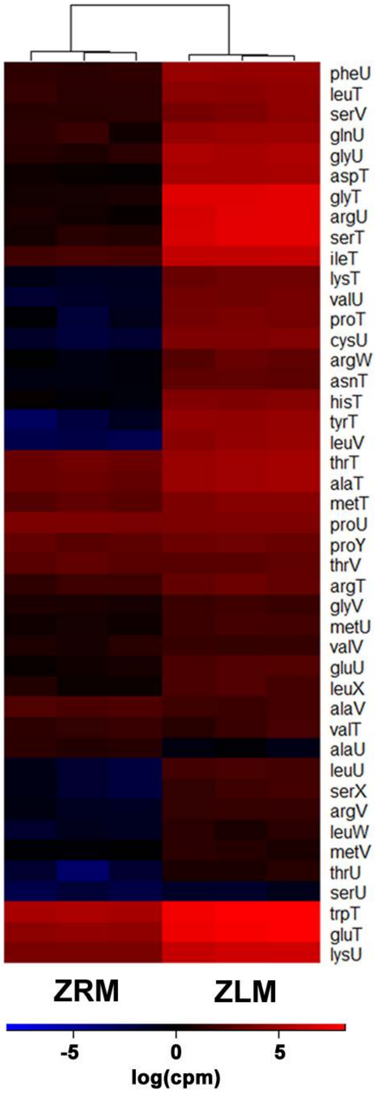

S4 Fig. Heatmap of tRNA molecules detected in RNAseq of *Mtb* H37Rv. Heatmap showing normalized log counts per million, log(cpm), expression values for tRNA molecules detected in RNAseq analysis. The heatmap has not been scaled and represents the normalized log(cpm) values used for differential expression analysis with values for biological triplicates given in columns labeled ZRM and ZLM.
