# Supplementary material for: Zinc limitation triggers anticipatory adaptations in Mycobacterium tuberculosis
Source: PLoS Pathog. 2021 May 14;17(5):e1009570. doi: 10.1371/journal.ppat.1009570 (PMC8121289; doi:10.1371/journal.ppat.1009570)
Supplement: S5 Fig — (PDF) [file ppat.1009570.s005.pdf]

A

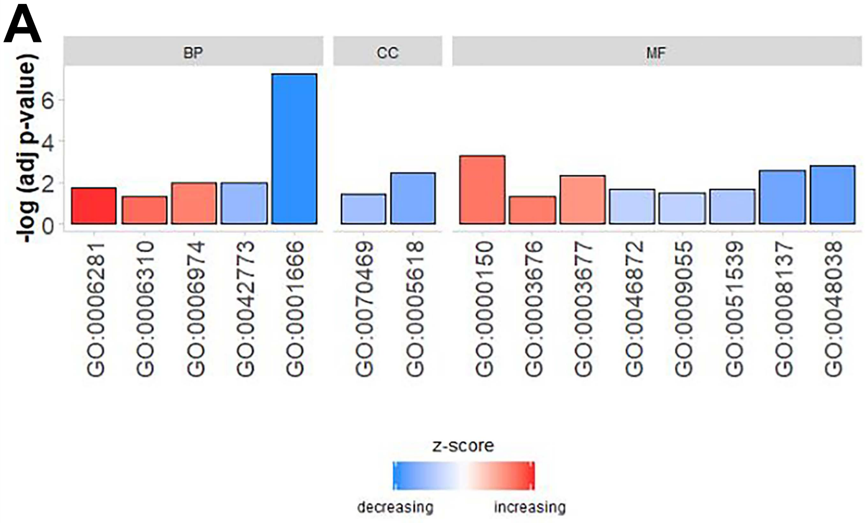

GO ID	Term	Process	p-value
GO:0001666	response to hypoxia	BP	5.48E-08
GO:0000150	recombinase activity	MF	5.10E-04
GO:0048038	quinone binding	MF	1.49E-03
GO:0008137	NADH dehydrogenase (ubiquinone) activity	MF	2.79E-03
GO:0005618	cell wall	CC	3.30E-03
GO:0003677	DNA binding	MF	4.36E-03
GO:0042773	ATP synthesis coupled electron transport	BP	1.01E-02
GO:0006974	cellular response to DNA damage stimulus	BP	1.07E-02
GO:0006281	DNA repair	BP	1.86E-02
GO:0046872	metal ion binding	MF	2.12E-02
GO:0051539	4 iron, 4 sulfur cluster binding	MF	2.21E-02
GO:0009055	electron carrier activity	MF	3.23E-02
GO:0070469	respiratory chain	CC	3.82E-02
GO:0006310	DNA recombination	BP	4.51E-02
GO:0003676	nucleic acid binding	MF	4.79E-02

B

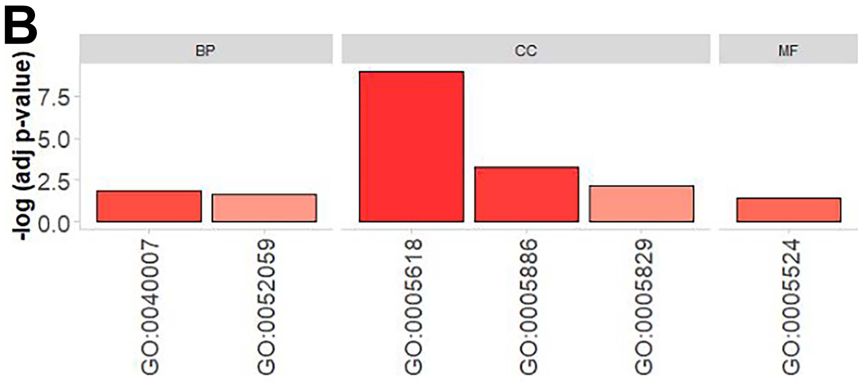

GO ID	Term	Process	p-value
GO:0005618	cell wall	CC	1.05E-09
GO:0005886	plasma membrane	CC	5.42E-04
GO:0005829	cytosol	CC	7.34E-03
GO:0040007	growth	BP	1.39E-02
GO:0052059	evasion or tolerance by symbiont of host-produced reactive oxygen species	BP	2.21E-02
GO:0005524	ATP binding	MF	4.04E-02

S5 Fig. Gene ontology enrichment analysis of differentially expressed genes (A) and proteins (B) from *Mtb* H37Rv. Bar graphs of significantly enriched GO terms (p-value <0.05) in the list of differentially expressed genes (A) or proteins (B). For panel A, among the 725 DE genes in ZLM, 663 are protein coding genes with annotated GO terms and were used for the enrichment analysis and for panel B, all 65 DE proteins had GO annotations and were used in the enrichment analysis. The color of the bars is determined by the z-score and indicates whether the given biological process is more likely to be decreased (blue) or increased (red) in the dataset. The scale for the z-score in panel A applies to both bar graphs in panels A and B. A key for the GO terms, their process and the p-value for the enriched term is given to the right of each bar graph. Process refers to the GO term categories labeled above each bar graph which refers to the process belonging to either a cellular component (CC), biological process (BP) or molecular function (MF).
