# Supplementary material for: Zinc limitation triggers anticipatory adaptations in Mycobacterium tuberculosis
Source: PLoS Pathog. 2021 May 14;17(5):e1009570. doi: 10.1371/journal.ppat.1009570 (PMC8121289; doi:10.1371/journal.ppat.1009570)
Supplement: S6 Fig — (PDF) [file ppat.1009570.s006.pdf]

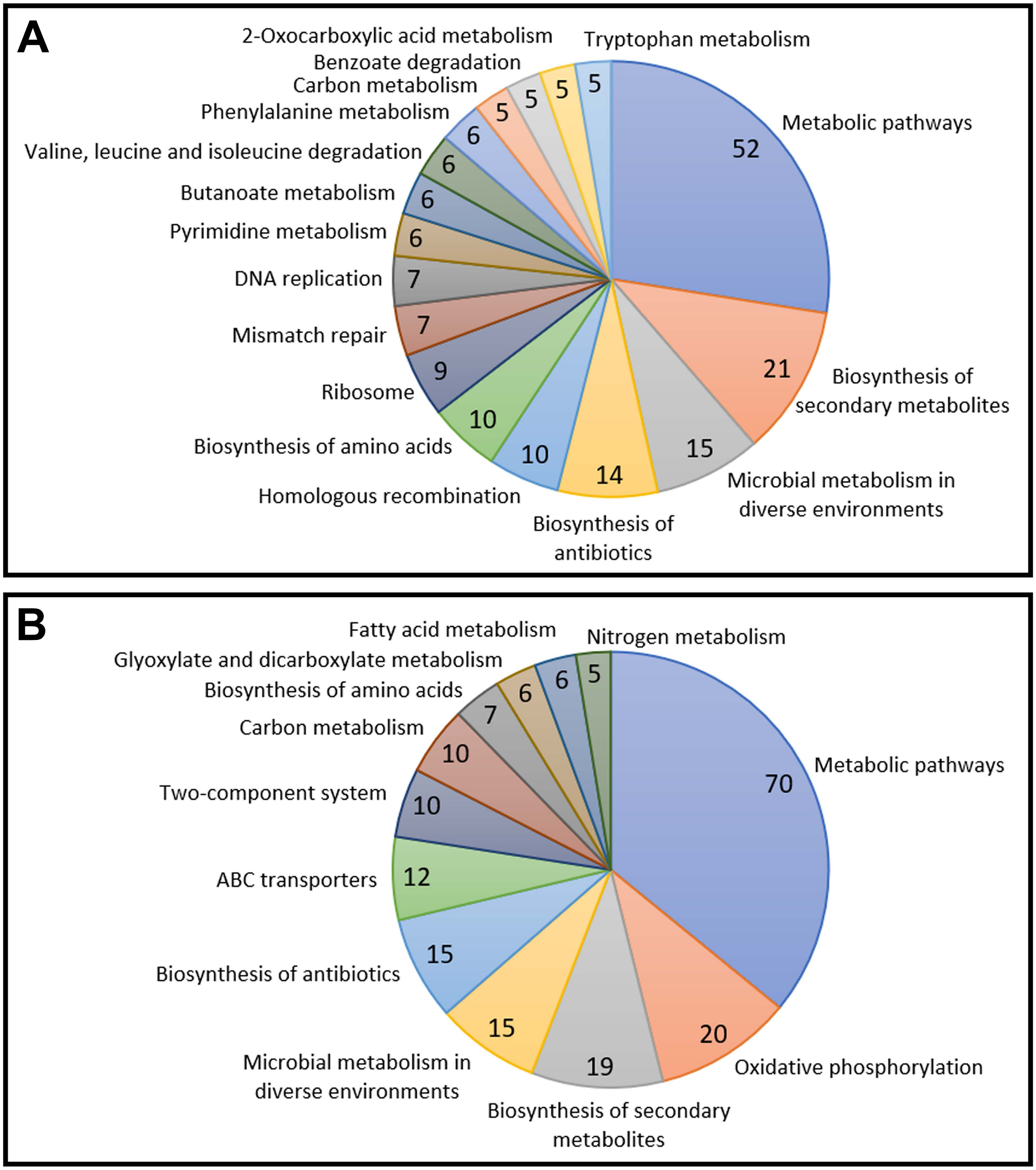


**S6 Fig. Pie charts showing KEGG pathways of DE genes in ZLM vs. ZRM.** (A) Significantly upregulated genes in ZLM. (B) Significantly downregulated genes in ZLM. For both charts, each slice of the pie is labeled with the KEGG pathway and the number inside the pie slice represents the number of genes in that pathway that were significantly regulated. Only pathways containing 5 or more DE genes are shown in the charts, the complete list of DE genes with KEGG pathway annotations are given in S6 and S7 Tables.
