# Supplementary material for: Zinc limitation triggers anticipatory adaptations in Mycobacterium tuberculosis
Source: PLoS Pathog. 2021 May 14;17(5):e1009570. doi: 10.1371/journal.ppat.1009570 (PMC8121289; doi:10.1371/journal.ppat.1009570)
Supplement: S7 Fig — (PDF) [file ppat.1009570.s007.pdf]

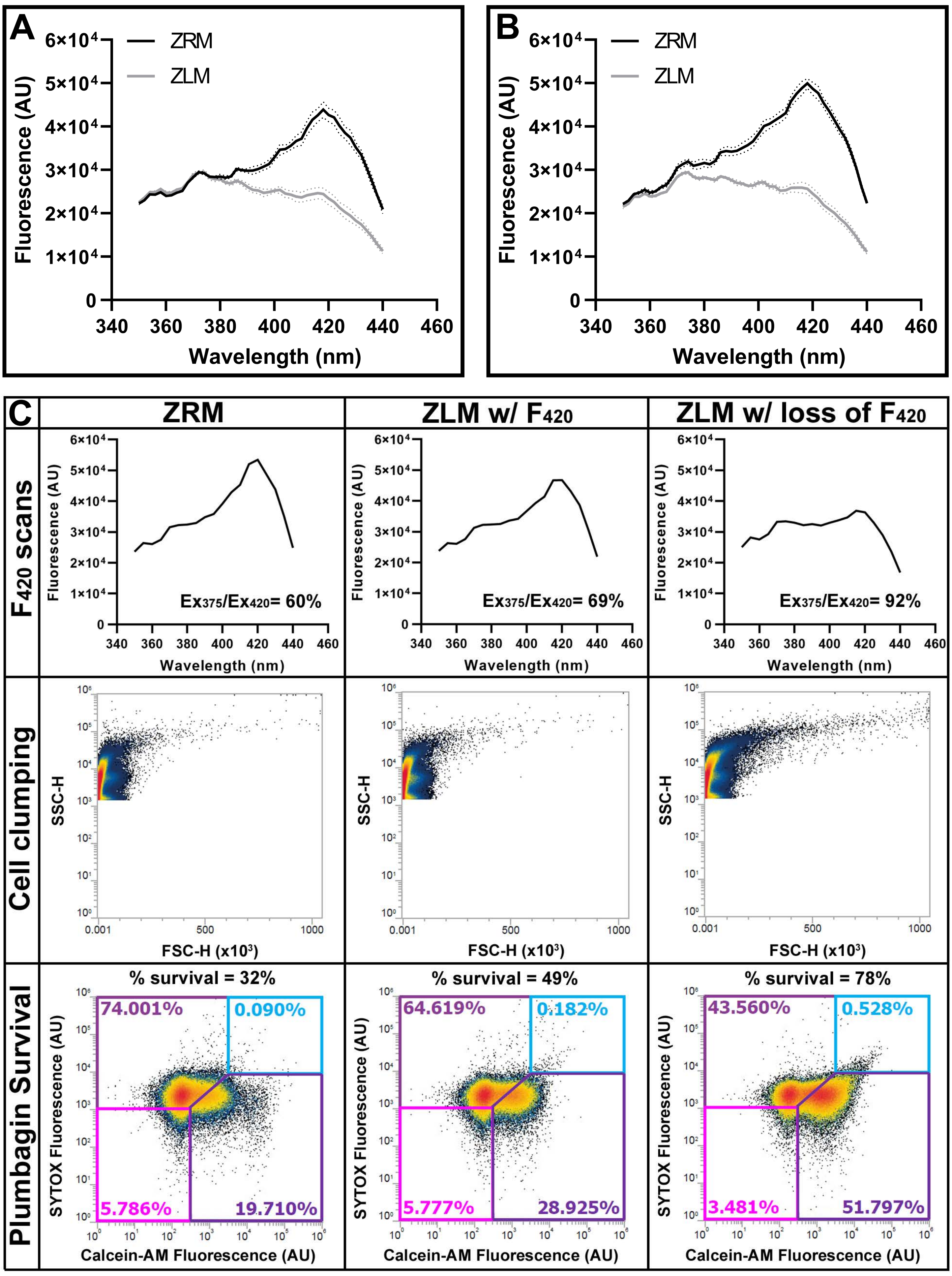

S7 Fig. Loss of peak fluorescence from redox cofactor F_{420} under Zn^{2+}-limiting conditions and the association of F_{420} loss with Zn^{2+}-limited phenotypes. Excitation wavelength scans of redox cofactor F_{420} in *Mtb* mc^2 6206 cultures (A) and cell-free supernatants (B) after 10 days of growth in ZRM and ZLM. All scans have emission wavelength of 475 nm. Solid lines represent the average of three biological replicates and dotted lines represent the standard deviation. (C) Zn^{2+}-limited phenotypes are associated with loss of F_{420} peak fluorescence. Three representative biological samples are shown to demonstrate phenotypes of cells grown to late-log phase (10 days) in ZRM and ZLM. Typically, cultures grown in ZLM exhibited a loss of peak fluorescence at 420 nm (ZLM w/ loss of F_{420}, panels in column on the right). However on occasion, a culture grown in ZLM retained some, albeit reduced fluorescence at 420 nm (ZLM w/F_{420}, panels in the middle column). The percentage of fluorescence intensity at excitation 375 nm to 420 nm (emission at 475 nm), was a quantifiable way to define loss of F_{420} peak fluorescence and can be used in the absence of equipment with fluorescence scanning ability. Cultures grown in ZRM had highly reproducible percentages of Ex_{375}/Ex_{420} with an average of 58% and standard deviation of 2% (n=6). Cultures grown in ZLM that lost peak fluorescence from F_{420} had and average Ex_{375}/Ex_{420} of 98% and standard deviation of 8% (n=5). An example of a culture that was grown in ZLM but retained some (albeit reduced) peak fluorescence at 420 nm had an average Ex_{375}/Ex_{420} of 69%. The percentages of Ex_{375}/Ex_{420} given in the graphs showing F_{420} scans are the ratios from the representative cultures shown. We noticed that the phenotype of Zn^{2+}-limited cells (ZLM) correlated with the loss of fluorescence from F_{420} and the culture that had retained some F_{420} fluorescence (panels in middle column) exhibited a phenotype that was in between that of cultures from ZRM and those from ZLM that had lost fluorescence from F_{420}. Phenotypes were observed using flow cytometry where cell clumping (after enrichment for single cell suspensions, see S1 Text) is demonstrated by increased amount of particles detected at larger forward scatter peak height intensities (FSC-H). Cultures from ZRM and from ZLM with retained F_{420} fluorescence (panels in middle column) have less clumping than cultures from ZLM that have lost F_{420} fluorescence. Similarly, survival after four days of treatment with the oxidizing agent plumbagin correlates with loss of peak F_{420} fluorescence in ZLM cultures as shown in the bottom scatter plots. The percent (%) survival on the top of each graph is calculated by taking the percentage of live cells after treatment vs. untreated cultures at the beginning of the experiment. Refer to S1 Text and S10 Fig for more information regarding experiments with flow cytometry.
