# Supplementary material for: Zinc limitation triggers anticipatory adaptations in Mycobacterium tuberculosis
Source: PLoS Pathog. 2021 May 14;17(5):e1009570. doi: 10.1371/journal.ppat.1009570 (PMC8121289; doi:10.1371/journal.ppat.1009570)
Supplement: S8 Fig — (PDF) [file ppat.1009570.s008.pdf]

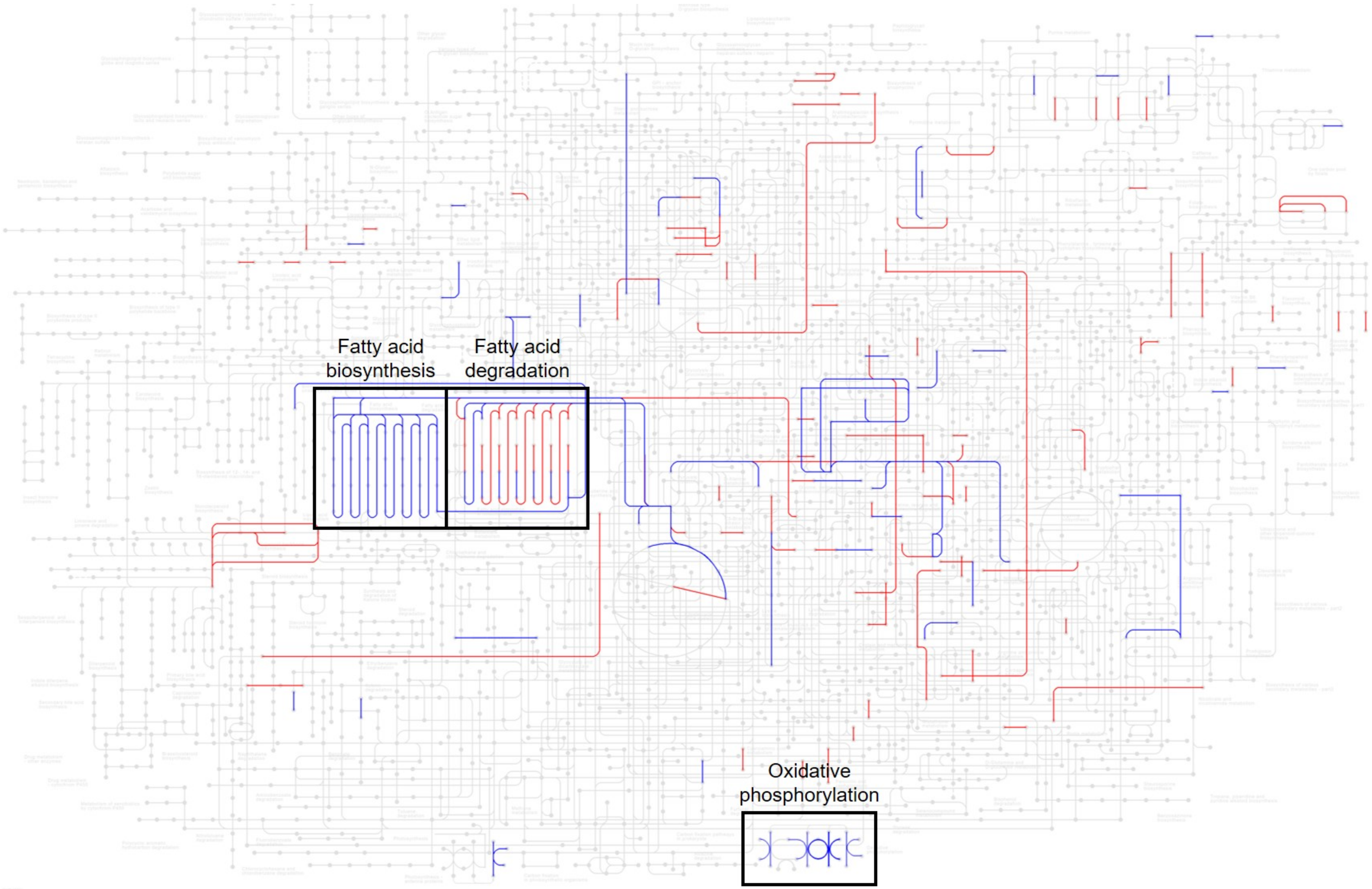

S8 Fig. Genes belonging to KEGG metabolic pathways that are upregulated (red) or downregulated (blue) in ZLM vs. ZRM are superimposed onto a global metabolic network. Enzymes (genes) are represented as lines and their substrates and products are represented by nodes, only genes that were significantly DE are colored. Relevant pathways are labeled on the map. DE genes shown belong to KEGG pathway "mtu01100 metabolic pathways" (S6 and S7 Tables).
