# Supplementary material for: Zinc limitation triggers anticipatory adaptations in Mycobacterium tuberculosis
Source: PLoS Pathog. 2021 May 14;17(5):e1009570. doi: 10.1371/journal.ppat.1009570 (PMC8121289; doi:10.1371/journal.ppat.1009570)
Supplement: S9 Fig — (PDF) [file ppat.1009570.s009.pdf]

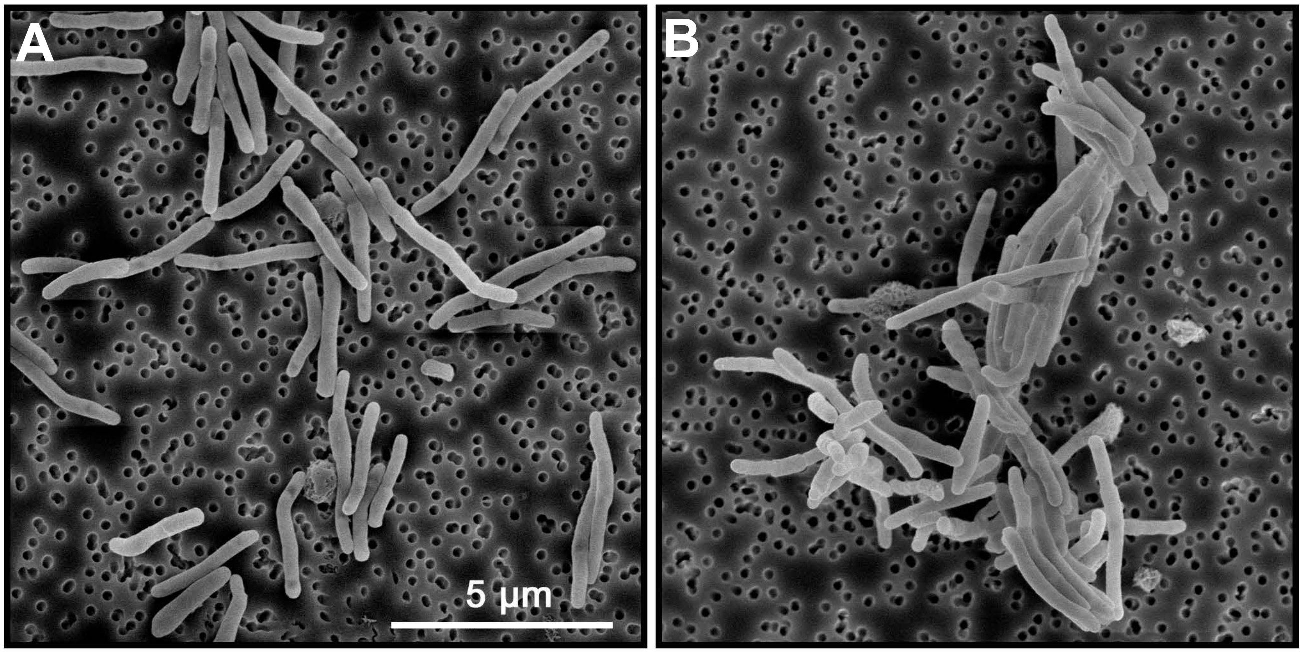

S9 Fig. Clumping of cells in ZLM observed with scanning electron microscopy (SEM). *Mtb* mc^2 6206 cells were grown to late-log phase (Day 10) in ZRM or ZLM, enriched for single cells and prepared for SEM as described in S1 Text. The micrographs shown are representative fields of view observed from cultures in ZRM (A) or ZLM (B) at 5 kV accelerating voltage and 5kX magnification. The scale bar in (A) corresponds to both panels. Holes in the background of the images are the pores of the membranes used for sample preparation.
