# Supplementary material for: Zinc limitation triggers anticipatory adaptations in Mycobacterium tuberculosis
Source: PLoS Pathog. 2021 May 14;17(5):e1009570. doi: 10.1371/journal.ppat.1009570 (PMC8121289; doi:10.1371/journal.ppat.1009570)
Supplement: S10 Fig — (PDF) [file ppat.1009570.s010.pdf]

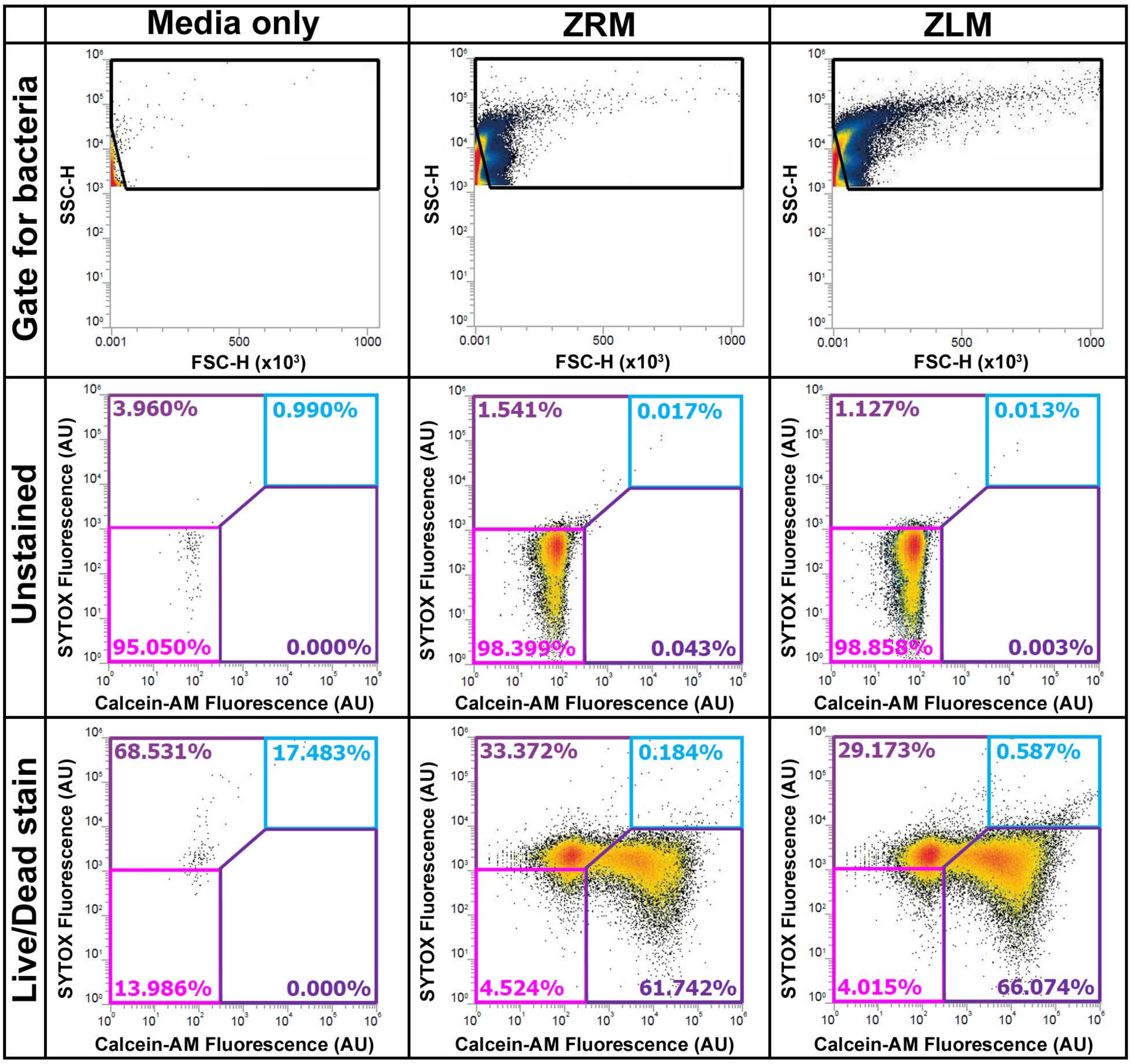

S10 Fig. Controls for flow cytometry killing experiment showing gating strategy for bacteria based on forward vs. side scatter and bacteria stained with the live cell stain Calcein-AM (C-AM) and the dead cell stain SYTOX. Scatter plots on the top row show the height of forward scatter (FSC-H) vs. height of side scatter (SSC-H) signal for particles detected in media only (no bacteria, left panels) and bacteria from ZRM (middle panels) and ZLM (right panels). The black gate indicates the events applied to the gates for C-AM and SYTOX in the graphs below and was drawn to minimize background signal from particles in the media. The number of particles from media only that fall within the gate are fewer than 0.002% of the particles gated in samples with bacteria. The panels in the middle row show scatter plots of unstained media and bacteria from ZRM and ZLM with gates drawn based on C-AM and SYTOX fluorescence. Most particles from unstained samples are C-AM$^-$/SYTOX$^-$ (pink gate, lower left), with less than 5% of particles with fluorescence values that were outside of this gate. The panels on the bottom row shows scatter plots of media only and bacteria from late-log phase in ZRM or ZLM, enriched for single cell suspensions and normalized, then stained with C-AM/SYTOX. The gates for C-AM$^+$/SYTOX$^-$ (purple gate, lower right) indicate the percentage of live cells in the cultures while the gate for C-AM$^-$/SYTOX$^+$ (mauve gate, upper left) indicates the percentage of dead cells in the cultures. Some cells stain positive for both C-AM and SYTOX (cyan gate, upper right) and we excluded cells in this gate from analysis. The gates for C-AM and SYTOX were drawn so that the percentage of particles falling in the unstained gate (C-AM$^-$/SYOTX$^-$, pink) was less than 5%. The percentages given within each gate for the scatter plots represent the percentage of events that fell within that gate. The percentage of cells in the live quadrant (C-AM$^+$/SYTOX$^-$) represents the percentage of live cells at the beginning of the experiment and this value for each culture was used to calculate the percent survival of antibiotic treated cells throughout treatment.
