# Supplementary material for: Zinc limitation triggers anticipatory adaptations in Mycobacterium tuberculosis
Source: PLoS Pathog. 2021 May 14;17(5):e1009570. doi: 10.1371/journal.ppat.1009570 (PMC8121289; doi:10.1371/journal.ppat.1009570)
Supplement: S11 Fig — (PDF) [file ppat.1009570.s011.pdf]

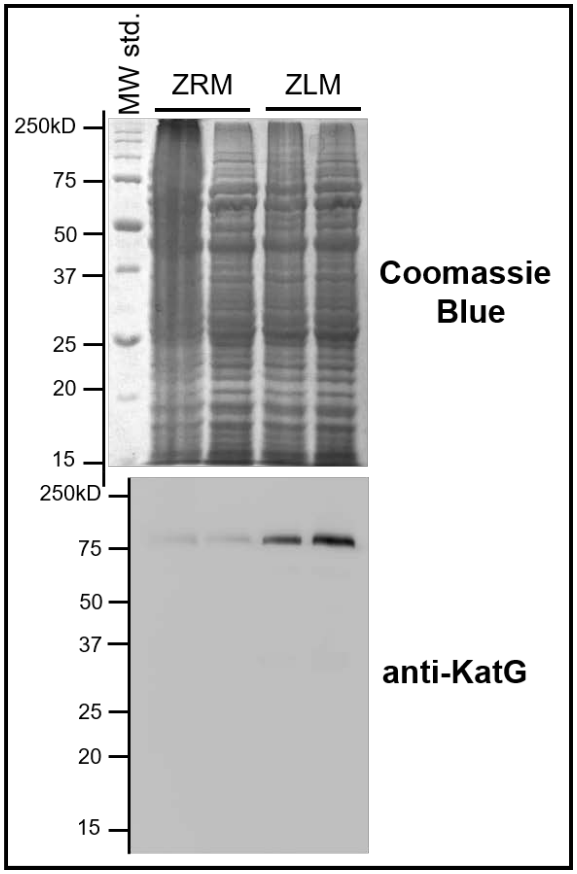

S11 Fig. Detection of KatG protein in lysates from *Mtb* mc^2 6206 in ZRM and ZLM. Proteins from day 10 cultures from two independent growths in ZRM and ZLM were extracted using TRIzol™ and 10 µg protein per sample were resolved on a 5%/12% sodium dodecyl sulfate polyacrylamide gel. Two identical gels were resolved, one gel was stained with Coomassie Blue as a control for protein loading amount and the other was used for blotting.
