# Supplementary material for: Zinc limitation triggers anticipatory adaptations in Mycobacterium tuberculosis
Source: PLoS Pathog. 2021 May 14;17(5):e1009570. doi: 10.1371/journal.ppat.1009570 (PMC8121289; doi:10.1371/journal.ppat.1009570)
Supplement: S12 Fig — (PDF) [file ppat.1009570.s012.pdf]

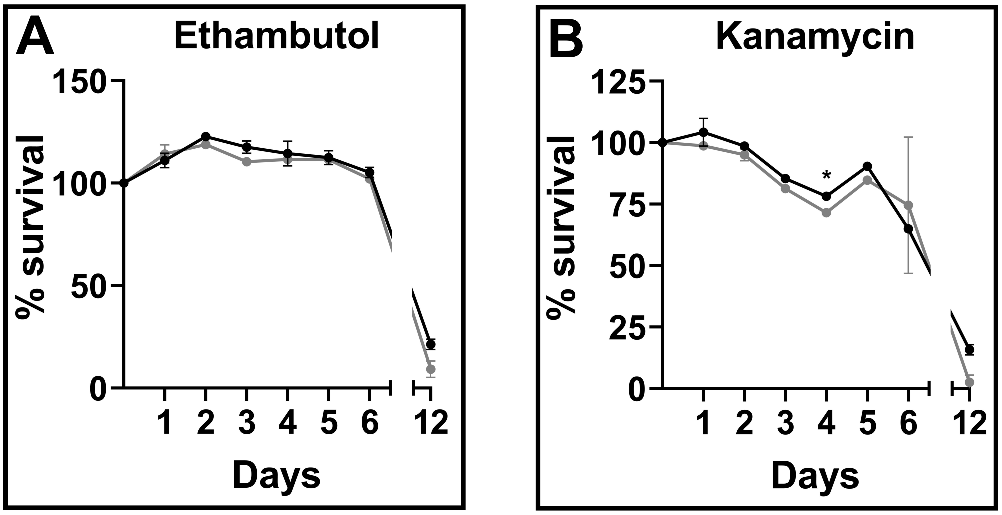

S12 Fig. Survival of *Mtb* mc² 6206 after growth in ZRM (black) or ZLM (grey) and subsequent exposure to the indicated antibiotics. Ethambutol (A) was at a concentration of 200 µg/mL and Kanamycin (B) was at a concentration of 6 µg/mL. Survival was monitored with flow cytometry for twelve days following treatment and was calculated as the percentage of live cells in untreated cultures at the beginning of treatment (refer to S10 Fig). The data is represented as the average of biological replicates (ZRM n=3, ZLM n=2) and the error bars represent the standard deviation. Asterisks represent a statistically significant difference (t-test, p-value <0.05) between survival of cultures in ZRM vs. ZLM at any given time point.
