# Supplementary material for: Zinc limitation triggers anticipatory adaptations in Mycobacterium tuberculosis
Source: PLoS Pathog. 2021 May 14;17(5):e1009570. doi: 10.1371/journal.ppat.1009570 (PMC8121289; doi:10.1371/journal.ppat.1009570)
Supplement: S13 Fig — (PDF) [file ppat.1009570.s013.pdf]

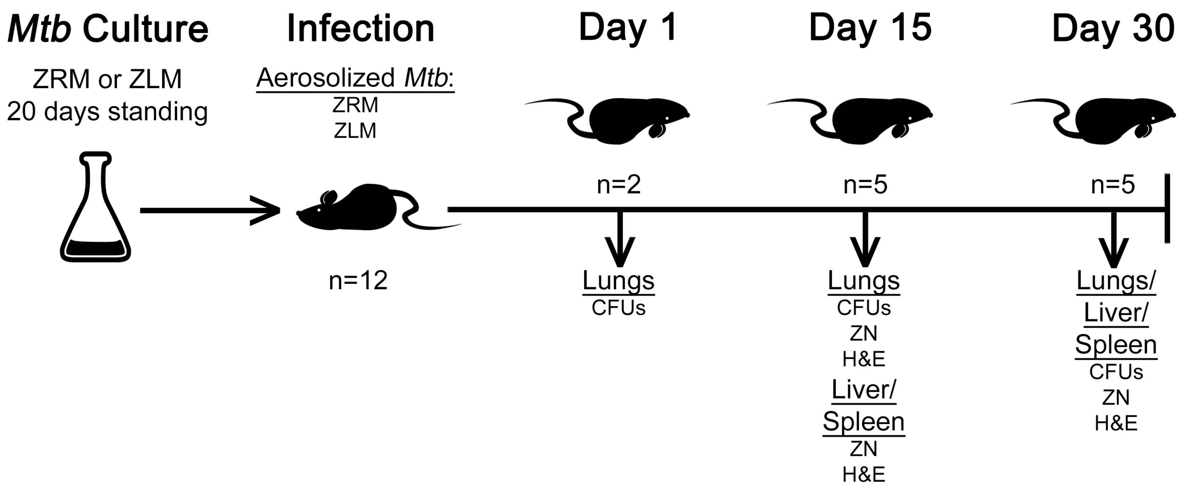

S13 Fig. Schematic of mouse infection experiment with *Mtb* H37Rv pre-grown in ZRM or ZLM. *Mtb* cultures were grown in ZRM or ZLM for 20 days in standing culture before being normalized for aerosol infection of 12 mice per inoculum condition. Prior to conducting the full animal study, conditions were standardized to ensure equal seeding of single cell aerosol particles in the lungs. Bacterial burden in the lungs after 24 hours (1 dpi) was assessed in two mice per condition. After 15 days post-infection (dpi) five mice per condition were sacrificed to determine bacterial burden in the lungs by plating for *Mtb* colony forming units (CFUs). Blinded histopathology analysis was also scored from cross-sections of the lungs, livers and spleens of mice at 15 dpi using Ziehl-Neelsen (ZN) stain for mycobacteria to determine bacterial burden and hematoxylin and eosin (H&E) to score changes in lung morphology and immune cell infiltration. At 30 dpi five mice per inoculum condition were harvested to determine bacterial burden from CFUs in the lungs, livers and spleens. Blinded histopathology scores from ZN and H&E stained cross-sections of the lungs, liver, and spleen were also recorded at 30 dpi. No mice died naturally or from infection during the course of the experiment.
