# Supplementary material for: Zinc limitation triggers anticipatory adaptations in Mycobacterium tuberculosis
Source: PLoS Pathog. 2021 May 14;17(5):e1009570. doi: 10.1371/journal.ppat.1009570 (PMC8121289; doi:10.1371/journal.ppat.1009570)
Supplement: S14 Fig — (PDF) [file ppat.1009570.s014.pdf]

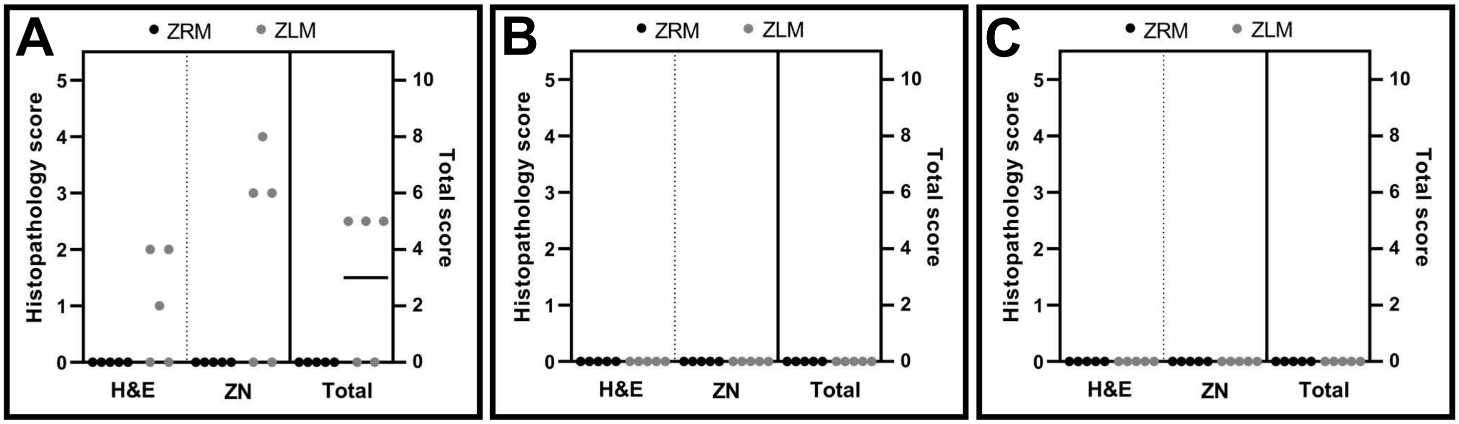

S14 Fig. Histopathology scores from the lungs (A), livers (B), and spleens (C) of mice after 15 days of infection with *Mtb* H37Rv pre-grown in ZRM or ZLM. Blinded histopathology scores from cross-sections of tissues stained with hematoxylin and eosin (H&E) to score changes in lung morphology and immune cell infiltration and Ziehl-Neelsen (ZN) stain for mycobacteria to determine bacterial burden. Maximal scores of five for H&E and ZN stained cross sections (plotted on the left y-axis) are summed to give the total score (right y-axis) with a score of 10 representing the maximal level of disease pathology. The horizontal bar through the data for total score represents the average total pathology score for mice infected with *Mtb* pre-grown in ZRM or ZLM.
