# Supplementary material for: Zinc limitation triggers anticipatory adaptations in Mycobacterium tuberculosis
Source: PLoS Pathog. 2021 May 14;17(5):e1009570. doi: 10.1371/journal.ppat.1009570 (PMC8121289; doi:10.1371/journal.ppat.1009570)
Supplement: S15 Fig — (PDF) [file ppat.1009570.s015.pdf]

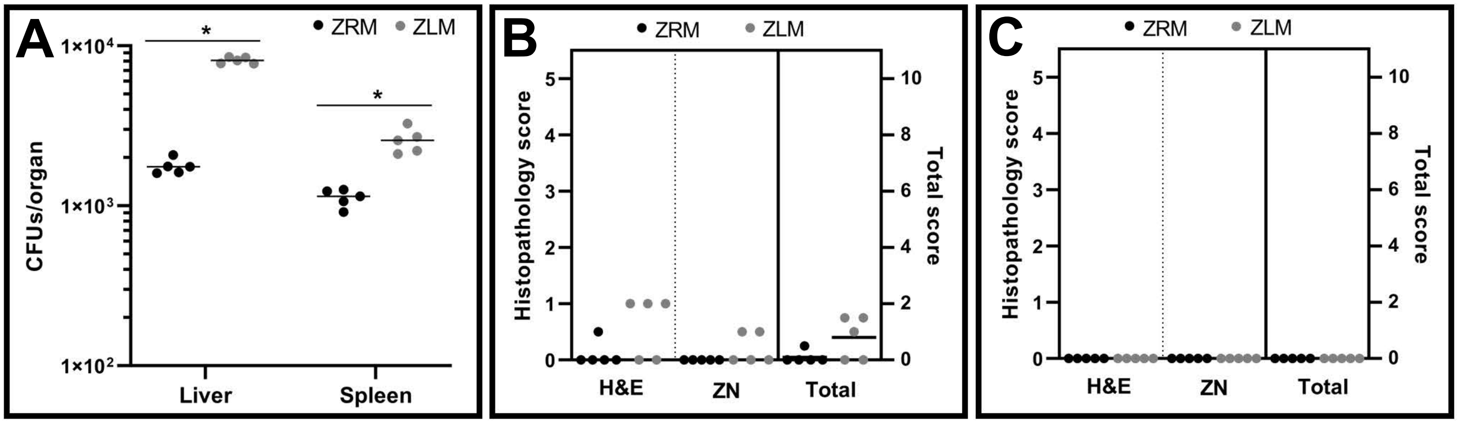

S15 Fig. Bacterial burden and histopathology scores from the livers and spleens of mice after 30 days of infection with *Mtb* H37Rv pre-grown in ZRM or ZLM. (A) Bacterial burden as determined by colony forming units (CFUs) from the livers and spleens of 5 mice per inoculum condition. Horizontal bars across the data points represent the average CFUs (n=5 mice per condition). Asterisks represent a statistically significant difference (t-test, p-value <0.05) between CFUs of mice infected with ZRM vs. ZLM. Blinded histopathology scores from cross-sections of livers (B) and spleens (C) stained with hematoxylin and eosin (H&E) to score changes in lung morphology and immune cell infiltration and Ziehl-Neelsen (ZN) stain for mycobacteria to determine bacterial burden. Maximal scores of five for H&E and ZN stained cross sections (plotted on the left y-axis) are summed to give the total score (right y-axis) with a score of 10 representing the maximal level of disease pathology. The horizontal bar through the data for total score represents the average total pathology score for mice infected with *Mtb* pre-grown in ZRM or ZLM.
