# Supplementary material for: Zinc limitation triggers anticipatory adaptations in Mycobacterium tuberculosis
Source: PLoS Pathog. 2021 May 14;17(5):e1009570. doi: 10.1371/journal.ppat.1009570 (PMC8121289; doi:10.1371/journal.ppat.1009570)
Supplement: S1 Text — (PDF) [file ppat.1009570.s027.pdf]

S1 Text

Zinc limitation triggers anticipatory adaptations in *Mycobacterium tuberculosis*

Allexa Dow, Preeti Sule, Timothy J. O'Donnell, Andrew Burger, Joshua T. Mattila, Brandi Antonio, Kevin Vergara, Endrei Marcantonio, L. Garry Adams, Nicholas James, Philip G. Williams, Jeffrey D. Cirillo, Sladjana Prisic

Materials and Methods

Media

All chemicals were purchased from Fisher Scientific™ or VWR™, unless otherwise noted. Whenever the auxotrophic strain *Mtb* mc^2 6206 was grown, 50 µg/mL L-leucine and 48 µg/mL calcium D-pantothenate were added to the medium. For strain maintenance, *Mtb* was grown in Middlebrook 7H9 (Difco) broth supplemented with ADC-T (0.5% albumin, 0.2% glucose, 0.085% NaCl, 0.05% Tween 80). Chemically defined Sauton's medium (0.05% KH_2PO_4, 0.05% $MgSO_47H_2O$, 0.2% citric acid, 0.005% ferric ammonium citrate, 6% glycerol, 0.4% asparagine, 0.05% Tween 80, pH 7.4) was used for all growth experiments. Zn^{2+} limitation in batch cultures was achieved by omitting $ZnSO_4$ in Sauton's Zn^{2+}-limited medium (ZLM) and for Zn^{2+}-replete medium (ZRM), $ZnSO_4$ was added to ZLM at 6 µM final concentration. Care was taken to avoid Zn^{2+} contamination during preparation of ZLM, only high-quality ultrapure type I water (18.2 MΩ.cm, Milli-Q®) was used and any contact with glassware was avoided during media preparation. ZLM media was prepared using polypropylene beakers and the pH was adjusted with sodium hydroxide prepared fresh with each batch of media in polypropylene conical tubes and the media was filter sterilized using 0.2 µm PES membrane vacuum filtration units (VWR™, Cat. #: 10040-440). Elemental analysis of zinc in ZLM using Inductively Coupled Plasma Mass Spectroscopy (ICP-MS) was performed in the OHSU Elemental Analysis Core.

Detection of calprotectin (CP) in sputum

Sputum samples were processed with NAC-PAC Red reagent and neutralized using the NPC-67 neutralizing buffer (Alpha-Tec). After the centrifugation step, sputum supernatants were heated at 92°C for 30 minutes to inactivate *Mtb* and were stored at -80°C until analysis.

For detection of CP from sputum samples, we used sandwich ELISA. Sheep polyclonal Anti-S100A8 antibody (4μg/mL, R&D Systems, Cat. #: AF4570), the capture antibody, was added to high antibody binding 96-well white polystyrene plates (ThermoFisher Scientific, Cat. #: 15042) and incubated for 16 hours at 4°C. Wells were blocked with phosphate buffered saline with 0.05% Tween 20 and 3% BSA for 4 hours at 22°C. Recombinant calprotectin standards and sputum samples were added to the wells and incubated for 2 hours at 22°C. Mouse monoclonal anti-S100A8 (4 μg/mL, Santa Cruz Biotechnology, Cat. #: SC-48352), the detection antibody, was incubated for 2 hours at 22°C. Goat anti-mouse IgG antibody conjugated to HRP (1 μg/mL, ThermoFisher, Cat. #: A16090) was used as secondary antibody for 1hr at 22°C. SuperSignal™ ELISA Pico Chemiluminescent Substrate was added to the wells (ThermoFisher, cat no: 37069) and luminescence was immediately detected using a BioTek plate reader. Wells were washed three times with PBST between each step.

Strains

Mtb H37Rv strain was a gift from Dr. Robert Husson, Boston Children's Hospital and *Mtb* mc^2 6206 is kindly provided by Dr. William R. Jacobs Jr., Albert Einstein College of Medicine of Yeshiva University. To monitor *altRP* promoter activity during growth, wild type *Mtb* H37Rv and mc^2 6206 were transformed with a plasmid carrying a fluorescent reporter, mCherry, expressed under the *altRP* promoter in an integrating vector (pMV306-P_{altRP}-mCherry), as reported previously [1]. The protein is optimized for expression in mycobacteria and was transferred from pVV16-mCherry plasmid [2]. Fluorescence of mCherry reporter (ex/em λ= 590 nm/635 nm) was measured throughout growth in ZLM in a Tecan plate reader.

Immunocytochemistry and confocal microscopy

Formalin-fixed paraffin-embedded tissue sections from cynomolgus macaques (*Macaca fascicularis*) that were experimentally infected with *Mtb* Erdman strain [3] were processed for immunohistochemistry as previously described [4]. Serial 5 μm-thick sections were stained for 1 hour with anti-CD68 (clone KP1, 1:50 dilution; ThermoFisher Scientific, Waltham, MA, USA), and 1:50 dilutions of polyclonal rabbit anti-S18-1 or anti-S18-2 (NeoBioSci). After washing, tissue sections were incubated with species-specific

secondary antibodies for 1 hour. The following antibodies were used: AlexaFluor546-conjugated donkey anti-rabbit IgG (Jackson ImmunoResearch Laboratories, West Grove, PA) and AlexaFluor488-conjugated donkey anti-mouse IgG (Jackson ImmunoResearch Laboratories). Subsequent staining was performed with calprotectin (clone MAC387; 1:100 dilution; ThermoFisher) antibodies labeled with AlexaFluor647 Zenon-labeling reagents (ThermoFisher). After washing, coverslips were applied with Prolong Gold mounting medium containing DAPI (ThermoFisher) and cured for 24 h before imaging with Fluoview 1000 laser scanning confocal microscope (Olympus, Center Valley, PA) maintained by the University of Pittsburgh's Center for Biologic Imaging. We chose to image lesions from animals with active TB with features that were representative of necrotic granulomas in order to identify subpopulations of bacteria residing within high and low [Zn^{2+}] microenvironments. Three-color images (red, green, and far red [pseudocolored as blue]) were acquired sequentially.

Western blots

For all western blots, *Mtb* lysates are proteins from TRIzol™ extracts, protein samples are resolved on sodium dodecyl sulfate polyacrylamide gels as indicated in the figure legends and transferred onto a Sequi-Blot™ PVDF membrane (Bio-Rad). After transfer, membranes were rinsed 3X with PBS+0.1% Tween-20 (PBST) after each step. Membranes were blocked in PBST+3% BSA for 1 hour at 22°C, Anti-*Mtb* S18-1 and S18-2 primary antibodies were diluted 1:1000 with overnight incubation at 4˚C followed by incubation with secondary antibody (Goat anti-Rabbit IgG (H+L)-HRP) diluted 1:5000 with incubation at 22°C for 1 hour. Anti-*Mtb* KatG (clone IT-57) mouse monoclonal antibody was used as the primary antibody at a 1:1000 dilution with overnight incubation at 4˚C followed by incubation with secondary antibody (Goat anti-Mouse IgG (H+L)-HRP) diluted 1:5000 with incubation at 22°C for 1 hour. The following reagent was obtained through BEI Resources, NIAID, NIH: Monoclonal Anti-Mycobacterium tuberculosis KatG (Gene Rv1908c), Clone IT-57 (CDA4) (culture supernatant), NR-13793 [5]. Membranes were developed with SuperSignal™ West Chemiluminescent Substrate (Thermo Scientific™) Pico substrate for S18-1 and S18-2 antibodies and Fempto substrate for KatG antibody. Chemiluminescence was detected using a SynGene imager.

Preparation of inoculum, seeding flasks and monitoring growth

Seed cultures were grown fresh at the beginning of each experiment by inoculating 1 mL of bacterial stock from -80˚C into 50 mL 7H9/ADC-T in a vented 250 mL flask (TriForest Labware, Cat. #: FPC0250S) as standing culture for 2-3 days, then transferred to shaking culture until the optical density at 600 nm (OD_{600}) reached ~0.8. The amount of seed cells needed to achieve OD 0.08 in 50 mL total volume was calculated and this volume of seed cells was transferred to 50 mL conical tubes. Cells were harvested by centrifugation at 3,000x*g* for 10 minutes at room temperature and re-suspended in 50 mL ZLM and decanted into new 250 mL vented flasks. Once in flasks, 6 µM final concentration of $ZnSO_4$ (1 M stock, 10 mM working) was added to ZRM cultures. Growth was at 37°C with shaking at 120 rpm for the remainder of the experiment. Cultures were monitored for growth by measuring OD_{600}. For *Mtb* H37Rv, OD was measured in a standard 1cm pathlength benchtop spectrophotometer and for *Mtb* mc^2 6206 OD was measured in a microplate reader and a regression equation was used to convert OD measurements from 200 µL sample volume in a 96-well plate to standard 1 cm path length.

Fluorescence measurements for *Mtb* H37Rv were recorded using a filter-based microplate reader (GENios FL, Tecan) while those for *Mtb* mc^2 6206 were recorded using a monochrome microplate reader (infinite 200Pro, Tecan). Fluorescence from mCherry was detected using 580 nm and 635 nm ex/em filters for *Mtb* H37Rv and an ex/em wavelength of 590 nm/635 nm for *Mtb* mc^2 6206. Fluorescence from the redox cofactor F_{420} was monitored in *Mtb* mc^2 6206 using ex/em wavelength of 420 nm/475 nm, or by using excitation wavelength scans from 350nm to 440 nm with a fixed emission wavelength of 475 nm.

Analysis of gene expression in response to different [Zn^{2+}] and Zn^{2+}-chelators

Cells from mid log phase (OD 0.5-0.8) *Mtb* mc^2 6206 cultures were harvested via centrifugation at 3000x*g* for 10 minutes, normalized to OD 0.1 in Sauton's medium with no added Zn^{2+} and incubated in vented culture flasks with shaking at 120 rpm at 37°C for 4 days. On day 5, after detection of mCherry from the reporter strain carrying pMV306-P_{altRP}-mCherry, 3.5 mL aliquots of cells were harvested via centrifugation at 3000x*g* for 10 minutes, and re-suspended in an volume of fresh Sauton's medium without added Zn^{2+} that would give a final volume of 3.5 mL after addition of other chemicals and transferred into PETG media bottles (VWR, Product #: 89132-066), Five conditions representing differing

free [Zn^{2+}] availability in the media were set-up in biological triplicate and these conditions were: 0.35 mg/mL recombinant CP, 0.8 µM TPEN, no added Zn^{2+}, 6 µM Zn^{2+} and 500 µM Zn^{2+}. To all cultures, a final concentration of 2 mM $CaCl_2$ (required for CP chelating ability) and the equivalent volume of DMSO added to the TPEN condition were added to all treatments to eliminate variability across conditions. After adding treatments, OD_{600} was recorded and cultures were incubated at 120 rpm at 37°C for 24 hours. After 24 hours, OD_{600} was recorded and cells were harvested via centrifugation at 3000x*g* for 10 minutes at 4°C and cell pellets were resuspended in 1 mL of TRIzol™. Isolation of RNA, sequencing and DE analysis of gene expression are described below.

Purification of RNA and proteins from *Mtb*

Mtb H37Rv was grown to late-log phase (10 days) in ZLM (n=3) or ZRM (n=3) from a starting OD of 0.05 and Zn^{2+}-limitation was evident as described in the text. Twenty-five mL of each culture was transferred to 50 mL conical tubes and cells were pelleted with centrifugation at 3,000x*g* for 10 minutes at 4˚C. The supernatant was discarded, and cell pellets were resuspended in 1 mL TRIzol™ reagent (Invitrogen™, Cat. #: 15596026) and transferred to a 2 mL screw-cap tube containing 200 µL of 0.1 mm zirconia beads (BioSpec, Cat. #: 11079101z). Tubes were rotated for 30 minutes at room temperature and then frozen at -80˚C until killing was validated and samples were removed from the BSL3 containment facility for further processing. Bacterial cell pellets in TRIzol™ were lysed via beating 3 times for 45 seconds at 7000 rpm in a MagNA Lyser (Roche) with cooling on ice for 3 minutes between cycles. Screw cap tubes were spun at 12,000x*g* for 5 minutes at 4°C and the supernatant was transferred to new 1.5 mL tubes. RNA and proteins were extracted from TRIzol™ supernatants concurrently, following the manufacturer's protocol.

Analysis of gene expression and differentially expressed (DE) genes

Precipitated RNA from the aqueous TRIzol™ fraction was resuspended in nuclease free water and purified using High Pure RNA Isolation kit (Roche, Cat. #: 11828665001) with omission of the on-column DNase digest step. Purified RNA was quantified using the ratio of absorbance at 260 and 280 nm using a Nanodrop™ (Thermo Scientific™) spectrophotometer and digested in solution twice with TURBO™

DNase (Invitrogen™, Cat. #: AM2239) following the manufacturer's directions. DNase-digested RNA was re-purified using the same kit as before. Ribosomal RNA was depleted from the samples using Ribo-Zero plus rRNA depletion kit (Illumina®, Cat. #:20037135) before being sequenced on an Illumina® HiSeq platform for generation of 150 base pair paired end reads. RNA sequencing (RNAseq) was performed by GENEWIZ® (South Plainfield, NJ 07080).

Analysis of raw read data was achieved following the pipeline: fastQC [6] of raw reads in .fastq format, trimmomatic [7] (parameters: HEADCROP:25 CROP:110 LEADING:30 TRAILING:30 SLIDINGWINDOW:4:15 MINLEN:35), fastQC of trimmed reads, Bowtie 2 [8] alignment of trimmed files and featureCounts [9] to define tagwise abundances for coding and non-coding RNAs from BAM files. Reference genome files (.gtf and .fasta) for gene annotation and featureCounts were obtained from Mycobrowser, Release 3 [10]. Data analysis was performed through the command line using the high-performance computing cyberinfrastructure from the University of Hawaii Information Technology Services.

The tagwise abundances were normalized using trimmed means of M-values (TMM) method in edgeR to obtain logCPM values for each feature [11] and the and Quantile-adjusted conditional maximum likelihood (qCML) linear modeling approach of normalized gene abundances was used to determine the common dispersion using limma [12]. Differential gene expression of the linearized model was determined using an "exact-like test" that utilizes a negative binomial distribution model and Benjamini-Hochberg false discovery rate (FDR) approximation to control for the family-wise error rate [12]. Significantly differentially regulated genes (DE genes) were defined as having an absolute fold change (absFC) greater than 2 and an FDR (adjusted P-value) less than 5% (FDR <0.05, absFC>2). A $\log_2$-fold change (logFC) threshold of 1 was applied using *treat* function in limma to ensure the false discovery rate (FDR) is controlled using the Benjamini-Hochberg procedure for multiple testing correction while only considering genes with changes in expression levels above the threshold (absFC >2) [12]. RNA sequence analysis using the edgeR-limma workflow was based on the previously published script [13] and was performed using R version 3.5.3 [14]. Enrichment analysis for gene ontology (GO) terms from DE genes in ZLM was achieved using DAVID with a p-value <0.05 used to define significantly enriched GO terms [15–17]. The circle plot in Figure 2 was created using the R package GOplot version 1.0.2 [18].

The color of the bars in the inner circle is determined by the z-score which indicates whether the given biological process is more likely to be decreased or increased in the dataset and is given as follows:

$$zscore = \frac{(up - down)}{\sqrt{count}}$$

Whereas *up* and *down* are the number of assigned DE genes that are upregulated or downregulated respectively in ZLM and *count* is the total number of genes assigned to a given GO term. Assignment of DE genes to metabolic pathways and the superimposition of DE genes onto the global metabolic network for *Mtb* was achieved using KEGG Mapper tool with organism specific search mode for *Mtb* H37Rv (mtu) [19,20].

Protein profiling using label-free quantitative mass spectrometry (qMS) and differentially expressed proteins (DEPs)

Total cellular protein was isolated from the organic fraction from TRIzol™ extractions above, and proteins were purified as previously reported [1]. Protein pellets were solubilized in 9.5 M urea and 2% CHAPS buffer, pH 9.1 and protein concentration was determined with the DC assay (Bio-Rad). Protein (100 µg) was digested using filter-aided sample preparation (FASP) method [21] and digested peptides were resuspended in 5% acetonitrile/5% formic acid and analyzed by UC Davis Genome Center – Proteomics Core by LC-MS/MS on a Q Exactive™ Plus Orbitrap Mass spectrometer in conjunction with Proxeon Easy-nLC II HPLC (Thermo Scientific) and Proxeon nanospray source following the Core's standard protocol. Buffer A was 0.2% formic acid in water and buffer B was 0.2% formic acid in acetonitrile. Following method was used: flow rate was 2 µl/min, 10 min buffer A, 90 min 5-35% gradient buffer B, 5 min 95% buffer B, 5 min 5% buffer B. Mass spectra in .raw format was converted to .mzXML format using MSConvert from ProteoWizard (ProteoWizard 3.0.19317.0ef6e44d0), tandem mass spectra were extracted, and MS/MS analyzed with X! Tandem (The GPM, thegpm.org; version X! Tandem Alanine (2017.2.1.4)) [22] for protein identification using search parameters: Cysteine alkylation – iodoacetamide; digestion – trypsin; fixed modifications – carbamidomethylation (C); variable modifications – oxidation (M), deamidation (N, Q), phosphorylation (S, T, Y), and acetylation (K); precursor mass tolerance – 20ppm and fragment mass tolerance – 10ppm. Spectral counts were exported as .csv format

using Excel. It has been demonstrated that multidimensional proteomic datasets from label-free quantitation experiments have a mean-dispersion relationship that can be modeled in edgeR, and as such we used the same edgeR-limma workflow applied to the RNAseq dataset for differential expression analysis of the proteome using spectral count data from X! TANDEM, with the exception that an absolute fold change value of 1.5 (absFC >1.5) was used to define DE proteins [13,23]. The mass spectrometry proteomics data have been deposited to the ProteomeXchange Consortium [24] via the PRIDE [25] partner repository with the dataset identifier PXD024389.

Obtaining single cell suspensions of *Mtb*

Considering the clumping nature of cultures in ZRM and ZLM by late-log phase, whenever we used the cells in an assay that required normalization or visualization we processed the culture to enrich for single cell suspensions. First, we transferred the late-log phase (10 days) cultures into 50 mL conical tubes and spun them at 800 x*g* for 10 minutes to remove large clumps. Next, the supernatants from the spin were filtered through 10 µm cell strainers (pluriStrainer®, Cat. #: 43-50010-03). The strained cells were then normalized to OD 0.5 and used for downstream assays or microscopy.

NAD(P)/H quantification assays

Quantification of nicotinamide cofactors were performed on late-log (10 days) cultures from ZRM and ZLM that had been processed to obtain single cell suspensions. Cells were normalized to an OD_{600} of 1 in PBS + 0.05% Tween 80 and 1 mL of cells were pelleted for each assay (1 for the oxidized and 1 for the reduced cofactor for phosphorylated and non-phosphorylated form). Assays were done using EnzyFluo™ NAD(P)/H kits (BioAssay Systems, Cat. #: EFND-100 and Cat. #: EFNP-100) following the manufacturer's directions. Fluorescence was detected using a Tecan plate reader. Statistical significance between ratios of oxidized to reduced forms was determined using a Student's t-test with alpha=0.05 in Excel.

Fluctuation and FLIM/FRET

Cells from late-log phase (day 10) were enriched for single cell suspensions and 2 µL were mounted on a microscope slide under a cover slip and sealed. Fluorescence lifetime imaging microscopy (FLIM)

measurements were recorded using an Alba FCS connected to a Nikon TE2000-U as previously described [26,27]. Two-photon excitation was provided by a Chameleon Ultra (Coherent, Santa Clara, CA) tuned to 740 nm with the NADH emission being spectrally filtered into PMTs through a 680 nm short-pass filter (FF01-680; Semrock, Rochester, NY) and a 450 nm bandpass (Chroma, Bellows Falls, VT). An ISS A320 FastFLIM box was joined to the Ti:Sapphire laser that created 80 fs pulses at a repetition rate of 80 MHz (H7422P-40, Hamamatsu, Hamamatsu City, Japan). NADH in solution, which was prepared each day, was used for standardizing phasor lifetimes at 0.4 ns. SimFCS (obtained from the Laboratory for Fluorescence Dynamics) was used to generate phasor plots of each sample collected and to analyze the pixel distribution of the phasor trajectories. The phasor plot analyzes the lifetime decay information from each pixel of the FLIM micrographs via Fourier transformation to obtain the corresponding phasor position and the resulting 2-D histogram is plotted as a distribution on the phasor plot with coordinates (*g*, *s*) [28].

Lipid extraction, LC/MS and data analysis

Mtb H37Rv cultures were grown in ZRM (n=3) or ZLM (n=3) for 10 days as described above. 25 mL of bacteria were pelleted in 50 mL polypropylene conical tubes and re-suspended in 1 mL methanol. After transferring cell suspensions into 2 mL polypropylene screw cap tubes, samples were mixed on an orbital rotator for 24 hours at room temperature to inactivate bacteria. At this point, bacteria are safe to remove from BSL3 containment. Lipid purification was performed following the previously established protocol [29]. Methanol-cell suspensions were transferred to 50 mL Corex®II tubes (Pyrex®, Cat. #: 8422-50) and 6 mL chloroform and 2mL methanol was added to achieve a final solvent ratio of 2:1. After extraction with rotation for 1 hour, cell suspensions were centrifuged for 10 minutes and the supernatant was collected and dried immediately in Genevac EZ 2 Series evaporator and stored at -20°C before further analysis. Lipids were resuspended in chloroform-methanol (2:1) with 2-3 water washes to remove impurities before being dried in Genevac and re-suspended at 1 mg/mL in chloroform:methanol:water [10:10:3] for LC/MS (Q-TOF) analysis following the previously established procedure [30]. Liquid chromatography separation of lipids was achieved using a flow rate of 0.45 mL/min and a Phenomenex 5 micron Luna C18 column with dimensions 150 x 3.0 mm at 45˚C with a solvent A (methanol:water (99:1)) to solvent B

(IPA:Hexane:water (79:20:1)) gradient of 0-3 mins 50% B, 3-16 mins 50-100% B, 16-20 min 100% B, 20-23 min 100-50% B. Eluting compounds were detected on an Agilent ESI-QTOF 6545 mass spectrometer.

Raw data files were converted to mzML format using MSconvertGUI from ProteoWizard (ProteoWizard 3.0.19317.0ef6e44d0). Converted mzML files were searched using XCMS online using pairwise comparison and default parameters for HPLC/Q-TOF in positive mode except we applied a more stringent mass tolerance of 15ppm m/z deviation in consecutive scans [31]. The compounds detected from LC-MS were identified by cross referencing the detected mass of precursor ions from the raw data with the *Mtb* lipid database [30] using lipid maps [32,33] with a threshold intensity value of 5,000 and a mass error of ±0.01 m/z. We limited searches to the protiated (H^+) form of each adduct and only included identified lipids with absFC>2 and adjusted p-values (q-value) >0.05 in the analysis (S6 Table and Figure 4B). The list of identified lipids was manually curated to remove any compounds that matched multiple lipid identities by retaining the identity of the lipid that was the closest match to the input mass (*i.e.*, had the smallest delta value). Different compounds detected (*i.e.*, features with unique *m/z*) that matched to the same lipid identity were retained as individual data points. The cloud plot of compound abundances was created using XCMS online (Version 3.7.1) [31] and the violin plots are created from the lipids identified with lipid maps using Prism (Version 8.4.0). Abbreviations of selected lipid classes from S6 Table shown in Fig. 4B are as follows: TDM/TMM; trehalose dimycolate and trehalose monomycolate (alpha- and keto-), GMM; glucose monomycolate (alpha-, keto- and methoxy-), TG; triacylglycerols, DG; diacylglycerols, CL; cardiolipins, PIM; phosphatidylinositol monomannosides, AcPIM; acylated (mono- and di-) phosphatidylinositol monomannosides, DIM A/B; phthiocerol (A) and phthiodolone (B) dimycocerosates, Alpha MA; alpha-mycolic acids, Keto MA; keto-mycolic acids, PE/PG/PI; phosphatidylethanolamines, phosphatidylglycerols and phosphatidylinositols.

Scanning electron microscopy

Cells from late-log (day 10) in ZRM (n=3) and ZLM (n=3) were enriched for single cell suspensions and 1 mL of cells from each culture was fixed in 2.5% glutaraldehyde in 0.1 M sodium cacodylate buffer (pH 7.4) for 2 hours. Fixed cells were loaded onto a 0.2 μm polycarbonate Isopore™ membrane filter (Millipore, Cat. #: GTTP01300). Cells on the filter were washed twice in 0.1 M cacodylate buffer for a total

of 30 minutes and post-fixed with 1% OsO_4 in 0.1 M cacodylate buffer for 1 hour. Samples were then dehydrated in a graded 30 to 100% ethanol series with two changes of 5 minutes each and three changes of 100% ethanol for 10 minutes and dried in a Tousimis Samdri-795 critical point dryer. Filters were mounted on aluminum stubs, sputter coated with gold/palladium in a Hummer 6.2 sputter coater and viewed with a Hitachi S-4800 Field Emission Scanning Electron Microscope at an accelerating voltage of 5 kV. Sample preparation and electron microscopy was done at the Biological Electron Microscope Facility (BEMF) at the University of Hawaiʻi at Mānoa.

Antibiotic and plumbagin killing assays

Cells from ZRM and ZLM at late-log phase (day 10) were enriched for single cell suspensions and normalized to an OD_{600} of 0.05 in fresh ZLM. 4 mL of normalized cells were transferred to 15 mL snap-cap polypropylene culture tubes and the appropriate antibiotic or stressor was added to the tubes which were incubated at 37˚C with shaking at 200 rpm for the remainder of the experiment. Stocks were prepared in DMSO unless otherwise noted and stored for up to 6 months at -20˚C. Stock concentrations used were as follows: Plumbagin (100 mM, diluted to 10 mM with DMSO for each experiment), Isoniazid (10 mg/mL), Rifampicin (10 mg/mL), Ethambutol (10 mg/mL) and Kanamycin (50 mg/mL in water, diluted to 5 mg/mL with water for each experiment). Working concentrations were as follows: Plumbagin (50 μM), Isoniazid (15 μg/mL), Rifampicin (30 μg/mL), Ethambutol (200 μg/mL) and Kanamycin (6 μg/mL). For flow cytometry, cells were stained with CellTrace™ Calcein Green-AM (Invitrogen™, Cat. #: C34852) and SYTOX™ AADvanced™ (Invitrogen™, Cat. #: S10349) and analyzed on an Attune NxT flow cytometer following the previously established protocol [34]. 200 μL aliquots of normalized and untreated control cells were immediately stained with C-AM/SYTOX for analysis with flow cytometry. For treated cultures, 200 μL aliquots were taken for six consecutive days and on day 12 after treatment and were stained and analyzed immediately each day. For flow cytometry experiments, percent survival is given as the percentage of live cells (C-AM^+/$SYTOX^-$) in treated cultures compared to the percentage of live cells in the untreated controls. Statistically significant differences in survival at each time point was determined using the Holm-Sidak t-test method for multiple comparisons corrections with alpha=0.05 using Prism (Version 8.4.0).

Animal infection

Cultures for mouse infection were grown in ZRM and ZLM without shaking (standing) with 5% CO_2 for 20 days. Cultures from ZLM (OD_{600} 0.78 and 2.1×10^6 CFUs) or ZRM (OD_{600} 0.9 and 1.55×10^6 CFUs) were normalized to 10^4 CFU/mL and single-cell suspensions were generated to deliver a targeted infection of 50-70 colony forming units (CFU) per mouse. C3HeB/FeJ (Kramnik) mice [35] ordered from Envigo, between 5-7 weeks of age, were infected using a Madison chamber. The Madison chamber enables effective homogenous infection of mice via the aerosol route resulting in minimal variation of bacterial load in the lungs. Mice were randomly divided into 2 groups of 12 each. Post infection (24 hours), two mice from each group were sacrificed and their lungs were harvested, placed in 1 mL sterile PBS, homogenized and plated on 7H11/ADC plates for bacterial enumeration providing the initial seeding. Five mice were further sacrificed on day 15 and day 30 post infection. Lungs, liver and spleen from these mice were processed for bacterial enumeration and histopathology analysis. In brief, half of each organ was homogenized in 1 mL sterile PBS for calculating bacterial load, and half stored in 10% neutral buffered formalin for histopathological analysis. All histopathology analysis was performed by a veterinary pathologist in a completely blinded manner, *i.e.*, the pathologist had no knowledge of the experimental design or any expected outcomes. Statistically significant differences in CFUs at each time point was determined using Student's t-test with alpha=0.05 using Prism (Version 8.4.0).

Ethics statement

Non-human primate (cynomolgus macaque) granuloma samples were obtained from animals that were enrolled in completed studies performed by JoAnne Flynn at the University of Pittsburgh. All work had been previously approved by the University of Pittsburgh's Institutional Animal Use and Care Committee.

All experimental animals (mice) used in this study were approved by the Texas A&M University Institutional Animal Care and Use Committee (#2015-0222). Mice were euthanized by barbiturate overdose using intraperitoneal injection of 100 mg/kg barbiturate. The animals were fed commercial diet (Harlan Teklad, Indianapolis, IN) and provided with ad libitum access to tap water. The mice were monitored daily during the experiment for signs of discomfort or pain.

Leftover sputa used for TB diagnosis were provided by the Hawaii Department of Health State Laboratory. These samples were not collected for research study and did not include any identifiers or any information, other than a positive TB status (confirmed culture positive). Therefore, they were not considered human subject study, as determined by Institutional Review Boards from the University of Hawaii and the Queen's Medical Center, the latter being the site of sputum collection.

SI References

1. Prisic S, Hwang H, Dow A, Barnaby O, Pan TS, Lonzanida JA, et al. Zinc regulates a switch between primary and alternative S18 ribosomal proteins in *Mycobacterium tuberculosis*. Mol Microbiol. 2015;97: 263–280. doi:10.1111/mmi.13022

2. Abramovitch RB, Rohde KH, Hsu F-F, Russell DG. aprABC: a *Mycobacterium tuberculosis* complex-specific locus that modulates pH-driven adaptation to the macrophage phagosome. Mol Microbiol. 2011;80: 678–694. doi:10.1111/j.1365-2958.2011.07601.x

3. Capuano S V., Croix D a, Pawar S, Zinovik A, Myers A, Lin PL, et al. Experimental *Mycobacterium tuberculosis* Infection of Cynomolgus Macaques Closely Resembles the Various Manifestations of Human *M. tuberculosis* Infection. Infect Immun. 2003;71: 5831–5844. doi:10.1128/IAI.71.10.5831-5844.2003

4. Mattila JT, Ojo OO, Kepka-Lenhart D, Marino S, Kim JH, Eum SY, et al. Microenvironments in Tuberculous Granulomas Are Delineated by Distinct Populations of Macrophage Subsets and Expression of Nitric Oxide Synthase and Arginase Isoforms. J Immunol. 2013;191: 773–784. doi:10.4049/jimmunol.1300113

5. Cunningham-Bussel A, Bange FC, Nathan CF. Nitrite impacts the survival of *Mycobacterium tuberculosis* in response to isoniazid and hydrogen peroxide. Microbiologyopen. 2013;2: 901–911. doi:10.1002/mbo3.126

6. Andrews S. FastQC: a quality control tool for high throughput sequence data. Available online at: http://www.bioinformatics.babraham.ac.uk/projects/fastqc. 2010.

7. Bolger AM, Lohse M, Usadel B. Trimmomatic: a flexible trimmer for Illumina sequence data. Bioinformatics. 2014;30: 2114–2120. doi:10.1093/bioinformatics/btu170

8. Langmead B, Salzberg SL. Fast gapped-read alignment with Bowtie 2. Nat Methods. 2012;9: 357–359. doi:10.1038/nmeth.1923

9. Liao Y, Smyth GK, Shi W. featureCounts: an efficient general purpose program for assigning sequence reads to genomic features. Bioinformatics. 2014;30: 923–930. doi:10.1093/bioinformatics/btt656

10. Kapopoulou A, Lew JM, Cole ST. The MycoBrowser portal: A comprehensive and manually annotated resource for mycobacterial genomes. Tuberculosis. 2011;91: 8–13. doi:10.1016/j.tube.2010.09.006

11. Robinson MD, McCarthy DJ, Smyth GK. edgeR: a Bioconductor package for differential expression analysis of digital gene expression data. Bioinformatics. 2010;26: 139–140. doi:10.1093/bioinformatics/btp616

12. Ritchie ME, Phipson B, Wu D, Hu Y, Law CW, Shi W, et al. limma powers differential expression analyses for RNA-sequencing and microarray studies. Nucleic Acids Res. 2015;43: e47–e47.

doi:10.1093/nar/gkv007

13. Law CW, Alhamdoosh M, Su S, Dong X, Tian L, Smyth GK, et al. RNA-seq analysis is easy as 1-2-3 with limma, Glimma and edgeR. F1000Research. 2018;5: 1408. doi:10.12688/f1000research.9005.3

14. R Core Team. R: A language and environment for statistical computing. R Foundation for Statistical Computing, Vienna, Austria. Available online at https://www.R-project.org/. 2019. Available: https://www.r-project.org/

15. Huang DW, Sherman BT, Lempicki RA. Systematic and integrative analysis of large gene lists using DAVID bioinformatics resources. Nat Protoc. 2009;4: 44–57. doi:10.1038/nprot.2008.211

16. Huang DW, Sherman BT, Lempicki RA. Bioinformatics enrichment tools: paths toward the comprehensive functional analysis of large gene lists. Nucleic Acids Res. 2009;37: 1–13. doi:10.1093/nar/gkn923

17. Ashburner M, Ball CA, Blake JA, Botstein D, Butler H, Cherry JM, et al. Gene Ontology: tool for the unification of biology. Nat Genet. 2000;25: 25–29. doi:10.1038/75556

18. Walter W, Sánchez-Cabo F, Ricote M. GOplot: an R package for visually combining expression data with functional analysis. Bioinformatics. 2015;31: 2912–2914. doi:10.1093/bioinformatics/btv300

19. Kanehisa M. KEGG: Kyoto Encyclopedia of Genes and Genomes. Nucleic Acids Res. 2000;28: 27–30. doi:10.1093/nar/28.1.27

20. Kanehisa M, Sato Y, Furumichi M, Morishima K, Tanabe M. New approach for understanding genome variations in KEGG. Nucleic Acids Res. 2019;47: D590–D595. doi:10.1093/nar/gky962

21. Wiśniewski JR, Rakus D. Multi-enzyme digestion FASP and the 'Total Protein Approach'-based absolute quantification of the *Escherichia coli* proteome. J Proteomics. 2014;109: 322–331. doi:10.1016/j.jprot.2014.07.012

22. Craig R, Beavis RC. TANDEM: matching proteins with tandem mass spectra. Bioinformatics. 2004;20: 1466–1467. doi:10.1093/bioinformatics/bth092

23. Branson OE, Freitas MA. Tag-Count Analysis of Large-Scale Proteomic Data. J Proteome Res. 2016;15: 4742–4746. doi:10.1021/acs.jproteome.6b00554

24. Deutsch EW, Bandeira N, Sharma V, Perez-Riverol Y, Carver JJ, Kundu DJ, et al. The ProteomeXchange consortium in 2020: enabling 'big data' approaches in proteomics. Nucleic Acids Res. 2019;48: 1145–1152. doi:10.1093/nar/gkz984

25. Perez-Riverol Y, Csordas A, Bai J, Bernal-Llinares M, Hewapathirana S, Kundu DJ, et al. The PRIDE database and related tools and resources in 2019: improving support for quantification data. Nucleic Acids Res. 2019;47: D442–D450. doi:10.1093/nar/gky1106

26. Nguyen H, Ward WS, James NG. Spatial and temporal resolution of mORC4 fluorescent variants reveals structural requirements for achieving higher order self-association and pronuclei entry. Methods Appl Fluoresc. 2019;7: 035002. doi:10.1088/2050-6120/ab0f57

27. Sanstrum BJ, Goo BMSS, Holden DZY, Delgado DD, Nguyen TPN, Lee KD, et al. Fluctuation Imaging of LRRK2 Reveals that the G2019S Mutation Alters Spatial and Membrane Dynamics. Molecules. 2020;25: 2561. doi:10.3390/molecules25112561

28. Bhattacharjee A, Datta R, Gratton E, Hochbaum AI. Metabolic fingerprinting of bacteria by fluorescence lifetime imaging microscopy. Sci Rep. 2017;7: 3743. doi:10.1038/s41598-017-04032-w

29. Layre E, Sweet L, Hong S, Madigan CA, Desjardins D, Young DC, et al. A Comparative

Lipidomics Platform for Chemotaxonomic Analysis of *Mycobacterium tuberculosis*. Chem Biol. 2011;18: 1537–1549. doi:10.1016/j.chembiol.2011.10.013

30. Sartain MJ, Dick DL, Rithner CD, Crick DC, Belisle JT. Lipidomic analyses of *Mycobacterium tuberculosis* based on accurate mass measurements and the novel " Mtb LipidDB." J Lipid Res. 2011;52: 861–872. doi:10.1194/jlr.M010363

31. Tautenhahn R, Patti GJ, Rinehart D, Siuzdak G. XCMS Online: A Web-Based Platform to Process Untargeted Metabolomic Data. Anal Chem. 2012;84: 5035–5039. doi:10.1021/ac300698c

32. Fahy E, Subramaniam S, Brown HA, Glass CK, Merrill AH, Murphy RC, et al. A comprehensive classification system for lipids. J Lipid Res. 2005;46: 839–862. doi:10.1194/jlr.E400004-JLR200

33. Fahy E, Subramaniam S, Murphy RC, Nishijima M, Raetz CRH, Shimizu T, et al. Update of the LIPID MAPS comprehensive classification system for lipids. J Lipid Res. 2009;50: S9–S14. doi:10.1194/jlr.R800095-JLR200

34. Hendon-Dunn CL, Doris KS, Thomas SR, Allnutt JC, Marriott AAN, Hatch KA, et al. A Flow Cytometry Method for Rapidly Assessing *Mycobacterium tuberculosis* Responses to Antibiotics with Different Modes of Action. Antimicrob Agents Chemother. 2016;60: 3869–3883. doi:10.1128/AAC.02712-15

35. Driver ER, Ryan GJ, Hoff DR, Irwin SM, Basaraba RJ, Kramnik I, et al. Evaluation of a Mouse Model of Necrotic Granuloma Formation Using C3HeB/FeJ Mice for Testing of Drugs against *Mycobacterium tuberculosis*. Antimicrob Agents Chemother. 2012;56: 3181–3195. doi:10.1128/AAC.00217-12
